# Meta-Analysis of Placental Transcriptome Data Identifies a Novel Molecular Pathway Related to Preeclampsia

**DOI:** 10.1371/journal.pone.0132468

**Published:** 2015-07-14

**Authors:** Miranda van Uitert, Perry D. Moerland, Daniel A. Enquobahrie, Hannele Laivuori, Joris A. M. van der Post, Carrie Ris-Stalpers, Gijs B. Afink

**Affiliations:** 1 Reproductive Biology Laboratory, Academic Medical Center, Amsterdam, the Netherlands; 2 Bioinformatics Laboratory, Department of Clinical Epidemiology, Biostatistics and Bioinformatics, Academic Medical Center, Amsterdam, Amsterdam, the Netherlands; 3 Department of Epidemiology, University of Washington, Seattle, WA, United States of America; 4 Medical Genetics and Obstetrics and Gynaecology, and the Institute for Molecular Medicine Finland, University of Helsinki and Helsinki University Hospital, Finland; 5 Women’s and Children’s Clinic, Academic Medical Center, Amsterdam, the Netherlands; VU University Medical Center, NETHERLANDS

## Abstract

Studies using the placental transcriptome to identify key molecules relevant for preeclampsia are hampered by a relatively small sample size. In addition, they use a variety of bioinformatics and statistical methods, making comparison of findings challenging. To generate a more robust preeclampsia gene expression signature, we performed a meta-analysis on the original data of 11 placenta RNA microarray experiments, representing 139 normotensive and 116 preeclamptic pregnancies. Microarray data were pre-processed and analyzed using standardized bioinformatics and statistical procedures and the effect sizes were combined using an inverse-variance random-effects model. Interactions between genes in the resulting gene expression signature were identified by pathway analysis (Ingenuity Pathway Analysis, Gene Set Enrichment Analysis, Graphite) and protein-protein associations (STRING). This approach has resulted in a comprehensive list of differentially expressed genes that led to a 388-gene meta-signature of preeclamptic placenta. Pathway analysis highlights the involvement of the previously identified hypoxia/HIF1A pathway in the establishment of the preeclamptic gene expression profile, while analysis of protein interaction networks indicates CREBBP/EP300 as a novel element central to the preeclamptic placental transcriptome. In addition, there is an apparent high incidence of preeclampsia in women carrying a child with a mutation in CREBBP/EP300 (Rubinstein-Taybi Syndrome). The 388-gene preeclampsia meta-signature offers a vital starting point for further studies into the relevance of these genes (in particular CREBBP/EP300) and their concomitant pathways as biomarkers or functional molecules in preeclampsia. This will result in a better understanding of the molecular basis of this disease and opens up the opportunity to develop rational therapies targeting the placental dysfunction causal to preeclampsia.

## Introduction

Preeclampsia, defined as the development of high blood pressure in combination with proteinuria after 20 weeks of gestation, is a common (incidence 2–8%) and potentially severe pregnancy complication for both mother and child [1]. At present, apart from delivery of placenta and fetus, there is no definite treatment for preeclampsia. Although preeclampsia clinically manifests during the second half of gestation, it most likely originates at the time the early placenta develops.

The molecular mechanisms that play a role in the development of preeclampsia are largely unknown. Over the past decades, much research has been dedicated to identify preeclampsia-specific molecules that can either serve as a biomarker for prognostic/diagnostic purposes, or that play a functional role in the disease and hence are potential therapeutic targets. The main approach that has been used for this purpose is the analysis of gene expression in the placenta of normotensive and preeclamptic pregnancies. This has resulted in the identification of factors unquestionably associated with preeclampsia, such as soluble Vascular Endothelial Growth factor receptor 1 (sFLT1) [2] and soluble Endoglin [3].

We have recently performed a systematic review and meta-analysis on 30 studies reporting lists of differentially expressed genes (DEGs) in relation to preeclamptic placenta [4]. This gene list-driven approach resulted in a 40-gene preeclamptic signature that, apart from identifying most of the genes known to be associated with preeclampsia, singled out 14 genes not previously associated with preeclampsia. Although this literature-based approach resulted in an overall picture of genes differentially expressed in the preeclamptic placenta and in the identification of potential novel biomarkers, the number of genes included in the meta-signature was limited due to a high degree of between-study variability. Apart from the fact that focus of the individual research groups may have driven the reported gene lists, inconsistencies in the preeclampsia-specific gene signatures are largely due to the relatively limited sample size used in most studies, combined with differences in experimental approach, and bioinformatics and statistical methods. Recently, two papers described meta-analyses of preeclampsia RNA microarray studies. Although these studies by their nature increase the sample size and resolve some of the disadvantages of the reported gene list-driven meta-analysis, they do not, or only partly, deal with aspects like quality assessment and uniformity of data analysis [5, 6].

For the current study we retrieved the original microarray data from 11 independent placental mRNA microarray experiments. Microarray data were subjected to quality analysis, pre-processed and analyzed using standardized bioinformatics and statistical procedures. This resulted in a comprehensive preeclampsia gene signature, which upon further analysis led to the identification of a novel pathway associated with preeclampsia.

## Materials and Methods

### Dataset acquisition

Datasets were identified by systematic review [4], searching the NCBI Gene Expression Omnibus [7] and the EBI ArrayExpress [8] functional genomics data repositories using the term “preeclampsia” and close derivatives. Datasets from platforms that interrogated more than 10,000 genes and did not completely pool samples were selected. In addition, we generated one dataset in our laboratory [9]. If available, data was directly obtained from the data repositories. For studies identified by systematic review without publicly available data, authors were contacted to provide original data. An overview of datasets is shown in Tables 1 and 2.

**Table 1:** Datasets included in the meta-analysis.

		number of samples					
		reported		after QC			
Data Set	Data source (first author)	NT	PE	NT	PE	Platform	probes with Gene ID
**1**	Authors^c^ (Centlow) [10]	20	15	19	11	Custom, OPERON	13010
**2**	Authors^$^ (Enquobahrie) [11]	18	18	15	16	Custom, OPERON	13010
**3**	Authors^c^ (Hoegh) [12]	3^a^	3^a^	3^a^	3^a^	Affymetrix HG U133A	19955
**4**	GSE54618 (Jebbink) [9]	12	12	11	11	Illumina HumanHT-12 v4	37463
**5**	Authors^c^ (Kivinen) [13]	6	8	6	7	Affymetrix HG U133 plus 2.0	41245
**6**	GSE30186 (Meng) [14]	6	6	6	5	Illumina HumanHT-12 v4	37463
**7**	GSE4707 (Nishizawa) [15]	4	10	2^b^	7^b^	Agilent Whole Genome Oligo Microarray G4112A	29777
**8**	GSE24129 (Nishizawa) [16]	8	8	8	8	Affymetrix Human Exon 1.0 ST array	21929
**9**	GSE10588 (Sitras) [17]	26	17	24	16	ABI Human Genome Survey Microarray v.2.0	26396
**10A**	GSE25906 (Tsai) [18]	8	7	8	7	Illumina Human-6 v2	27303
**10B**		29	16	28	16		
**11**	GSE14722 (Winn) [19]	11	12	9	9	Affymetrix HG U133A	19955
	**Total**	**151**	**132**	**139**	**116**		

GSE = GEO accession number, NT = normotensive, PE = preeclampsia, QC = Quality Control

^a^each sample represents 3 pooled placentas

^b^samples that are also present in dataset 8 were excluded from dataset 7

^c^original data was provided by the authors.

**Table 2:** Studies excluded in the meta-analysis.

	number of samples		
First author	NT	PE	Platform^a^
Ahr [20]	4	3	University Health Network Hu19k2 (cDNA)
Guller [21]	6	3	Affymetrix HG U133 plus 2.0
Jarvenpaa [22]	3	2	Affymetrix HG U133 plus 2.0
Kang [23]	16	17	Codelink Human Whole Genome Bioarrays
Lee [24]	13	13	Agilent Human 4X44K Oligomicroarrays Chip
Liu [25]	28	24	CapitalBio, OPERON
Mayor-Lynn [26]	6	3	Illumina HumRef-12 v3
Varkonyi [27]	10	12	Agilent Human 4X44K Oligomicroarrays Chip
Zhou [28]	5	5	CapitalBio, OPERON
	**91**	**82**	

NT = normotensive, PE = preeclampsia

^a^platform names were obtained from description in cited publication.

### Data processing and meta-analysis

The meta-analysis was performed according to the stepwise approach described in [29]. In brief, original data was retrieved where possible and subsequently pre-processed using state-of-the art methods for each of the platforms included. Samples of low quality were removed using the quality criteria calculated by the R package arrayQualityMetrics. Effect sizes (Hedges’ g) and their variances were calculated for each gene in each dataset. Dataset-specific effect sizes were combined using an inverse-variance random-effects model. As a final step, a leave-one-out-analysis was performed to increase the robustness of the resulting meta-signature. Details of the analysis are provided as Supplementary Methods in S1 text.

STRING 9.1 [30] was used for protein-protein association analysis. All 388 genes from the meta‐signature were used as input for STRING analysis and a network was built based on high confidence (0.8) evidence from experimental protein‐protein interaction and curated databases.

KEGG Pathway analysis was performed using Graphite [31]. The 388 gene meta-signature was used as input. Pathways were considered significantly enriched at q-values ≤ 0.05.

GSEAPreranked was used for gene set enrichment analysis [32]. All 8612 genes of the meta-analysis were ranked according to their mu-value and used as ranked gene list. Enrichment scores were calculated based on the standard GSEAPreranked settings and the curated gene set database (c2.all.v5.0.entrez.gmt, 4530 gene sets). Gene sets were considered significantly enriched at FDR q-values ≤ 0.05.

Ingenuity Pathway Analysis (Qiagen) Upstream Regulator Analysis was performed to identify upstream regulators that can explain the observed 388 gene meta-signature. Analysis was performed using standard settings. The resulting activation z-score was used to infer the activation states of the predicted regulator. A z-score of > 2 (activated) or <-2 (inhibited) was considered significant.

Gene Expression Barcode: RNA microarray data for human placentas of different gestational ages was obtained from the NCBI Gene Expression Omnibus (accession GSE9984). Samples were pre-processed and barcoded as described by the Gene Expression Barcode 3.0 using the R package frma [33]. Probesets were mapped to official gene symbols using the R package hgu133plus2.db

## Results

For the current meta-analysis, we included 11 different studies representing a total of 151 normotensive and 132 preeclamptic placenta samples (Table 1). Nine studies (covering 91 normotensive and 82 preeclamptic samples) were not included (Table 2) because authors did not respond to our request for data (5 instances) or because authors did not wish to share their data (3 instances). One author was not able to recover the original data files.

**Fig 1:**
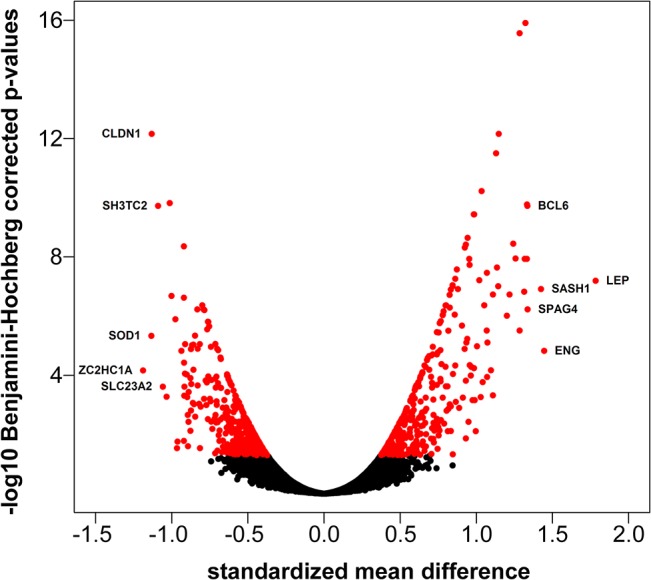
Volcano plot of DEGs. Volcano plot showing the standardized mean difference and the adjusted p-value for the 8,612 genes included on all platforms. The red dots represent genes differentially expressed (adjusted P<0.05) in the preeclamptic versus normotensive placenta. The symbol-marked dots indicate the five genes with the largest negative or positive standardized mean difference.

Quality analysis of the microarray data resulted in the removal of 28 samples. One dataset [18] consisted of two batches, which induced systematic differences in gene expression levels and that were therefore treated as two independent datasets. Meta-analysis was performed on 12 datasets (covering placentas from 139 normotensive and 116 preeclamptic pregnancies) combining the effect sizes using an inverse-variance random-effects model. The meta-analysis was performed on the 8,612 genes available on all platforms, resulting in the identification of 688 differentially expressed genes in preeclamptic versus normotensive placenta (Fig 1). To increase the robustness of the meta-signature, an additional leave-one-out-analysis was performed which reduced the number of genes to 388 (Benjamini-Hochberg-based corrected p-value<0.05), of which 214 were overexpressed in preeclamptic placentas compared to placentas from normotensive pregnancies. A detailed list of the 388 genes and the corresponding statistics is shown in S1 Table, corresponding forest plots are shown in S1 Fig.

Comparison of the current 388-gene meta-signature with the gene list-driven signature of 40 genes we previously obtained from published gene lists reported as being differentially expressed in preeclamptic placenta [4] shows that most of the literature-based genes are also included in the 388-gene signature (S2 Table). In total, 12 genes from the gene list-driven signature were not included in the meta-signature from our current analysis. Six of these were not represented by a gene-specific probe on some platforms (*INHBA*, *CGB*, *F5*, *HTRA4*, *PVRL4*, *RDH13*). Calculation of the standardized mean difference (mu) based on the studies that did contain a gene-specific probe for these six genes shows that all are differentially expressed. Two of the 12 genes (*HSD17B1*, *VIM*) were among the 688 differentially expressed genes in the initial meta-signature, but were removed in the leave-one-out-analysis, and four genes (*CGA*, *IGFBP1*, *PGF*, *TNFSF10*) were not significantly differentially expressed in our meta-analysis on the original microarray data. For those genes in the gene list-driven signature that were also differentially expressed in the current analysis, the direction of differential expression was identical except for *SOD1*, *SPP1* and *VIM*. *SOD1* and *VIM*, which have been described in the literature both as being upregulated and downregulated in the preeclamptic placenta, are definitely downregulated in the current analysis. In fact, *SOD1* is one of the most strongly downregulated genes in the preeclamptic placenta, as shown in S1 Table. The only genuine discrepancy is the direction of *SPP1*. Although reported as being upregulated in the preeclamptic placenta in three different publications, our current meta-analysis identifies *SPP1* as a gene downregulated in preeclampsia.

**Fig 2:**
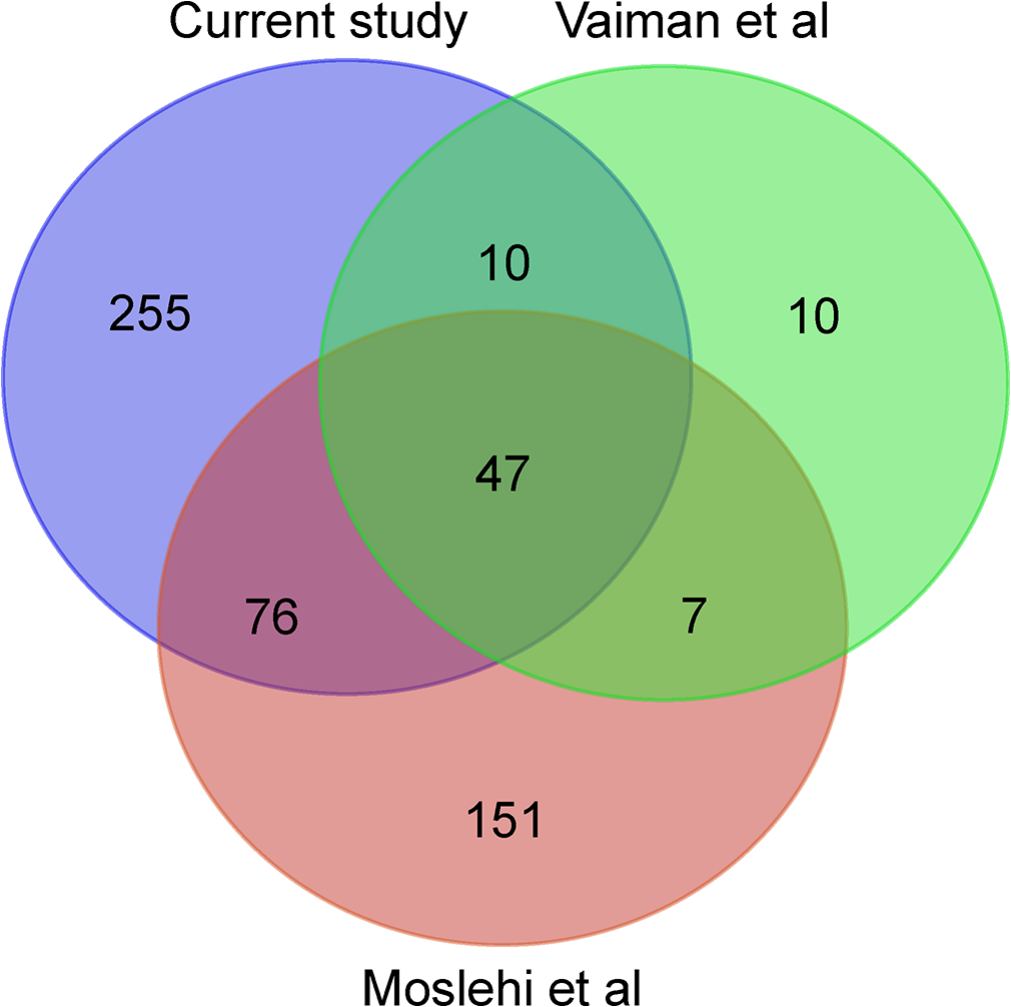
Overlapping genes with previous meta-analyses. Venn diagram of the DEGs from the current study and the previously published studies. DEGs were extracted from the publications [5, 6] and only genes within the gene universe of our study were included in the comparison.

Two recent studies also performed a meta-analysis on microarray data exclusively. Vaiman et al [6] analyzed six datasets (corresponding to dataset 6, 7, 8, 9, 10 and 11 in this paper) and used vote counting to identify genes that were consistently upregulated or downregulated in the majority of studies. Moslehi et al [5] analyzed four datasets (corresponding to dataset 7, 8, 9, and 11 in this paper) and performed their meta-analysis by averaging p-values and fold differences. After removal of the genes not included in our 8,612 gene universe, 77% (57 out of 74) of the DEGs reported by Vaiman et al are included in our current 388-gene meta-signature, while the overlap with the Moslehi study is 44% (123 out of 281) as shown in Fig 2.

**Fig 3:**
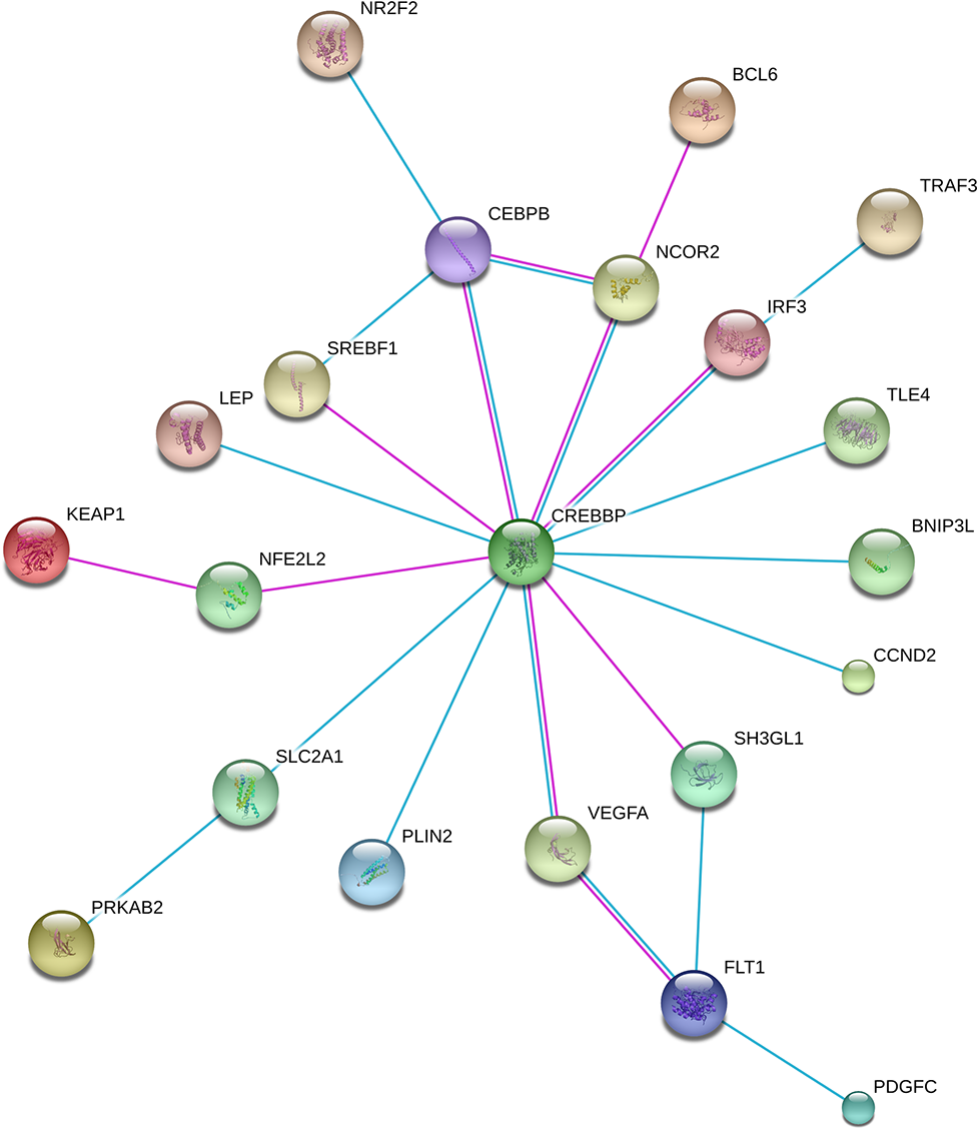
Protein interaction network analysis indicates a central role for CREBBP in the preeclamptic placenta. All 388 genes were used as input for STRING analysis and a network was built (S2 Fig). Shown are the details of CREBBP interactions based on high confidence (0.8) evidence from experimental protein-protein interaction (blue lines) and curated (purple lines) databases. Proteins are indicated by nodes labeled with the encoding gene symbol. The network is enriched in interactions (p = 2.17E-13) [30].

To further identify preeclampsia-related pathways or networks within our 388-gene meta-signature, the STRING network of both physical and functional interactions between proteins was used [30]. By employing a stringent confidence score of 0.8 and experiments plus curated databases as active prediction methods, 112 interactions between proteins encoded by meta-signature genes were observed (S2 Fig). CREBBP is a hub in this network, with 13 direct protein interactions and 23 interactions in total (Fig 3).

**Fig 4:**
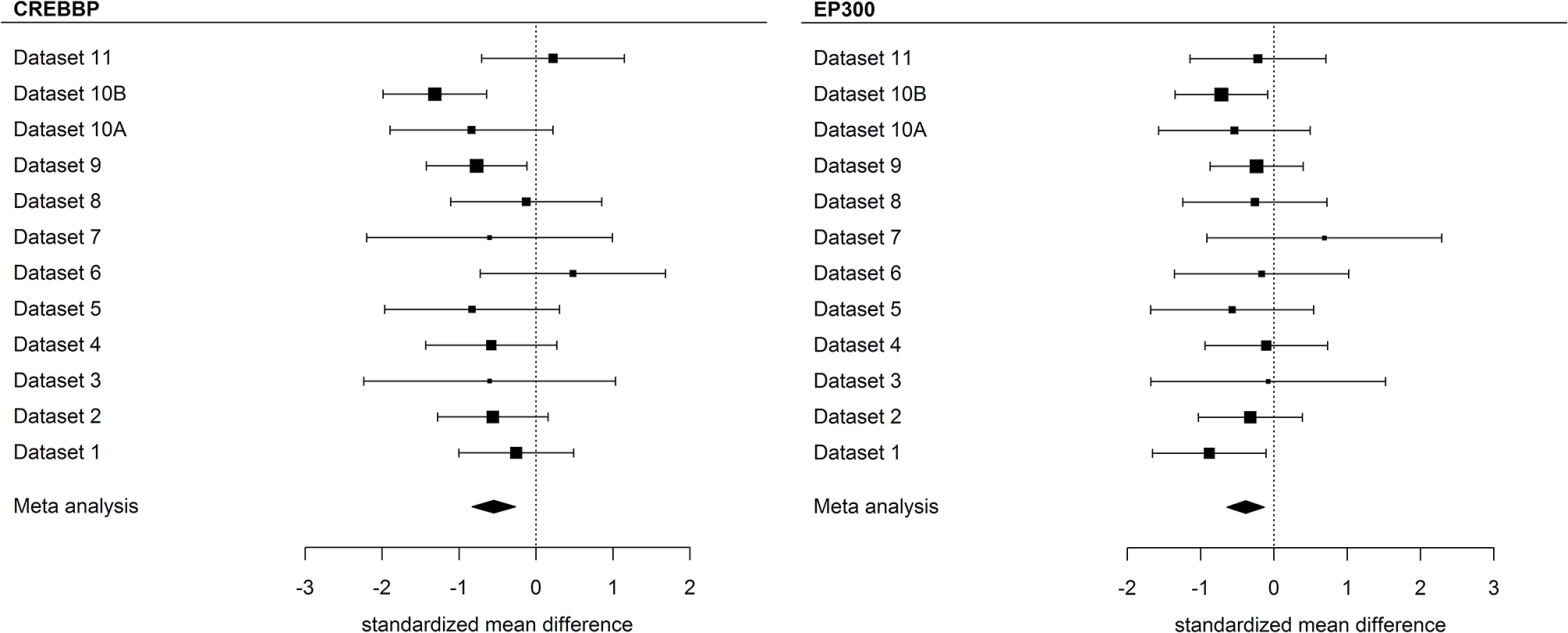
CREBBP/EP300 expression in individual studies. Forest plots for CREBBP and EP300. Squares are proportional to weights used in the meta-analysis, and the lines represent the 95% confidence interval. The diamond represents the standardized mean difference (log2 scale).

As shown in Fig 4, CREBBP (adjusted p-value = 0.004, mu = -0.549) and its functionally similar paralogue EP300 (adjusted p-value = 0.038, mu = -0.388; present in the 688 differentially expressed gene list, but removed in leave-one-out-analysis) [34], are consistently downregulated in almost all individual datasets.

Parallel approaches using pathway analysis resulted in a large number of pathways that are enriched in the 388-gene meta-signature with a consistent enrichment for the hypoxia/HIF1A pathway in Ingenuity Pathway Analysis, Graphite, and Gene Set Enrichment Analysis (Table 3).

**Table 3:** Pathway analysis.

**Ingenuity Upstream Regulator Analysis** ^**a**^				
**Upstream Regulator**	**Molecule Type**		**Predicted Activation State**	**Activation z-score**
HIF1A	transcription regulator		Activated	4.163
PDGF BB	complex		Activated	3.400
CREB1	transcription regulator		Activated	3.392
PPARG	ligand-dependent nuclear receptor		Activated	3.085
CSF3	cytokine		Activated	2.961
**Graphite Pathway Analysis**				
**Pathway**		**q-value**		
HIF-1 signaling pathway		0.0000		
Fc gamma R-mediated phagocytosis		0.0003		
**Gene Set Enrichment Analysis (top of the ranked gene list)** ^**a**^				
**Gene Set Name**		**Size** ^**b**^	**NES** ^**c**^	**FDR q-val**
SEMENZA_HIF1_TARGETS		31	2.517	0.000
ELVIDGE_HYPOXIA_BY_DMOG_UP		109	2.470	0.000
MENSE_HYPOXIA_UP		70	2.461	0.000
LEONARD_HYPOXIA		41	2.450	0.000
ELVIDGE_HIF1A_AND_HIF2A_TARGETS_DN		83	2.437	0.000
ELVIDGE_HYPOXIA_UP		140	2.398	0.000
ELVIDGE_HIF1A_TARGETS_DN		72	2.301	0.000
WANG_ADIPOGENIC_GENES_REPRESSED_BY_SIRT1		17	2.253	0.000
WINTER_HYPOXIA_METAGENE		187	2.240	0.001
FARDIN_HYPOXIA_11		25	2.212	0.001

^a^ For the Graphite Pathway Analysis all the significant pathways are shown. For Ingenuity Upstream Regulator Analysis the top 5 activated upstream regulators, and for Gene Set Enrichment Analysis the top 10 gene sets overrepresented at the top of the meta-signature are shown. A full list of all significant results is presented in S3 Table.

^b^Size: Number of genes in the gene set after filtering out those genes not in the expression dataset.

^c^NES: Normalized enrichment score.

To obtain information if the genes listed in the meta-signature are also expressed earlier during gestation, we performed a Gene Expression Barcode analysis on a microarray data set including 1^st^ and 2^nd^ trimester placentas [35]. As shown in S4 Table most of the meta-signature genes are expressed above background (z-score > 3 in 2^nd^, and even 1^st^ trimester placenta.

## Discussion

We established a robust 388-gene meta-signature in what is presently the most complete meta-analysis of the preeclamptic placental transcriptome. Our meta-analysis not only greatly increases the number of samples included as compared to the individual studies, but also largely resolves the between-study variation in bioinformatics and statistical analysis, which was one of the major inconsistencies reported in our previous gene list-driven meta-analysis [4].

Recently, several other approaches to summarize placental mRNA expression data in relation to preeclampsia have been reported. As mentioned above, we previously performed a systematic review and vote-counting-based meta-analysis on published preeclampsia lists of DEGs from both genome-wide microarray and smaller scale mRNA expression studies [4]. Most genes in the resulting gene list-driven meta-signature are also included in our current 388-gene meta-signature. Genes not included in our current meta-signature had a lower number of votes and mainly originate from older studies. The current meta-analysis clearly generates many additional DEGs compared with the literature-based approach.

The 388-gene meta-signature we currently present shows considerable overlap (77%; odds ratio = 82.86, P<2.2·10^−16^, Fisher’s exact test) with the genes in the meta-signature of Vaiman and colleagues, and with the meta-signature reported by Moslehi and colleagues (44%; odds ratio = 23.67, P<2.2·10^−16^, Fisher’s exact test). However, our current approach has generated many more DEGs. Possible reasons for this are multiple and include (i) an increase in statistical power by the larger number of studies included in our meta-analysis, (ii) renormalization of the original data using an uniform set of state-of-the-art methods whenever possible, and (iii) use of stringent quality control criteria to detect and discard low-quality arrays. Differences in meta-analysis techniques used also impact the meta-signatures obtained. Combining effect sizes using an inverse-variance random-effects model has several advantages compared to the meta-analysis techniques used by the other two studies [29]. Particularly important is the incorporation of between-study heterogeneity into the study weights, since the 11 studies included in our meta-analysis are geographically (eight different countries) and technically (eight different platforms, 10 different laboratories) diverse.

Higher statistical power of the current meta-analysis compared to the individual studies leads to discovery of novel genes/pathways as illustrated by CREBBP (Fig 4). *CREBBP* is downregulated in preeclamptic placenta in most studies, but with wide confidence intervals. However, its association with preeclampsia is exposed by combining all studies in the current meta-analysis.

There are two obvious issues that have limited the full potential of the current analysis. (i) Of the 20 suitable studies identified, only seven had submitted their data to a publicly available repository; the data of four additional studies were obtained directly from the authors. Although the acquired original data represents the majority of samples, it is disappointing that from almost half of the studies data did not become available.

(ii) In order to obtain summary statistics and p-values based on data from all studies, only the genes present on all platforms were taken into account. This results in a large decrease of the gene universe in the current analysis (8,612 genes) compared to the more than 20,000 genes located on most of the microarray platforms included. In particular, the inclusion of the Operon platforms (representing approximately 13,000 Entrez gene IDs) dramatically decreases the number of genes in the intersection.

A shortcoming of the current meta-analysis (but also for all preeclampsia placental gene expression studies) in relation to both function of gene products and their potential role as predictors in preeclampsia is the timing of the gene expression pattern. All mRNA microarray data included in the current meta-analysis are generated from placentas at delivery, while the initial defects leading to preeclampsia occur earlier during pregnancy [1, 36]. Based on data from one of the few microarray data sets on early gestation placenta, we show that the majority of the meta-signature genes is expressed during the 1^st^ and 2^nd^ trimester. CREBBP shows clear placental expression from 1^st^ trimester onwards, but the level of EP300 mRNA expression in the early placenta is less pronounced. Novel developments allowing the quantification of placental RNAs in maternal blood offers the enticing opportunity to study placental gene expression in ongoing pregnancy [37].

A further asset would be the coupling of individual gene expression data to more extensive clinical parameters. The method and time of delivery and the use of antihypertensive drugs, among other things, are likely to affect placental gene expression. Preeclampsia, as a heterogeneous condition consists of several subtypes with different etiology and degree of placental contribution [38]. Unfortunately, most of the datasets available for the current study are not accompanied by sufficient individual clinical information to allow more detailed placental transcriptome to clinical phenotype coupling.

Identification of functionally relevant pathways from the large number of genes in the preeclampsia-placental signature is a major challenge. Building networks based on protein-protein interactions can help to identify these functionally relevant pathways. Even when using a rather conservative approach, a protein-protein interaction network within our preeclampsia meta-signature emerges with CREBBP as key protein within this network. CREBBP interacts with several proteins well known to be associated with preeclampsia, such as NFE2L2 [39, 40], LEP [41] and VEGF/FLT1 [42], but also with proteins not-yet related to preeclampsia. CREBBP and EP300 are transcriptional/epigenetic regulators involved in many physiological and pathological processes [43]. Although their functions appear to be largely redundant, differences have been reported [34]. Even though *CREBBP*/*EP300* were not identified as DEGs in the other two published meta-analyses, their involvement in preeclampsia was suggested based on association with differentially expressed transcription factors by Vaiman and colleagues [6].

Mutations in *CREBBP* and *EP300* are causative for Rubinstein-Taybi syndrome (RSTS, OMIM 180849 and 613684). RSTS is a rare (approximately 1 in 100,000 newborns) autosomal dominant congenital disorder characterized by mental retardation, dysmorphic facial features, postnatal growth deficiency, and skeletal abnormalities [44]. Almost all mutations occur de novo and range from large deletions to point mutations. There is no clear genotype-phenotype correlation, but loss of the CREBBP/EP300 histone acetyltransferase activity appears to be essential for the development of RSTS [45]. Interestingly, there have been frequent reports of preeclampsia-complicated pregnancies in mothers giving birth to a RSTS affected child [46–51]. Thus, the fetal genotype not only causes RSTS in the neonate but also influences placenta function resulting in an increased incidence of preeclampsia in the mother. If we translate these observations and our meta-analysis results to non-RSTS women, it appears that decreased CEBBP/EP300 levels are detrimental for placenta functioning, resulting in an increased probability for the mother to develop preeclampsia.

Analysis of canonical pathways and gene expression patterns clearly indicated a prominent role for hypoxia in the establishment of the preeclamptic gene meta-signature, a conclusion that was also drawn by Moslehi et al, based on the DEGs from their meta-analysis [5].

**Fig 5:**
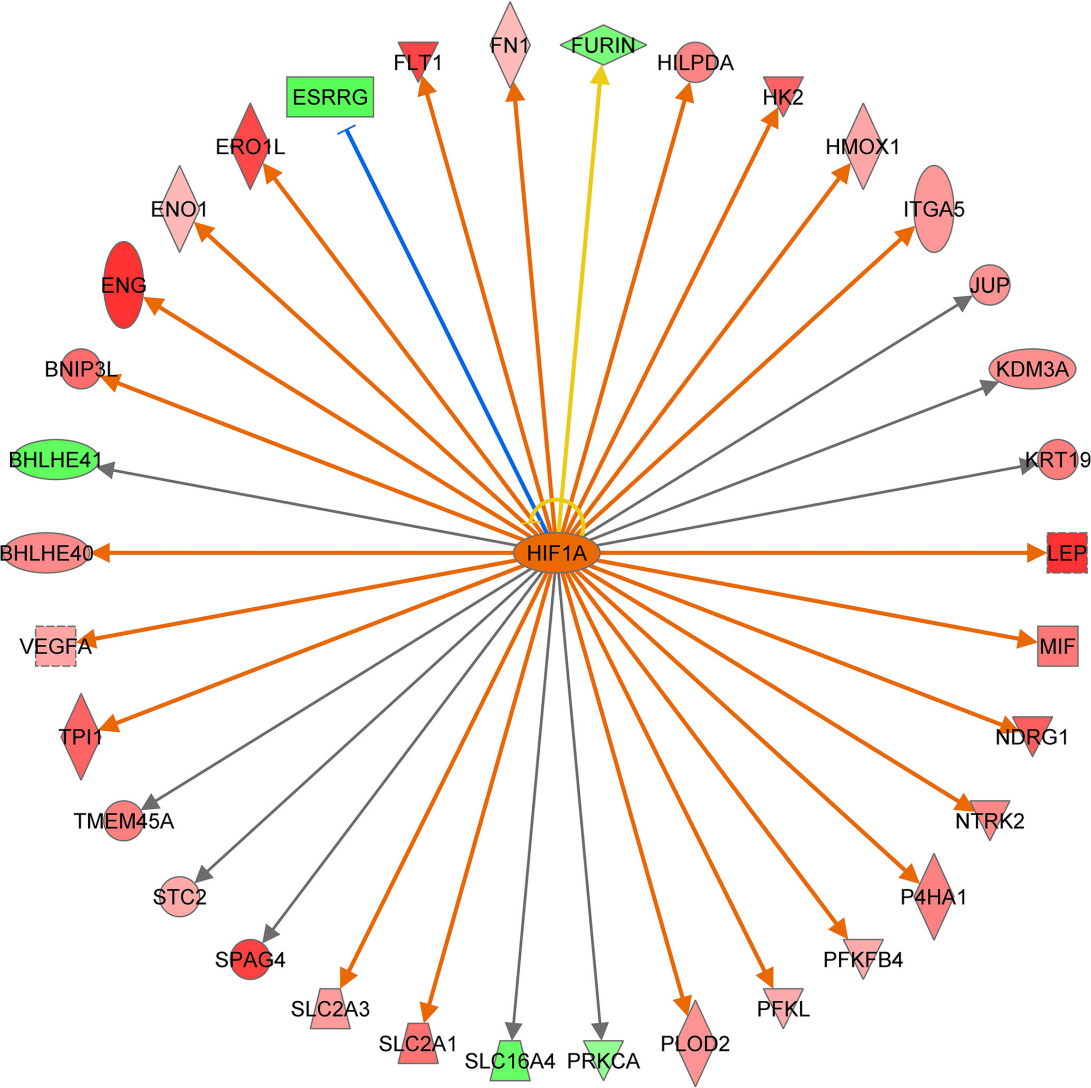
Ingenuity Pathway Analysis identifies HIF1A as the most prominently activated upstream regulator. Thirty four out of 388 genes are direct HIF1A downstream targets according to the Ingenuity Knowledge Database. Arrows indicate the predicted relationship: orange = leads to activation (23 genes), blue = leads to inhibition (1 gene), grey = effect not predicted (9 genes), yellow = findings inconsistent with the state of downstream molecule (1 gene). Red symbols indicate genes upregulated in preeclamptic placenta, green symbols indicate genes downregulated in preeclamptic placenta. Intensity of the coloring is related to the level of up/downregulation.

Preeclamptic placentas are compromised by hypoxia/ischemia, and the effect on gene expression is largely mediated via HIF-1α [52]. More than 10% of the upregulated meta-signature genes are direct targets of HIF-1α (Fig 5). CREBBP/EP300 is an important transcriptional coactivator of HIF-1α [53–56]. As a result, decreased CREBBP/EP300 levels may impair the placenta’s capacity to respond to low oxygen, resulting in a further decline of its (preeclamptic) condition.

## Conclusions

The robust 388-gene signature of differentially expressed genes in the preeclamptic placenta serves as a starting point to investigate their function in the development of preeclampsia. This will result in a better understanding of the molecular basis of this disease and opens up the opportunity to develop rational therapies targeting the placental dysfunction causal to preeclampsia.

## Supporting Information

S1 Fig Forest plots of meta-signature genes. Forest plots of the 388 differentially expressed genes in the preeclamptic placenta ordered on the absolute value of the standardized mean difference (see S1 Table). Squares are proportional to weights used in the meta-analysis, and the lines represent the 95% confidence interval. The diamond represents the standardized mean difference (log2 scale).
(PDF)

S2 Fig STRING protein interaction analysis. All 388 genes from the meta‐signature were used as input for STRING analysis and a network was built based on high confidence (0.8) evidence from experimental protein‐protein interaction (blue lines) and curated (purple lines) databases. Proteins are indicated by nodes labeled with the encoding gene symbol. Two genes (CEBPA and SEPP1) are not present in the used STRING version 9.1. The network is enriched in interactions (p = 0.007) using the intersection of 8,612 genes present on all platforms as background. Additional cluster analysis was used to color the nodes of the interaction networks. Yellow nodes represent proteins that have no protein‐protein interaction at this confidence setting.
(PDF)

S1 Table 388-gene preeclampsia meta-signature. (XLSX)

S2 Table Comparison of current 388-gene meta-signature with previously generated 40-gene meta-signature. (XLSX)

S3 Table Pathway analysis (all significant pathways). (XLSX)

S4 Table Expression of meta-signature genes in placentas of different gestational ages. Indicated microarray samples were analyzed by the Gene Expression Barcode 3.0 and the resulting z-scores are shown. The z-score is the number of standard deviations above the mean expression signal of the unexpressed null distribution. An average z-score of ≥ 3 for a gene-specific probe has been labeled as being expressed in that trimester.
(XLSX)

S1 Text Supplementary Methods. (DOCX)
