# Supplementary material for: Meta-Analysis of Placental Transcriptome Data Identifies a Novel Molecular Pathway Related to Preeclampsia
Source: PLoS One. 2015 Jul 14;10(7):e0132468. doi: 10.1371/journal.pone.0132468 (PMC4501668; doi:10.1371/journal.pone.0132468)
Supplement: S1 Fig — Forest plots of the 388 differentially expressed genes in the preeclamptic placenta ordered on the absolute value of the standardized mean difference (see S1 Table). Squares are proportional to weights used in the meta-analysis, and the lines represent the 95% confidence interval. The diamond represents the standardized mean difference (log2 scale). (PDF) [file pone.0132468.s001.pdf]

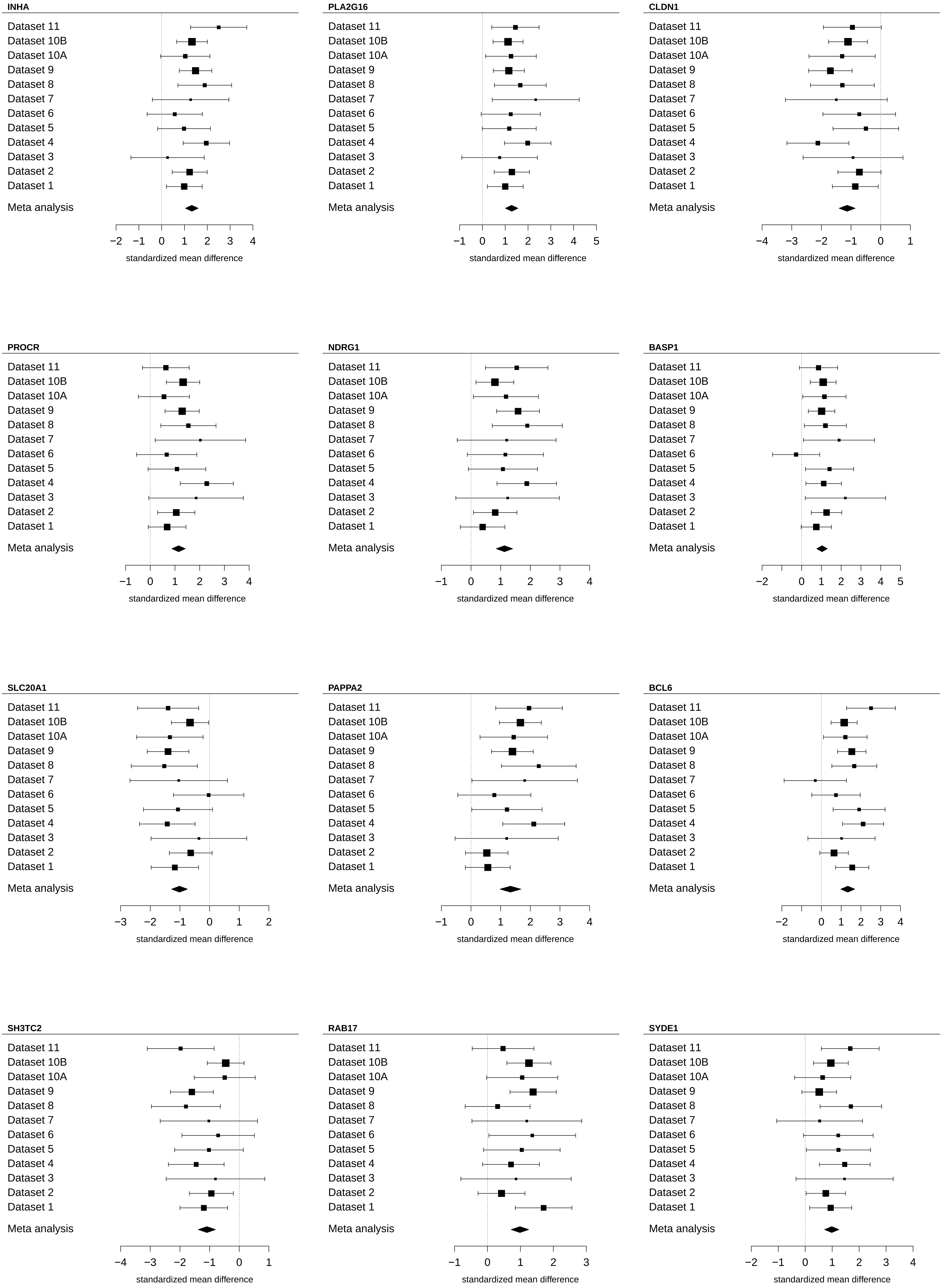
INHA
PLA2G16
CLDN1
PROCR
NDRG1
BASP1
SLC20A1
PAPPA2
BCL6
SH3TC2
RAB17
SYDE1
Dataset 11
Dataset 10B
Dataset 10A
Dataset 9
Dataset 8
Dataset 7
Dataset 6
Dataset 5
Dataset 4
Dataset 3
Dataset 2
Dataset 1
Meta analysis
standardized mean difference

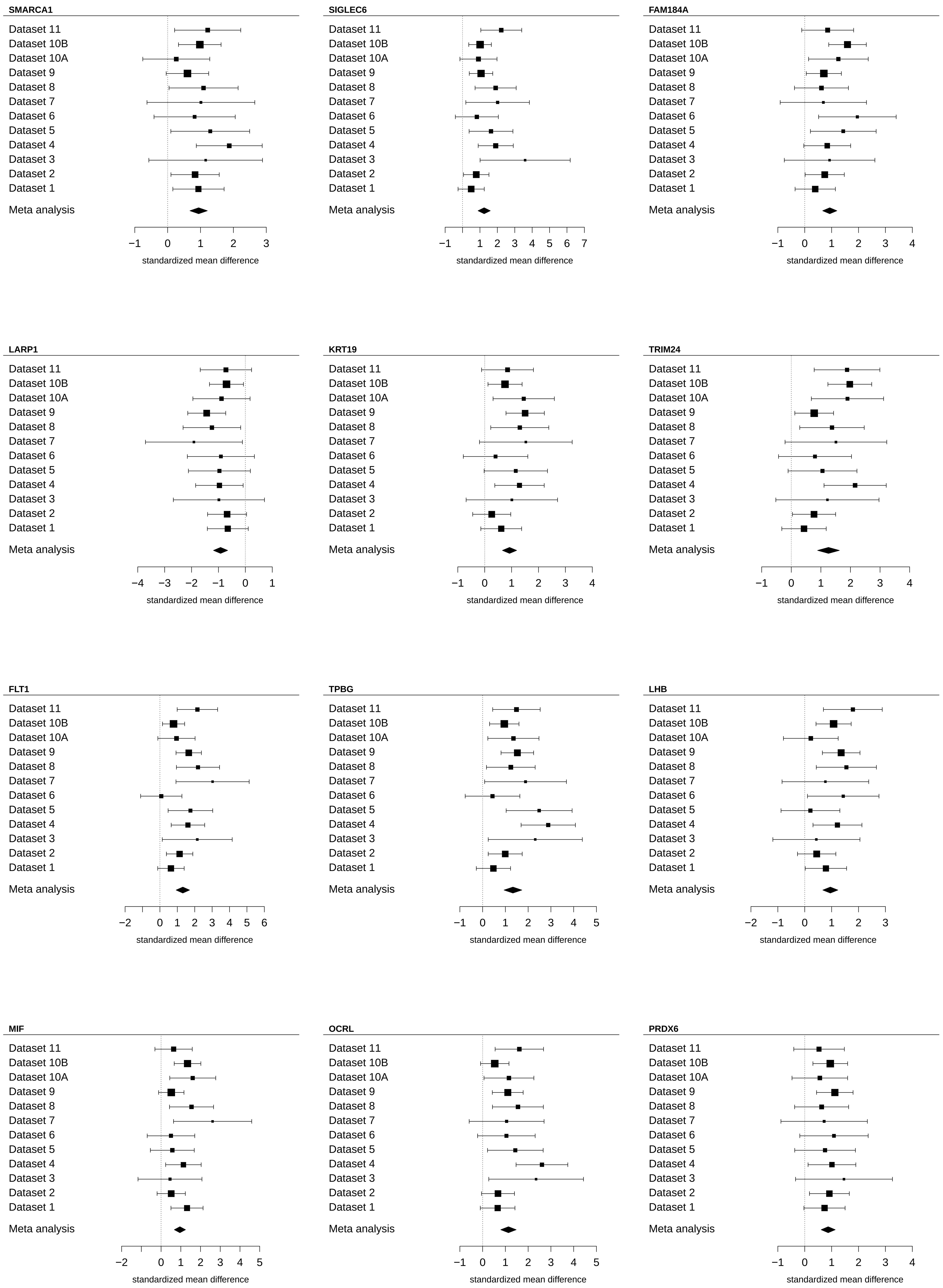

SMARCA1
SIGLEC6
FAM184A
LARP1
KRT19
TRIM24
FLT1
TPBG
LHB
MIF
OCRL
PRDX6
Dataset 11
Dataset 10B
Dataset 10A
Dataset 9
Dataset 8
Dataset 7
Dataset 6
Dataset 5
Dataset 4
Dataset 3
Dataset 2
Dataset 1
Meta analysis
standardized mean difference

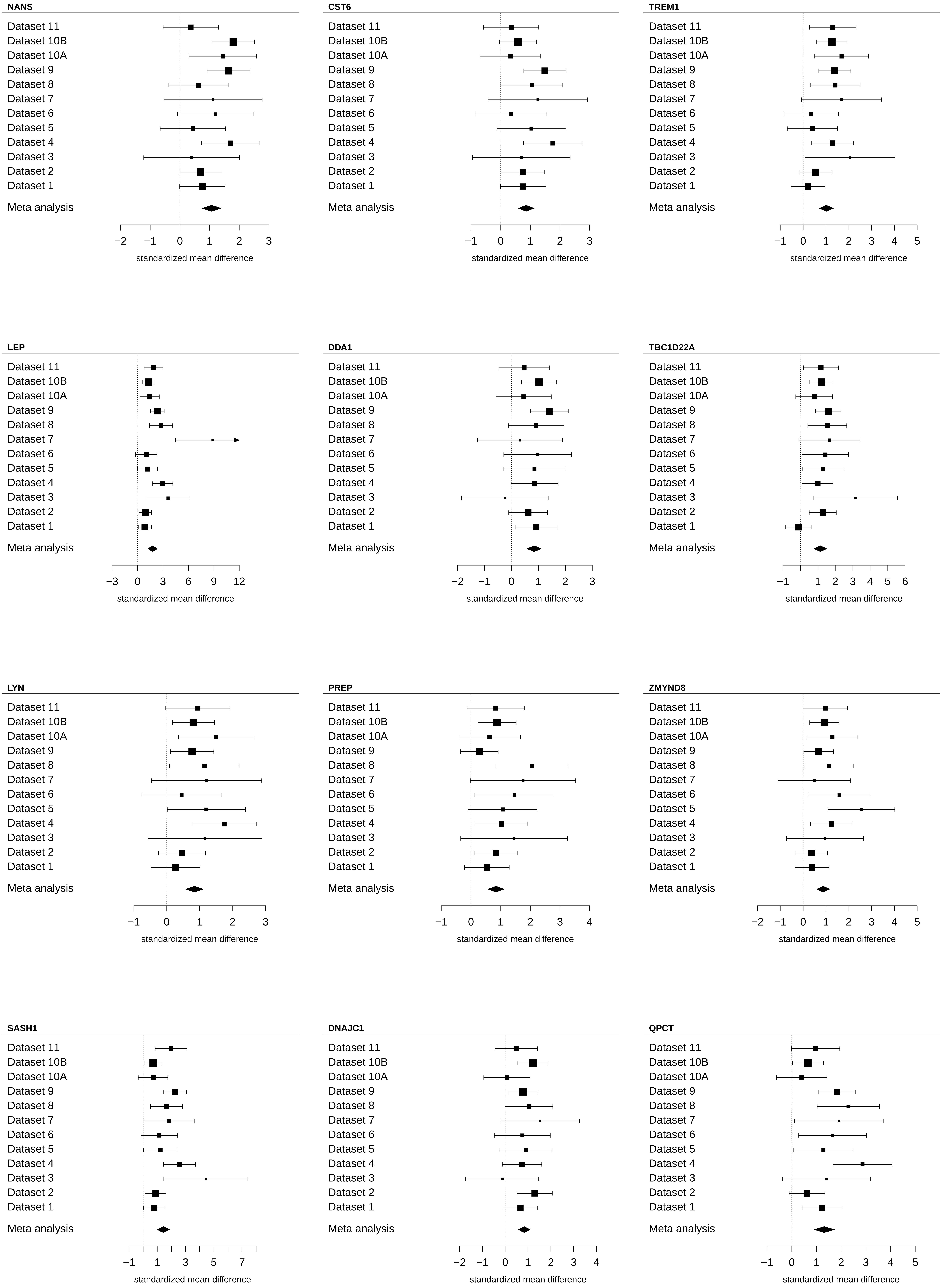
NANS
Dataset 11
Dataset 10B
Dataset 10A
Dataset 9
Dataset 8
Dataset 7
Dataset 6
Dataset 5
Dataset 4
Dataset 3
Dataset 2
Dataset 1
Meta analysis
−2 −1 0 1 2 3
standardized mean difference
CST6
Dataset 11
Dataset 10B
Dataset 10A
Dataset 9
Dataset 8
Dataset 7
Dataset 6
Dataset 5
Dataset 4
Dataset 3
Dataset 2
Dataset 1
Meta analysis
−1 0 1 2 3
standardized mean difference
TREM1
Dataset 11
Dataset 10B
Dataset 10A
Dataset 9
Dataset 8
Dataset 7
Dataset 6
Dataset 5
Dataset 4
Dataset 3
Dataset 2
Dataset 1
Meta analysis
−1 0 1 2 3 4 5
standardized mean difference
LEP
Dataset 11
Dataset 10B
Dataset 10A
Dataset 9
Dataset 8
Dataset 7
Dataset 6
Dataset 5
Dataset 4
Dataset 3
Dataset 2
Dataset 1
Meta analysis
−3 0 3 6 9 12
standardized mean difference
DDA1
Dataset 11
Dataset 10B
Dataset 10A
Dataset 9
Dataset 8
Dataset 7
Dataset 6
Dataset 5
Dataset 4
Dataset 3
Dataset 2
Dataset 1
Meta analysis
−2 −1 0 1 2 3
standardized mean difference
TBC1D22A
Dataset 11
Dataset 10B
Dataset 10A
Dataset 9
Dataset 8
Dataset 7
Dataset 6
Dataset 5
Dataset 4
Dataset 3
Dataset 2
Dataset 1
Meta analysis
−1 1 2 3 4 5 6
standardized mean difference
LYN
Dataset 11
Dataset 10B
Dataset 10A
Dataset 9
Dataset 8
Dataset 7
Dataset 6
Dataset 5
Dataset 4
Dataset 3
Dataset 2
Dataset 1
Meta analysis
−1 0 1 2 3
standardized mean difference
PREP
Dataset 11
Dataset 10B
Dataset 10A
Dataset 9
Dataset 8
Dataset 7
Dataset 6
Dataset 5
Dataset 4
Dataset 3
Dataset 2
Dataset 1
Meta analysis
−1 0 1 2 3 4
standardized mean difference
ZMYND8
Dataset 11
Dataset 10B
Dataset 10A
Dataset 9
Dataset 8
Dataset 7
Dataset 6
Dataset 5
Dataset 4
Dataset 3
Dataset 2
Dataset 1
Meta analysis
−2 −1 0 1 2 3 4 5
standardized mean difference
SASH1
Dataset 11
Dataset 10B
Dataset 10A
Dataset 9
Dataset 8
Dataset 7
Dataset 6
Dataset 5
Dataset 4
Dataset 3
Dataset 2
Dataset 1
Meta analysis
−1 1 3 5 7
standardized mean difference
DNAJC1
Dataset 11
Dataset 10B
Dataset 10A
Dataset 9
Dataset 8
Dataset 7
Dataset 6
Dataset 5
Dataset 4
Dataset 3
Dataset 2
Dataset 1
Meta analysis
−2 −1 0 1 2 3 4
standardized mean difference
QPCT
Dataset 11
Dataset 10B
Dataset 10A
Dataset 9
Dataset 8
Dataset 7
Dataset 6
Dataset 5
Dataset 4
Dataset 3
Dataset 2
Dataset 1
Meta analysis
−1 0 1 2 3 4 5
standardized mean difference

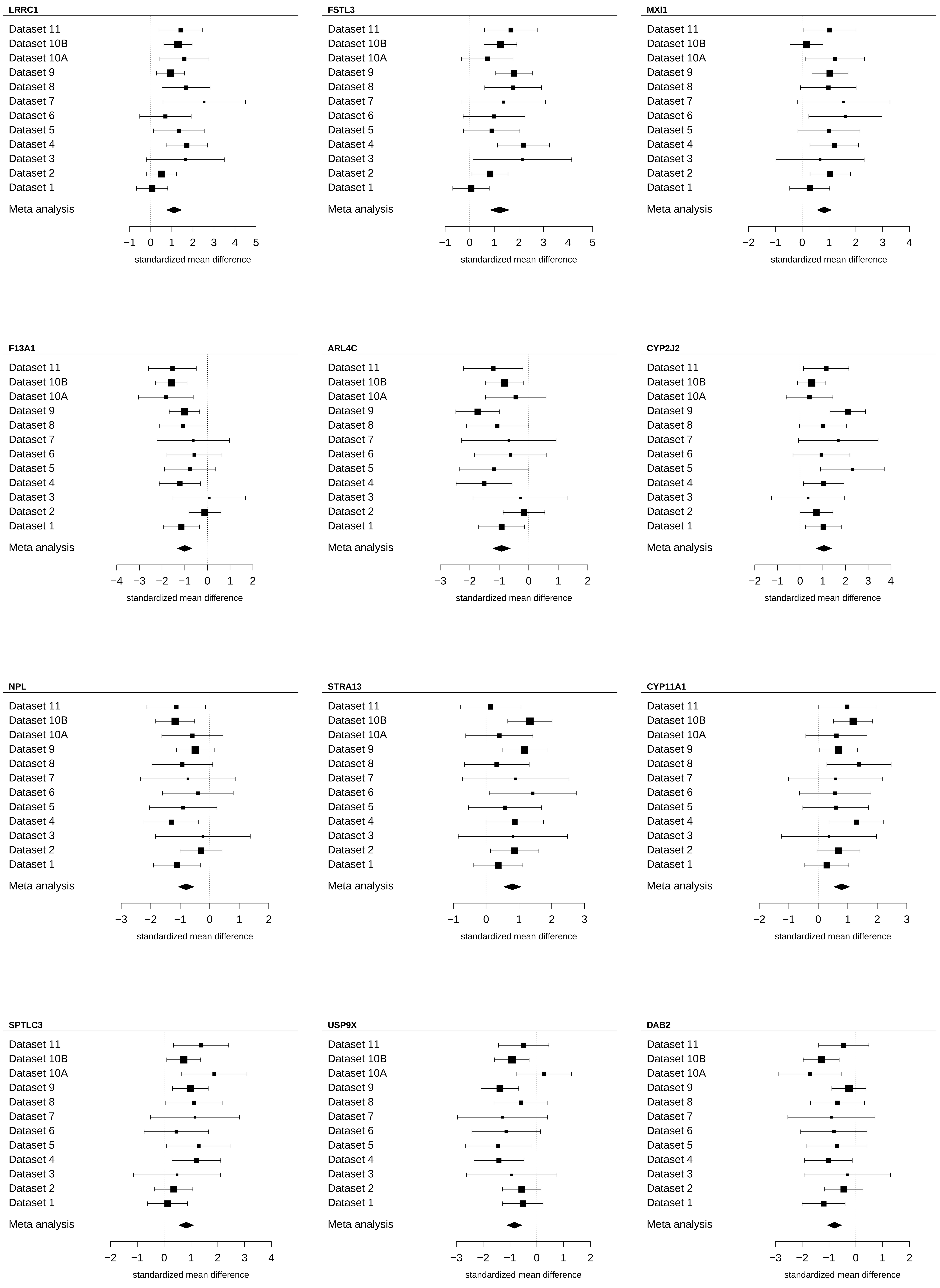
LRRC1
FSTL3
MXI1
F13A1
ARL4C
CYP2J2
NPL
STRA13
CYP11A1
SPTLC3
USP9X
DAB2
Dataset 11
Dataset 10B
Dataset 10A
Dataset 9
Dataset 8
Dataset 7
Dataset 6
Dataset 5
Dataset 4
Dataset 3
Dataset 2
Dataset 1
Meta analysis
standardized mean difference

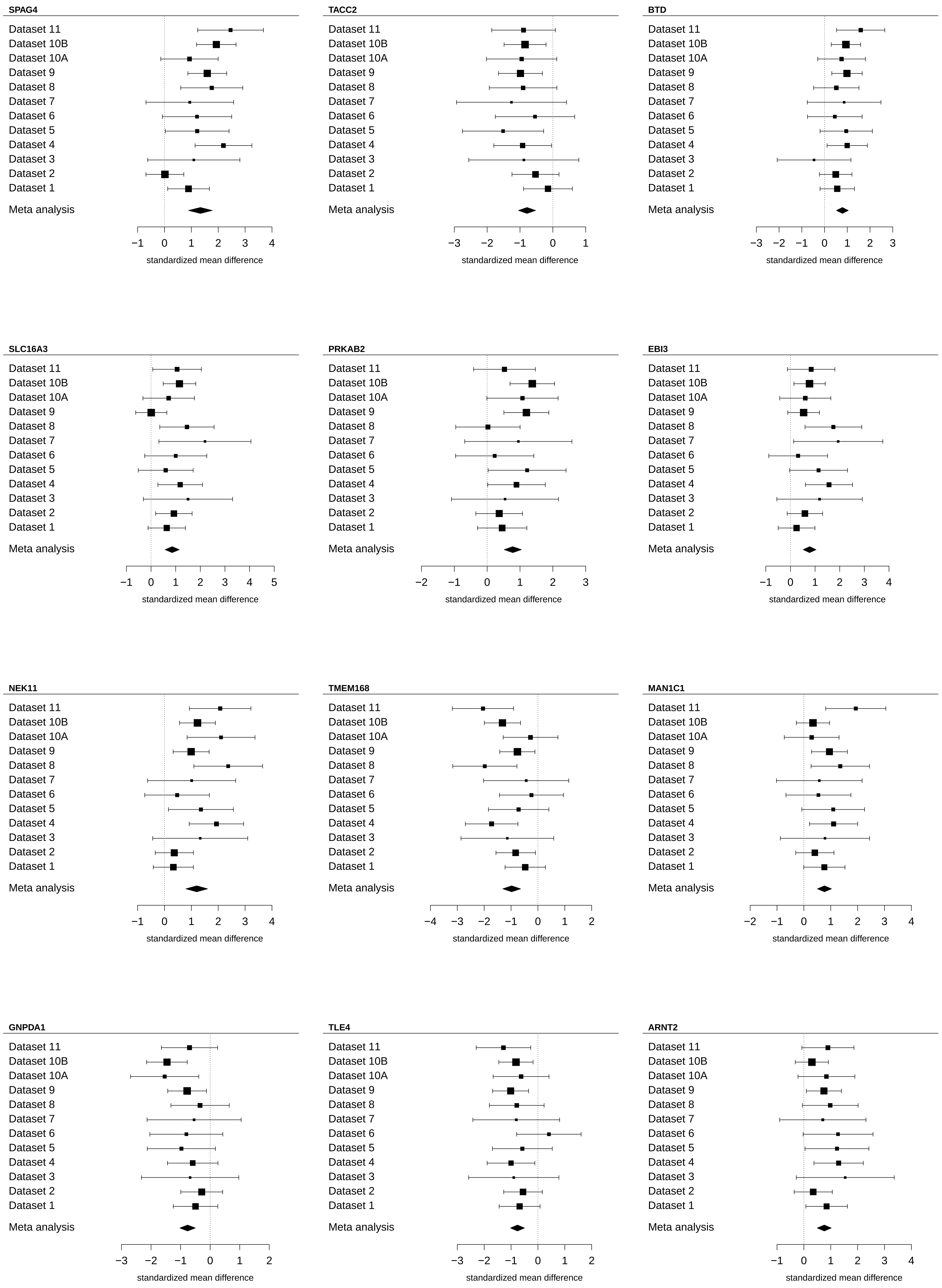
SPAG4
Dataset 11
Dataset 10B
Dataset 10A
Dataset 9
Dataset 8
Dataset 7
Dataset 6
Dataset 5
Dataset 4
Dataset 3
Dataset 2
Dataset 1
Meta analysis
−1 0 1 2 3 4
standardized mean difference
TACC2
Dataset 11
Dataset 10B
Dataset 10A
Dataset 9
Dataset 8
Dataset 7
Dataset 6
Dataset 5
Dataset 4
Dataset 3
Dataset 2
Dataset 1
Meta analysis
−3 −2 −1 0 1
standardized mean difference
BTD
Dataset 11
Dataset 10B
Dataset 10A
Dataset 9
Dataset 8
Dataset 7
Dataset 6
Dataset 5
Dataset 4
Dataset 3
Dataset 2
Dataset 1
Meta analysis
−3 −2 −1 0 1 2 3
standardized mean difference
SLC16A3
Dataset 11
Dataset 10B
Dataset 10A
Dataset 9
Dataset 8
Dataset 7
Dataset 6
Dataset 5
Dataset 4
Dataset 3
Dataset 2
Dataset 1
Meta analysis
−1 0 1 2 3 4 5
standardized mean difference
PRKAB2
Dataset 11
Dataset 10B
Dataset 10A
Dataset 9
Dataset 8
Dataset 7
Dataset 6
Dataset 5
Dataset 4
Dataset 3
Dataset 2
Dataset 1
Meta analysis
−2 −1 0 1 2 3
standardized mean difference
EBI3
Dataset 11
Dataset 10B
Dataset 10A
Dataset 9
Dataset 8
Dataset 7
Dataset 6
Dataset 5
Dataset 4
Dataset 3
Dataset 2
Dataset 1
Meta analysis
−1 0 1 2 3 4
standardized mean difference
NEK11
Dataset 11
Dataset 10B
Dataset 10A
Dataset 9
Dataset 8
Dataset 7
Dataset 6
Dataset 5
Dataset 4
Dataset 3
Dataset 2
Dataset 1
Meta analysis
−1 0 1 2 3 4
standardized mean difference
TMEM168
Dataset 11
Dataset 10B
Dataset 10A
Dataset 9
Dataset 8
Dataset 7
Dataset 6
Dataset 5
Dataset 4
Dataset 3
Dataset 2
Dataset 1
Meta analysis
−4 −3 −2 −1 0 1 2
standardized mean difference
MAN1C1
Dataset 11
Dataset 10B
Dataset 10A
Dataset 9
Dataset 8
Dataset 7
Dataset 6
Dataset 5
Dataset 4
Dataset 3
Dataset 2
Dataset 1
Meta analysis
−2 −1 0 1 2 3 4
standardized mean difference
GNPDA1
Dataset 11
Dataset 10B
Dataset 10A
Dataset 9
Dataset 8
Dataset 7
Dataset 6
Dataset 5
Dataset 4
Dataset 3
Dataset 2
Dataset 1
Meta analysis
−3 −2 −1 0 1 2
standardized mean difference
TLE4
Dataset 11
Dataset 10B
Dataset 10A
Dataset 9
Dataset 8
Dataset 7
Dataset 6
Dataset 5
Dataset 4
Dataset 3
Dataset 2
Dataset 1
Meta analysis
−3 −2 −1 0 1 2
standardized mean difference
ARNT2
Dataset 11
Dataset 10B
Dataset 10A
Dataset 9
Dataset 8
Dataset 7
Dataset 6
Dataset 5
Dataset 4
Dataset 3
Dataset 2
Dataset 1
Meta analysis
−1 0 1 2 3 4
standardized mean difference

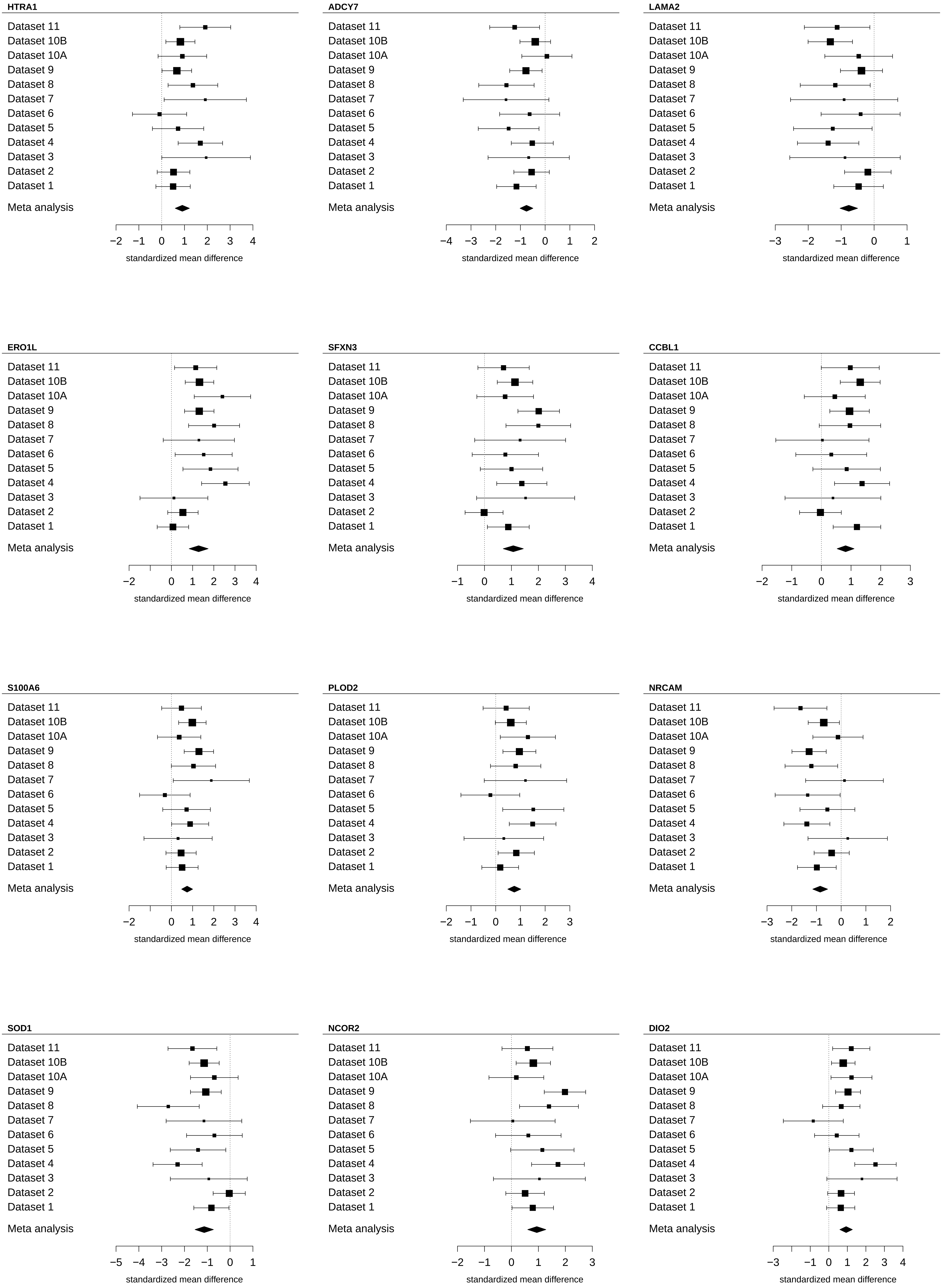
HTRA1
Dataset 11
Dataset 10B
Dataset 10A
Dataset 9
Dataset 8
Dataset 7
Dataset 6
Dataset 5
Dataset 4
Dataset 3
Dataset 2
Dataset 1
Meta analysis
−2 −1 0 1 2 3 4
standardized mean difference
ADCY7
Dataset 11
Dataset 10B
Dataset 10A
Dataset 9
Dataset 8
Dataset 7
Dataset 6
Dataset 5
Dataset 4
Dataset 3
Dataset 2
Dataset 1
Meta analysis
−4 −3 −2 −1 0 1 2
standardized mean difference
LAMA2
Dataset 11
Dataset 10B
Dataset 10A
Dataset 9
Dataset 8
Dataset 7
Dataset 6
Dataset 5
Dataset 4
Dataset 3
Dataset 2
Dataset 1
Meta analysis
−3 −2 −1 0 1
standardized mean difference
ERO1L
Dataset 11
Dataset 10B
Dataset 10A
Dataset 9
Dataset 8
Dataset 7
Dataset 6
Dataset 5
Dataset 4
Dataset 3
Dataset 2
Dataset 1
Meta analysis
−2 0 1 2 3 4
standardized mean difference
SFXN3
Dataset 11
Dataset 10B
Dataset 10A
Dataset 9
Dataset 8
Dataset 7
Dataset 6
Dataset 5
Dataset 4
Dataset 3
Dataset 2
Dataset 1
Meta analysis
−1 0 1 2 3 4
standardized mean difference
CCBL1
Dataset 11
Dataset 10B
Dataset 10A
Dataset 9
Dataset 8
Dataset 7
Dataset 6
Dataset 5
Dataset 4
Dataset 3
Dataset 2
Dataset 1
Meta analysis
−2 −1 0 1 2 3
standardized mean difference
S100A6
Dataset 11
Dataset 10B
Dataset 10A
Dataset 9
Dataset 8
Dataset 7
Dataset 6
Dataset 5
Dataset 4
Dataset 3
Dataset 2
Dataset 1
Meta analysis
−2 0 1 2 3 4
standardized mean difference
PLOD2
Dataset 11
Dataset 10B
Dataset 10A
Dataset 9
Dataset 8
Dataset 7
Dataset 6
Dataset 5
Dataset 4
Dataset 3
Dataset 2
Dataset 1
Meta analysis
−2 −1 0 1 2 3
standardized mean difference
NRCAM
Dataset 11
Dataset 10B
Dataset 10A
Dataset 9
Dataset 8
Dataset 7
Dataset 6
Dataset 5
Dataset 4
Dataset 3
Dataset 2
Dataset 1
Meta analysis
−3 −2 −1 0 1 2
standardized mean difference
SOD1
Dataset 11
Dataset 10B
Dataset 10A
Dataset 9
Dataset 8
Dataset 7
Dataset 6
Dataset 5
Dataset 4
Dataset 3
Dataset 2
Dataset 1
Meta analysis
−5 −4 −3 −2 −1 0 1
standardized mean difference
NCOR2
Dataset 11
Dataset 10B
Dataset 10A
Dataset 9
Dataset 8
Dataset 7
Dataset 6
Dataset 5
Dataset 4
Dataset 3
Dataset 2
Dataset 1
Meta analysis
−2 −1 0 1 2 3
standardized mean difference
DIO2
Dataset 11
Dataset 10B
Dataset 10A
Dataset 9
Dataset 8
Dataset 7
Dataset 6
Dataset 5
Dataset 4
Dataset 3
Dataset 2
Dataset 1
Meta analysis
−3 −1 0 1 2 3 4
standardized mean difference

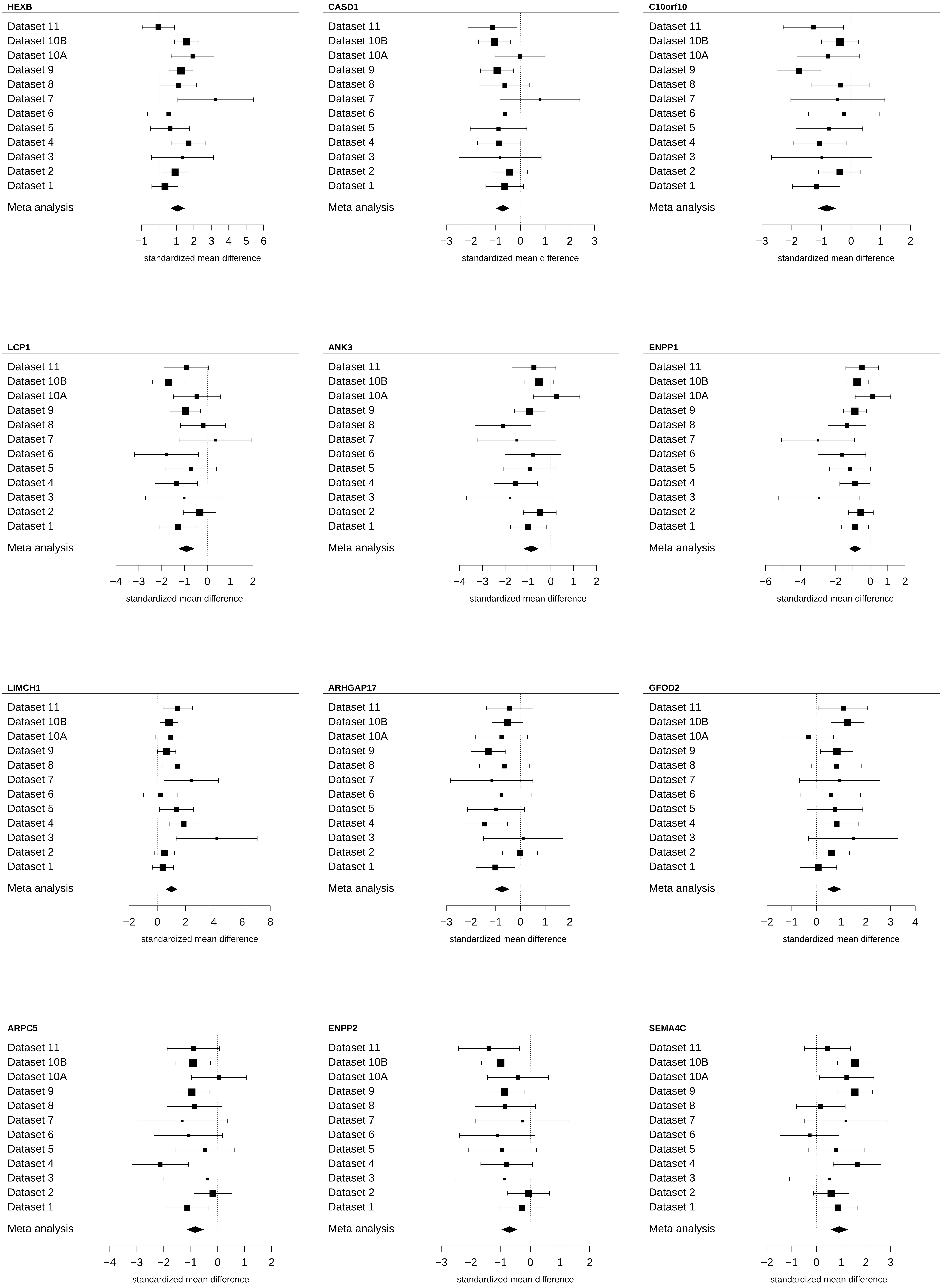
HEXB
Dataset 11
Dataset 10B
Dataset 10A
Dataset 9
Dataset 8
Dataset 7
Dataset 6
Dataset 5
Dataset 4
Dataset 3
Dataset 2
Dataset 1
Meta analysis
−1 1 2 3 4 5 6
standardized mean difference
CASD1
Dataset 11
Dataset 10B
Dataset 10A
Dataset 9
Dataset 8
Dataset 7
Dataset 6
Dataset 5
Dataset 4
Dataset 3
Dataset 2
Dataset 1
Meta analysis
−3 −2 −1 0 1 2 3
standardized mean difference
C10orf10
Dataset 11
Dataset 10B
Dataset 10A
Dataset 9
Dataset 8
Dataset 7
Dataset 6
Dataset 5
Dataset 4
Dataset 3
Dataset 2
Dataset 1
Meta analysis
−3 −2 −1 0 1 2
standardized mean difference
LCP1
Dataset 11
Dataset 10B
Dataset 10A
Dataset 9
Dataset 8
Dataset 7
Dataset 6
Dataset 5
Dataset 4
Dataset 3
Dataset 2
Dataset 1
Meta analysis
−4 −3 −2 −1 0 1 2
standardized mean difference
ANK3
Dataset 11
Dataset 10B
Dataset 10A
Dataset 9
Dataset 8
Dataset 7
Dataset 6
Dataset 5
Dataset 4
Dataset 3
Dataset 2
Dataset 1
Meta analysis
−4 −3 −2 −1 0 1 2
standardized mean difference
ENPP1
Dataset 11
Dataset 10B
Dataset 10A
Dataset 9
Dataset 8
Dataset 7
Dataset 6
Dataset 5
Dataset 4
Dataset 3
Dataset 2
Dataset 1
Meta analysis
−6 −4 −2 0 1 2
standardized mean difference
LIMCH1
Dataset 11
Dataset 10B
Dataset 10A
Dataset 9
Dataset 8
Dataset 7
Dataset 6
Dataset 5
Dataset 4
Dataset 3
Dataset 2
Dataset 1
Meta analysis
−2 0 2 4 6 8
standardized mean difference
ARHGAP17
Dataset 11
Dataset 10B
Dataset 10A
Dataset 9
Dataset 8
Dataset 7
Dataset 6
Dataset 5
Dataset 4
Dataset 3
Dataset 2
Dataset 1
Meta analysis
−3 −2 −1 0 1 2
standardized mean difference
GFOD2
Dataset 11
Dataset 10B
Dataset 10A
Dataset 9
Dataset 8
Dataset 7
Dataset 6
Dataset 5
Dataset 4
Dataset 3
Dataset 2
Dataset 1
Meta analysis
−2 −1 0 1 2 3 4
standardized mean difference
ARPC5
Dataset 11
Dataset 10B
Dataset 10A
Dataset 9
Dataset 8
Dataset 7
Dataset 6
Dataset 5
Dataset 4
Dataset 3
Dataset 2
Dataset 1
Meta analysis
−4 −3 −2 −1 0 1 2
standardized mean difference
ENPP2
Dataset 11
Dataset 10B
Dataset 10A
Dataset 9
Dataset 8
Dataset 7
Dataset 6
Dataset 5
Dataset 4
Dataset 3
Dataset 2
Dataset 1
Meta analysis
−3 −2 −1 0 1 2
standardized mean difference
SEMA4C
Dataset 11
Dataset 10B
Dataset 10A
Dataset 9
Dataset 8
Dataset 7
Dataset 6
Dataset 5
Dataset 4
Dataset 3
Dataset 2
Dataset 1
Meta analysis
−2 −1 0 1 2 3
standardized mean difference

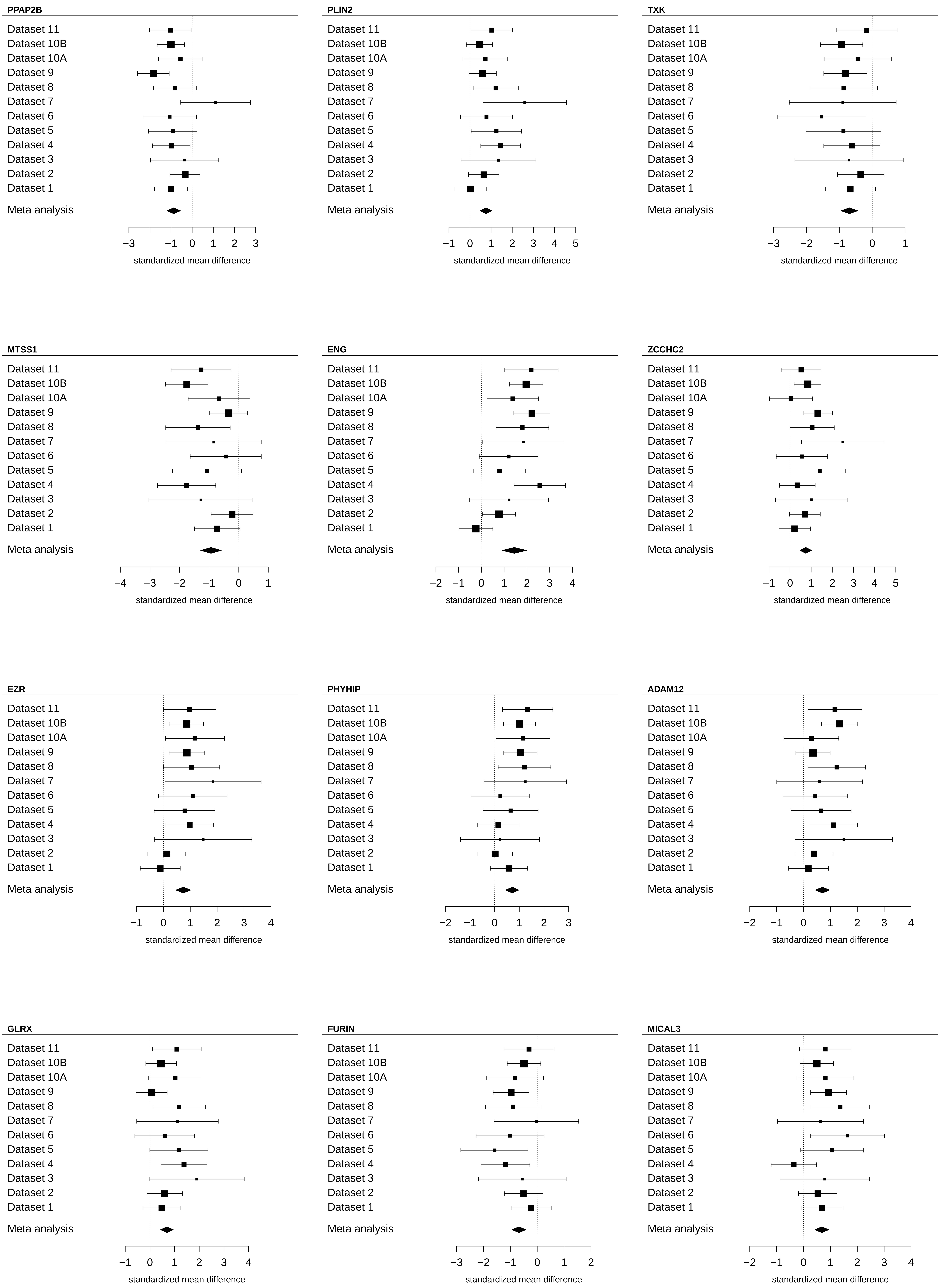
PPAP2B
Dataset 11
Dataset 10B
Dataset 10A
Dataset 9
Dataset 8
Dataset 7
Dataset 6
Dataset 5
Dataset 4
Dataset 3
Dataset 2
Dataset 1
Meta analysis
−3 −1 0 1 2 3
standardized mean difference
PLIN2
Dataset 11
Dataset 10B
Dataset 10A
Dataset 9
Dataset 8
Dataset 7
Dataset 6
Dataset 5
Dataset 4
Dataset 3
Dataset 2
Dataset 1
Meta analysis
−1 0 1 2 3 4 5
standardized mean difference
TXK
Dataset 11
Dataset 10B
Dataset 10A
Dataset 9
Dataset 8
Dataset 7
Dataset 6
Dataset 5
Dataset 4
Dataset 3
Dataset 2
Dataset 1
Meta analysis
−3 −2 −1 0 1
standardized mean difference
MTSS1
Dataset 11
Dataset 10B
Dataset 10A
Dataset 9
Dataset 8
Dataset 7
Dataset 6
Dataset 5
Dataset 4
Dataset 3
Dataset 2
Dataset 1
Meta analysis
−4 −3 −2 −1 0 1
standardized mean difference
ENG
Dataset 11
Dataset 10B
Dataset 10A
Dataset 9
Dataset 8
Dataset 7
Dataset 6
Dataset 5
Dataset 4
Dataset 3
Dataset 2
Dataset 1
Meta analysis
−2 −1 0 1 2 3 4
standardized mean difference
ZCCHC2
Dataset 11
Dataset 10B
Dataset 10A
Dataset 9
Dataset 8
Dataset 7
Dataset 6
Dataset 5
Dataset 4
Dataset 3
Dataset 2
Dataset 1
Meta analysis
−1 0 1 2 3 4 5
standardized mean difference
EZR
Dataset 11
Dataset 10B
Dataset 10A
Dataset 9
Dataset 8
Dataset 7
Dataset 6
Dataset 5
Dataset 4
Dataset 3
Dataset 2
Dataset 1
Meta analysis
−1 0 1 2 3 4
standardized mean difference
PHYHIP
Dataset 11
Dataset 10B
Dataset 10A
Dataset 9
Dataset 8
Dataset 7
Dataset 6
Dataset 5
Dataset 4
Dataset 3
Dataset 2
Dataset 1
Meta analysis
−2 −1 0 1 2 3
standardized mean difference
ADAM12
Dataset 11
Dataset 10B
Dataset 10A
Dataset 9
Dataset 8
Dataset 7
Dataset 6
Dataset 5
Dataset 4
Dataset 3
Dataset 2
Dataset 1
Meta analysis
−2 −1 0 1 2 3 4
standardized mean difference
GLRX
Dataset 11
Dataset 10B
Dataset 10A
Dataset 9
Dataset 8
Dataset 7
Dataset 6
Dataset 5
Dataset 4
Dataset 3
Dataset 2
Dataset 1
Meta analysis
−1 0 1 2 3 4
standardized mean difference
FURIN
Dataset 11
Dataset 10B
Dataset 10A
Dataset 9
Dataset 8
Dataset 7
Dataset 6
Dataset 5
Dataset 4
Dataset 3
Dataset 2
Dataset 1
Meta analysis
−3 −2 −1 0 1 2
standardized mean difference
MICAL3
Dataset 11
Dataset 10B
Dataset 10A
Dataset 9
Dataset 8
Dataset 7
Dataset 6
Dataset 5
Dataset 4
Dataset 3
Dataset 2
Dataset 1
Meta analysis
−2 −1 0 1 2 3 4
standardized mean difference

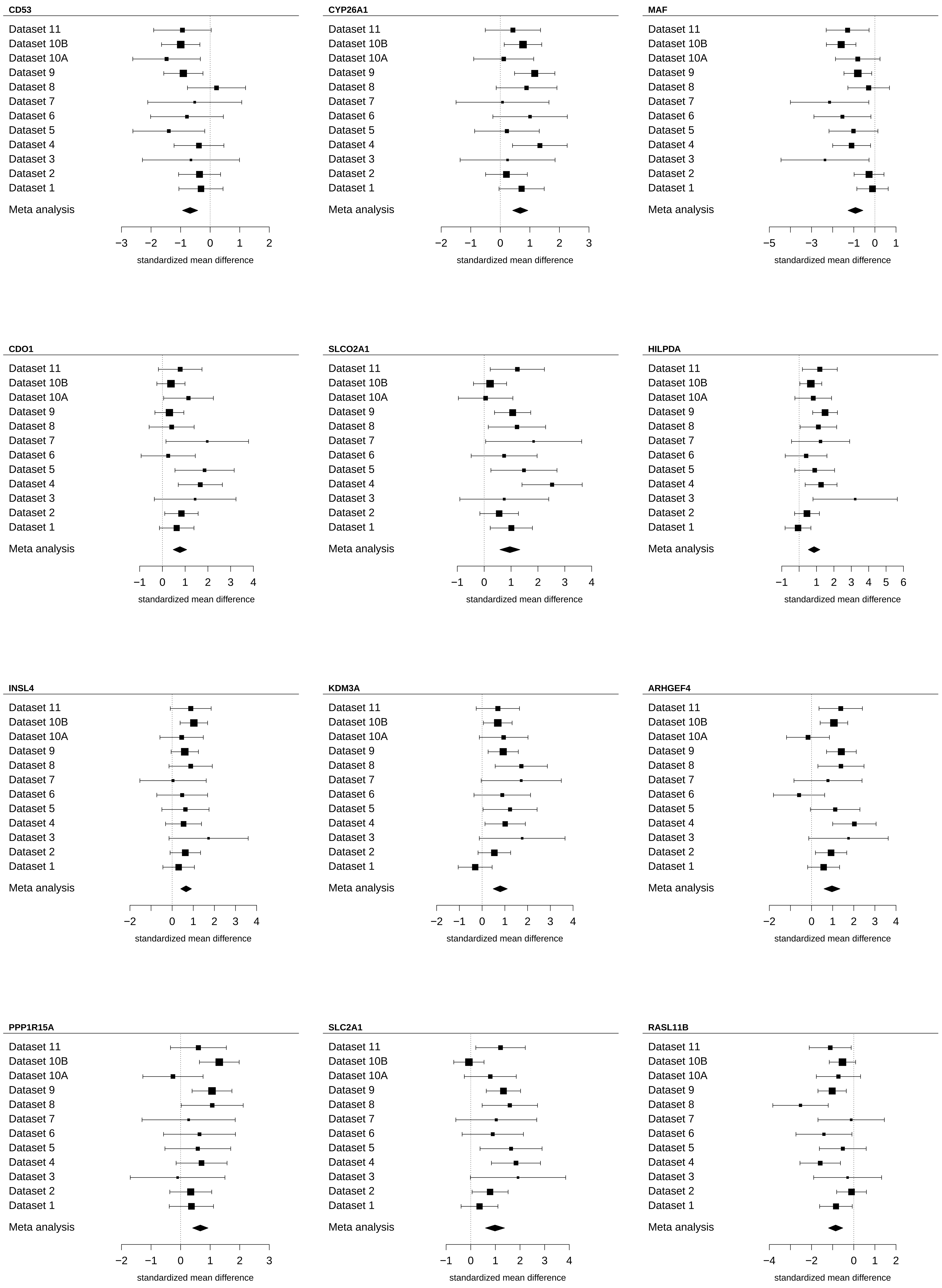
CD53
CYP26A1
MAF
CDO1
SLCO2A1
HILPDA
INSL4
KDM3A
ARHGEF4
PPP1R15A
SLC2A1
RASL11B
Dataset 11
Dataset 10B
Dataset 10A
Dataset 9
Dataset 8
Dataset 7
Dataset 6
Dataset 5
Dataset 4
Dataset 3
Dataset 2
Dataset 1
Meta analysis
standardized mean difference

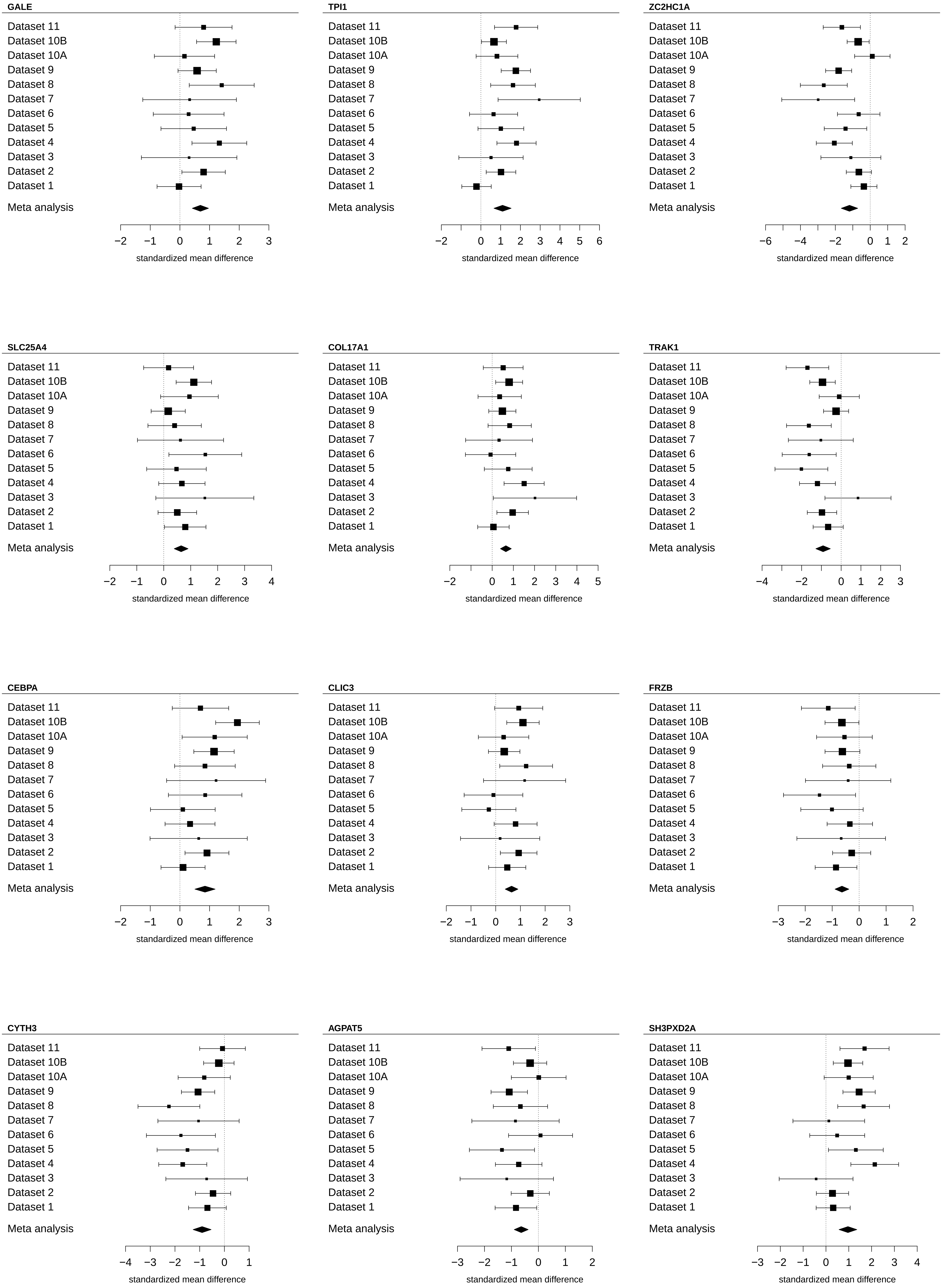
GALE
TPI1
ZC2HC1A
SLC25A4
COL17A1
TRAK1
CEBPA
CLIC3
FRZB
CYTH3
AGPAT5
SH3PXD2A
Dataset 11
Dataset 10B
Dataset 10A
Dataset 9
Dataset 8
Dataset 7
Dataset 6
Dataset 5
Dataset 4
Dataset 3
Dataset 2
Dataset 1
Meta analysis
standardized mean difference

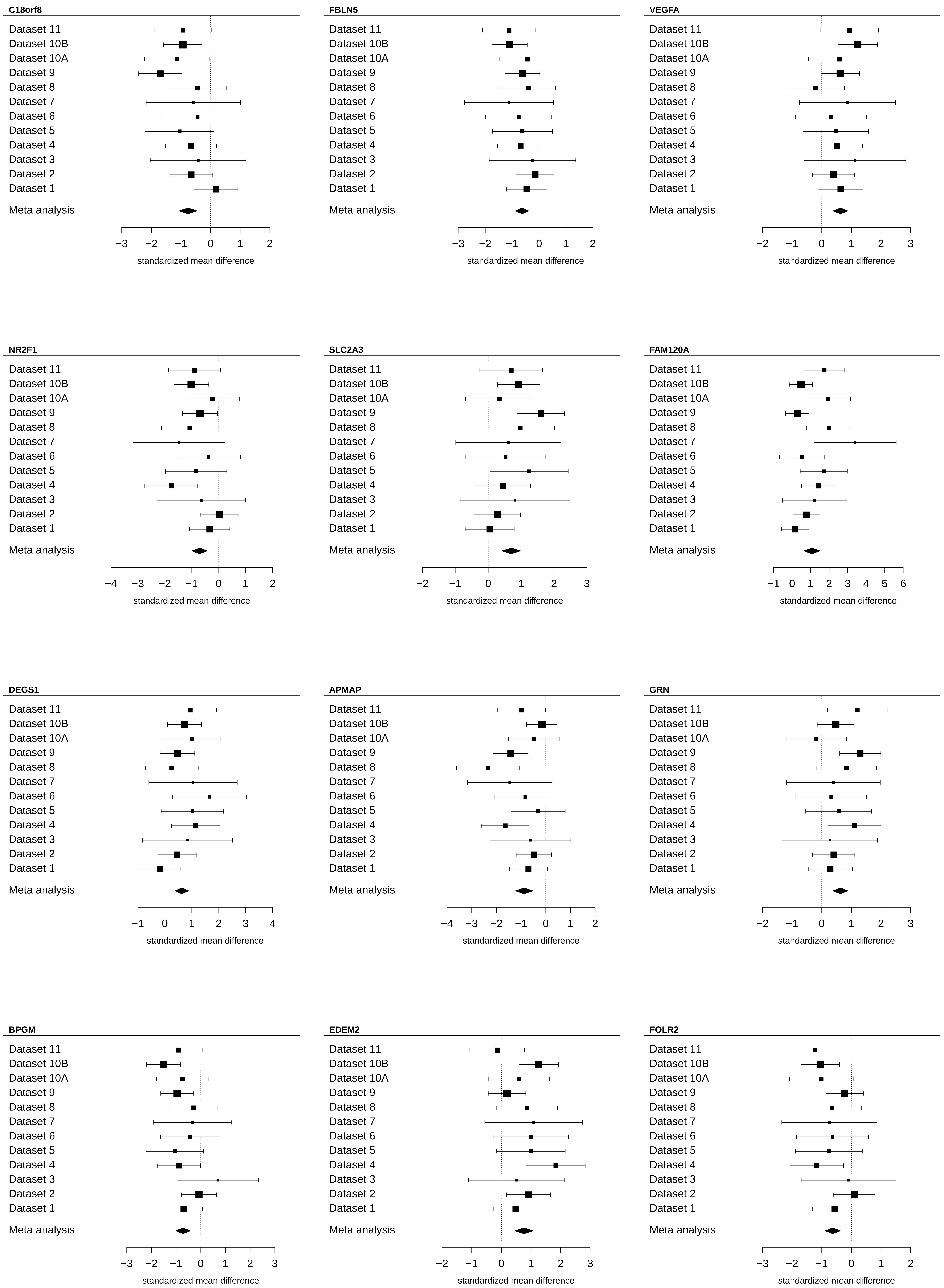

C18orf8
FBLN5
VEGFA
NR2F1
SLC2A3
FAM120A
DEGS1
APMAP
GRN
BPGM
EDEM2
FOLR2
Dataset 11
Dataset 10B
Dataset 10A
Dataset 9
Dataset 8
Dataset 7
Dataset 6
Dataset 5
Dataset 4
Dataset 3
Dataset 2
Dataset 1
Meta analysis
standardized mean difference

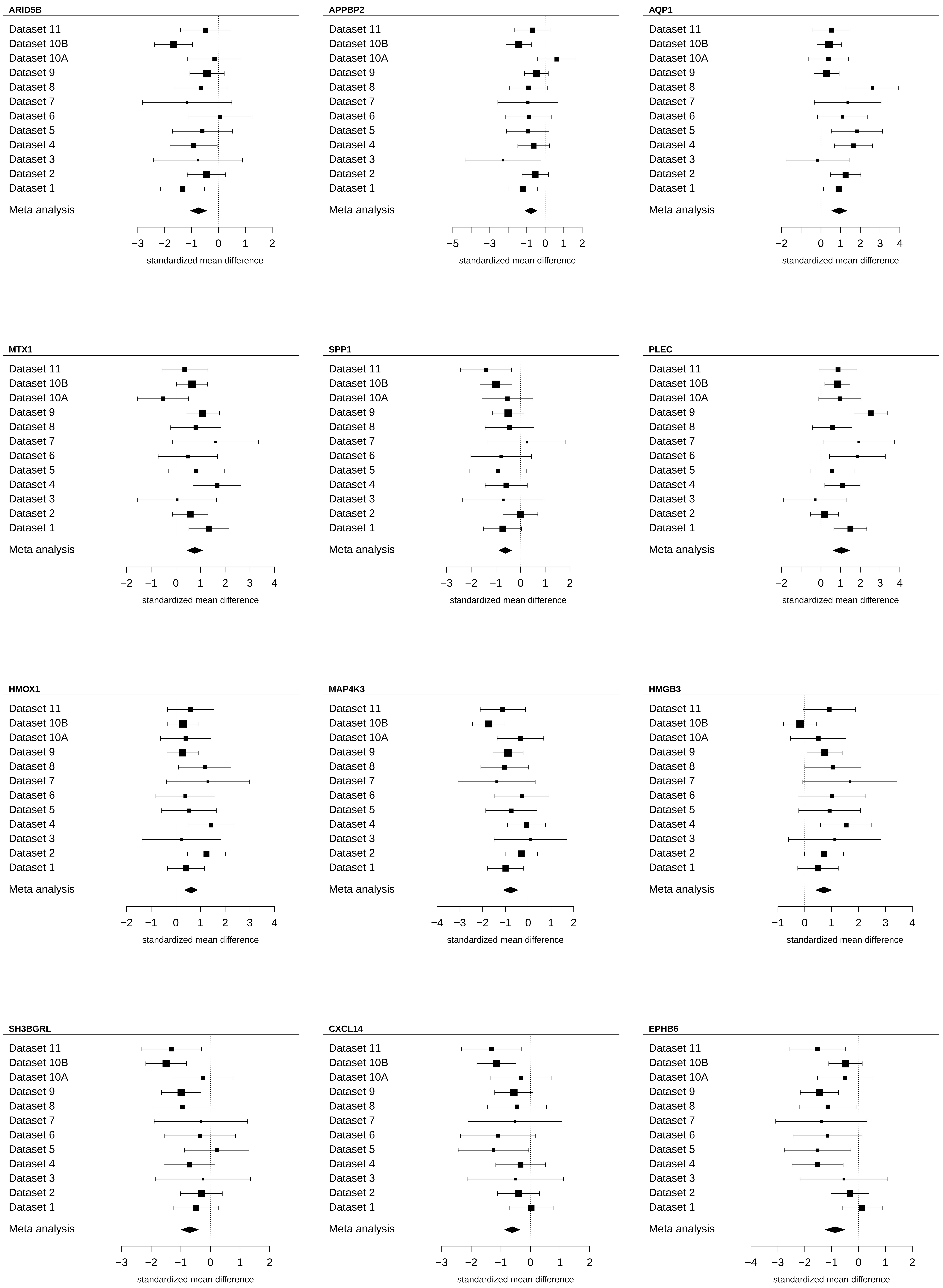
ARID5B
Dataset 11
Dataset 10B
Dataset 10A
Dataset 9
Dataset 8
Dataset 7
Dataset 6
Dataset 5
Dataset 4
Dataset 3
Dataset 2
Dataset 1
Meta analysis
−3 −2 −1 0 1 2
standardized mean difference
APPBP2
Dataset 11
Dataset 10B
Dataset 10A
Dataset 9
Dataset 8
Dataset 7
Dataset 6
Dataset 5
Dataset 4
Dataset 3
Dataset 2
Dataset 1
Meta analysis
−5 −3 −1 0 1 2
standardized mean difference
AQP1
Dataset 11
Dataset 10B
Dataset 10A
Dataset 9
Dataset 8
Dataset 7
Dataset 6
Dataset 5
Dataset 4
Dataset 3
Dataset 2
Dataset 1
Meta analysis
−2 0 1 2 3 4
standardized mean difference
MTX1
Dataset 11
Dataset 10B
Dataset 10A
Dataset 9
Dataset 8
Dataset 7
Dataset 6
Dataset 5
Dataset 4
Dataset 3
Dataset 2
Dataset 1
Meta analysis
−2 −1 0 1 2 3 4
standardized mean difference
SPP1
Dataset 11
Dataset 10B
Dataset 10A
Dataset 9
Dataset 8
Dataset 7
Dataset 6
Dataset 5
Dataset 4
Dataset 3
Dataset 2
Dataset 1
Meta analysis
−3 −2 −1 0 1 2
standardized mean difference
PLEC
Dataset 11
Dataset 10B
Dataset 10A
Dataset 9
Dataset 8
Dataset 7
Dataset 6
Dataset 5
Dataset 4
Dataset 3
Dataset 2
Dataset 1
Meta analysis
−2 0 1 2 3 4
standardized mean difference
HMOX1
Dataset 11
Dataset 10B
Dataset 10A
Dataset 9
Dataset 8
Dataset 7
Dataset 6
Dataset 5
Dataset 4
Dataset 3
Dataset 2
Dataset 1
Meta analysis
−2 −1 0 1 2 3 4
standardized mean difference
MAP4K3
Dataset 11
Dataset 10B
Dataset 10A
Dataset 9
Dataset 8
Dataset 7
Dataset 6
Dataset 5
Dataset 4
Dataset 3
Dataset 2
Dataset 1
Meta analysis
−4 −3 −2 −1 0 1 2
standardized mean difference
HMGB3
Dataset 11
Dataset 10B
Dataset 10A
Dataset 9
Dataset 8
Dataset 7
Dataset 6
Dataset 5
Dataset 4
Dataset 3
Dataset 2
Dataset 1
Meta analysis
−1 0 1 2 3 4
standardized mean difference
SH3BGRL
Dataset 11
Dataset 10B
Dataset 10A
Dataset 9
Dataset 8
Dataset 7
Dataset 6
Dataset 5
Dataset 4
Dataset 3
Dataset 2
Dataset 1
Meta analysis
−3 −2 −1 0 1 2
standardized mean difference
CXCL14
Dataset 11
Dataset 10B
Dataset 10A
Dataset 9
Dataset 8
Dataset 7
Dataset 6
Dataset 5
Dataset 4
Dataset 3
Dataset 2
Dataset 1
Meta analysis
−3 −2 −1 0 1 2
standardized mean difference
EPHB6
Dataset 11
Dataset 10B
Dataset 10A
Dataset 9
Dataset 8
Dataset 7
Dataset 6
Dataset 5
Dataset 4
Dataset 3
Dataset 2
Dataset 1
Meta analysis
−4 −3 −2 −1 0 1 2
standardized mean difference

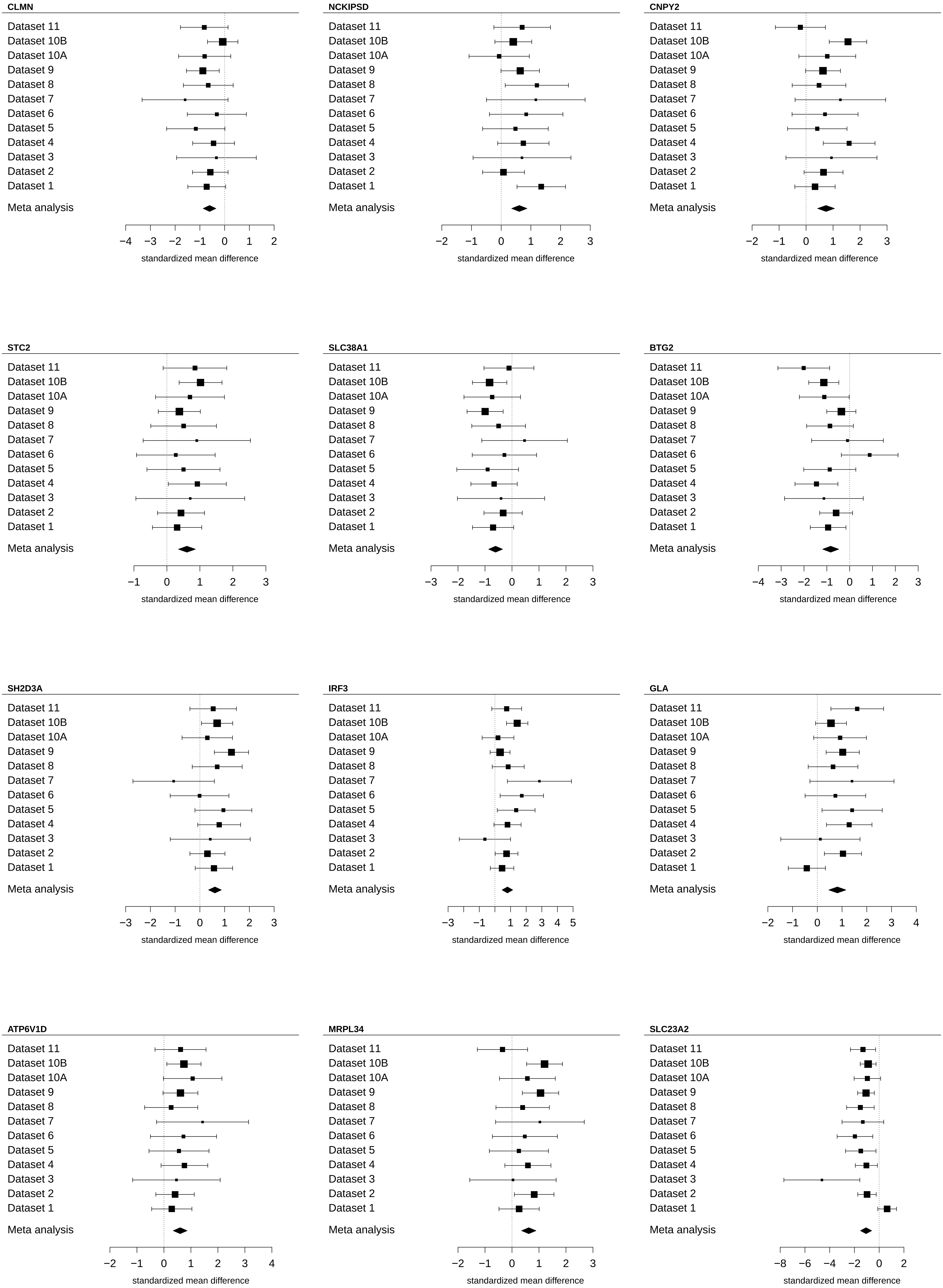
CLMN
NCKIPSD
CNPY2
STC2
SLC38A1
BTG2
SH2D3A
IRF3
GLA
ATP6V1D
MRPL34
SLC23A2
Dataset 11
Dataset 10B
Dataset 10A
Dataset 9
Dataset 8
Dataset 7
Dataset 6
Dataset 5
Dataset 4
Dataset 3
Dataset 2
Dataset 1
Meta analysis
standardized mean difference

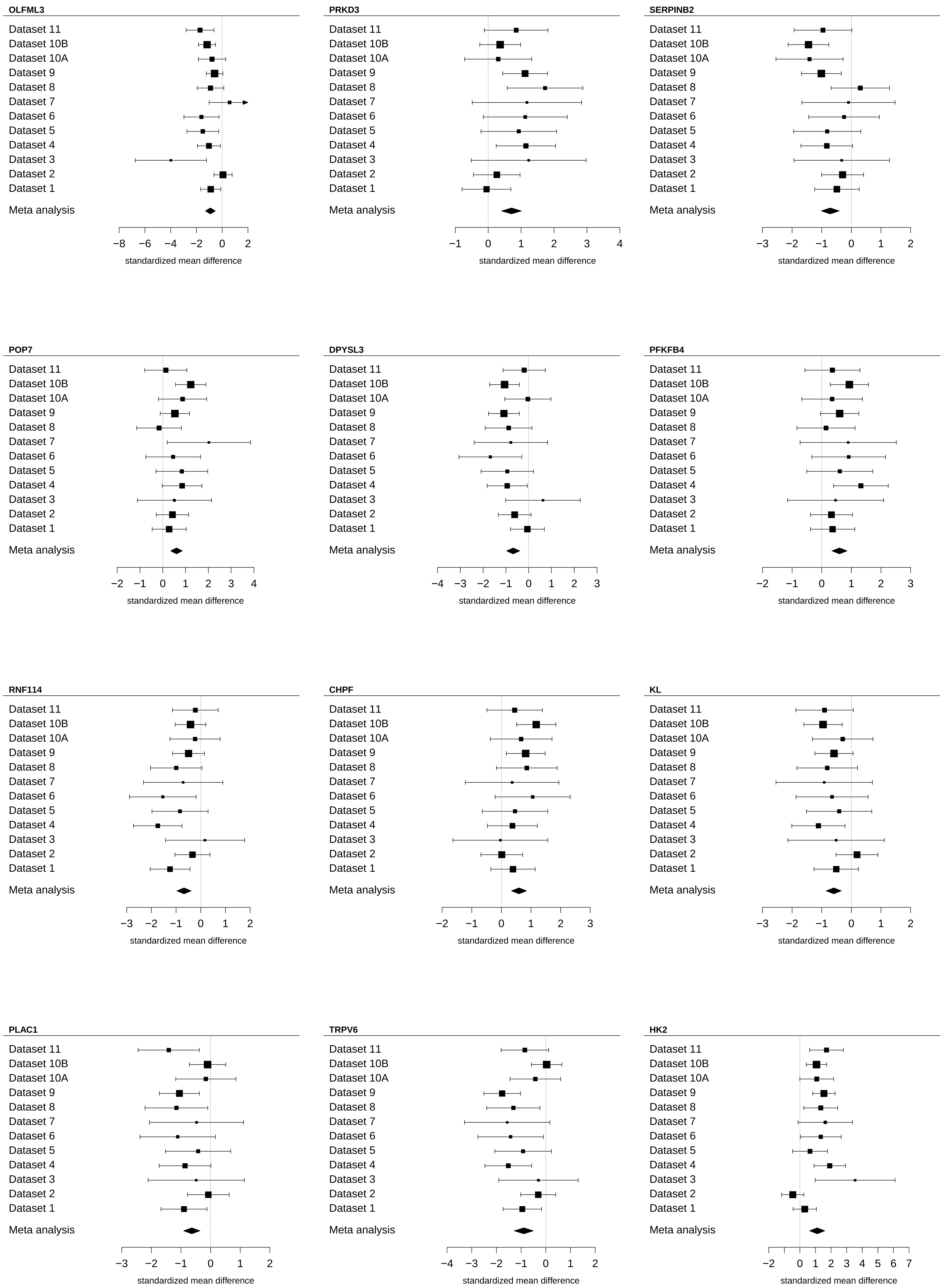
OLFML3
Dataset 11
Dataset 10B
Dataset 10A
Dataset 9
Dataset 8
Dataset 7
Dataset 6
Dataset 5
Dataset 4
Dataset 3
Dataset 2
Dataset 1
Meta analysis
−8 −6 −4 −2 0 2
standardized mean difference
PRKD3
Dataset 11
Dataset 10B
Dataset 10A
Dataset 9
Dataset 8
Dataset 7
Dataset 6
Dataset 5
Dataset 4
Dataset 3
Dataset 2
Dataset 1
Meta analysis
−1 0 1 2 3 4
standardized mean difference
SERPINB2
Dataset 11
Dataset 10B
Dataset 10A
Dataset 9
Dataset 8
Dataset 7
Dataset 6
Dataset 5
Dataset 4
Dataset 3
Dataset 2
Dataset 1
Meta analysis
−3 −2 −1 0 1 2
standardized mean difference
POP7
Dataset 11
Dataset 10B
Dataset 10A
Dataset 9
Dataset 8
Dataset 7
Dataset 6
Dataset 5
Dataset 4
Dataset 3
Dataset 2
Dataset 1
Meta analysis
−2 −1 0 1 2 3 4
standardized mean difference
DPYSL3
Dataset 11
Dataset 10B
Dataset 10A
Dataset 9
Dataset 8
Dataset 7
Dataset 6
Dataset 5
Dataset 4
Dataset 3
Dataset 2
Dataset 1
Meta analysis
−4 −3 −2 −1 0 1 2 3
standardized mean difference
PFKFB4
Dataset 11
Dataset 10B
Dataset 10A
Dataset 9
Dataset 8
Dataset 7
Dataset 6
Dataset 5
Dataset 4
Dataset 3
Dataset 2
Dataset 1
Meta analysis
−2 −1 0 1 2 3
standardized mean difference
RNF114
Dataset 11
Dataset 10B
Dataset 10A
Dataset 9
Dataset 8
Dataset 7
Dataset 6
Dataset 5
Dataset 4
Dataset 3
Dataset 2
Dataset 1
Meta analysis
−3 −2 −1 0 1 2
standardized mean difference
CHPF
Dataset 11
Dataset 10B
Dataset 10A
Dataset 9
Dataset 8
Dataset 7
Dataset 6
Dataset 5
Dataset 4
Dataset 3
Dataset 2
Dataset 1
Meta analysis
−2 −1 0 1 2 3
standardized mean difference
KL
Dataset 11
Dataset 10B
Dataset 10A
Dataset 9
Dataset 8
Dataset 7
Dataset 6
Dataset 5
Dataset 4
Dataset 3
Dataset 2
Dataset 1
Meta analysis
−3 −2 −1 0 1 2
standardized mean difference
PLAC1
Dataset 11
Dataset 10B
Dataset 10A
Dataset 9
Dataset 8
Dataset 7
Dataset 6
Dataset 5
Dataset 4
Dataset 3
Dataset 2
Dataset 1
Meta analysis
−3 −2 −1 0 1 2
standardized mean difference
TRPV6
Dataset 11
Dataset 10B
Dataset 10A
Dataset 9
Dataset 8
Dataset 7
Dataset 6
Dataset 5
Dataset 4
Dataset 3
Dataset 2
Dataset 1
Meta analysis
−4 −3 −2 −1 0 1 2
standardized mean difference
HK2
Dataset 11
Dataset 10B
Dataset 10A
Dataset 9
Dataset 8
Dataset 7
Dataset 6
Dataset 5
Dataset 4
Dataset 3
Dataset 2
Dataset 1
Meta analysis
−2 0 1 2 3 4 5 6 7
standardized mean difference

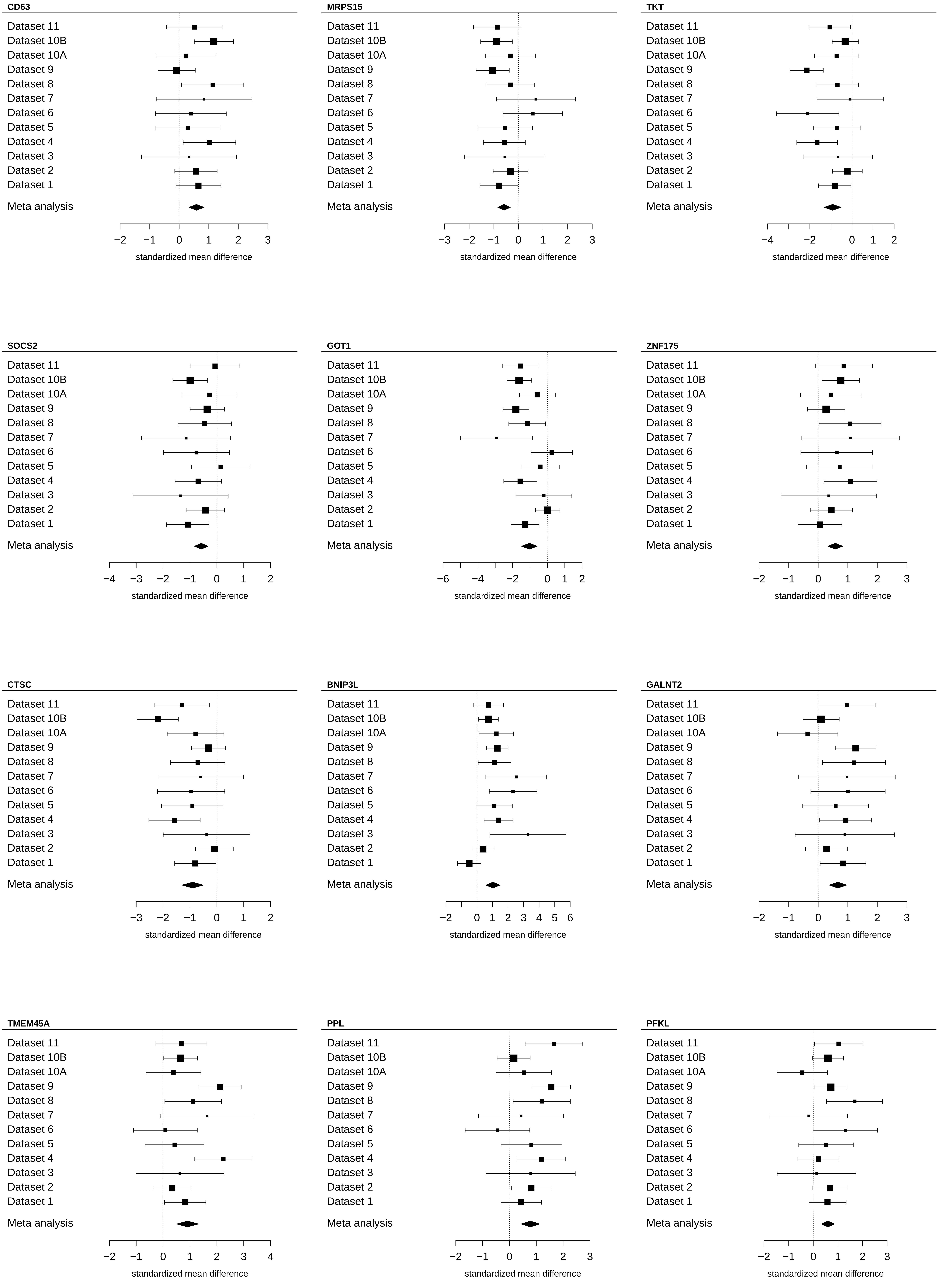
CD63
Dataset 11
Dataset 10B
Dataset 10A
Dataset 9
Dataset 8
Dataset 7
Dataset 6
Dataset 5
Dataset 4
Dataset 3
Dataset 2
Dataset 1
Meta analysis
−2 −1 0 1 2 3
standardized mean difference
MRPS15
Dataset 11
Dataset 10B
Dataset 10A
Dataset 9
Dataset 8
Dataset 7
Dataset 6
Dataset 5
Dataset 4
Dataset 3
Dataset 2
Dataset 1
Meta analysis
−3 −2 −1 0 1 2 3
standardized mean difference
TKT
Dataset 11
Dataset 10B
Dataset 10A
Dataset 9
Dataset 8
Dataset 7
Dataset 6
Dataset 5
Dataset 4
Dataset 3
Dataset 2
Dataset 1
Meta analysis
−4 −2 0 1 2
standardized mean difference
SOCS2
Dataset 11
Dataset 10B
Dataset 10A
Dataset 9
Dataset 8
Dataset 7
Dataset 6
Dataset 5
Dataset 4
Dataset 3
Dataset 2
Dataset 1
Meta analysis
−4 −3 −2 −1 0 1 2
standardized mean difference
GOT1
Dataset 11
Dataset 10B
Dataset 10A
Dataset 9
Dataset 8
Dataset 7
Dataset 6
Dataset 5
Dataset 4
Dataset 3
Dataset 2
Dataset 1
Meta analysis
−6 −4 −2 0 1 2
standardized mean difference
ZNF175
Dataset 11
Dataset 10B
Dataset 10A
Dataset 9
Dataset 8
Dataset 7
Dataset 6
Dataset 5
Dataset 4
Dataset 3
Dataset 2
Dataset 1
Meta analysis
−2 −1 0 1 2 3
standardized mean difference
CTSC
Dataset 11
Dataset 10B
Dataset 10A
Dataset 9
Dataset 8
Dataset 7
Dataset 6
Dataset 5
Dataset 4
Dataset 3
Dataset 2
Dataset 1
Meta analysis
−3 −2 −1 0 1 2
standardized mean difference
BNIP3L
Dataset 11
Dataset 10B
Dataset 10A
Dataset 9
Dataset 8
Dataset 7
Dataset 6
Dataset 5
Dataset 4
Dataset 3
Dataset 2
Dataset 1
Meta analysis
−2 0 1 2 3 4 5 6
standardized mean difference
GALNT2
Dataset 11
Dataset 10B
Dataset 10A
Dataset 9
Dataset 8
Dataset 7
Dataset 6
Dataset 5
Dataset 4
Dataset 3
Dataset 2
Dataset 1
Meta analysis
−2 −1 0 1 2 3
standardized mean difference
TMEM45A
Dataset 11
Dataset 10B
Dataset 10A
Dataset 9
Dataset 8
Dataset 7
Dataset 6
Dataset 5
Dataset 4
Dataset 3
Dataset 2
Dataset 1
Meta analysis
−2 −1 0 1 2 3 4
standardized mean difference
PPL
Dataset 11
Dataset 10B
Dataset 10A
Dataset 9
Dataset 8
Dataset 7
Dataset 6
Dataset 5
Dataset 4
Dataset 3
Dataset 2
Dataset 1
Meta analysis
−2 −1 0 1 2 3
standardized mean difference
PFKL
Dataset 11
Dataset 10B
Dataset 10A
Dataset 9
Dataset 8
Dataset 7
Dataset 6
Dataset 5
Dataset 4
Dataset 3
Dataset 2
Dataset 1
Meta analysis
−2 −1 0 1 2 3
standardized mean difference

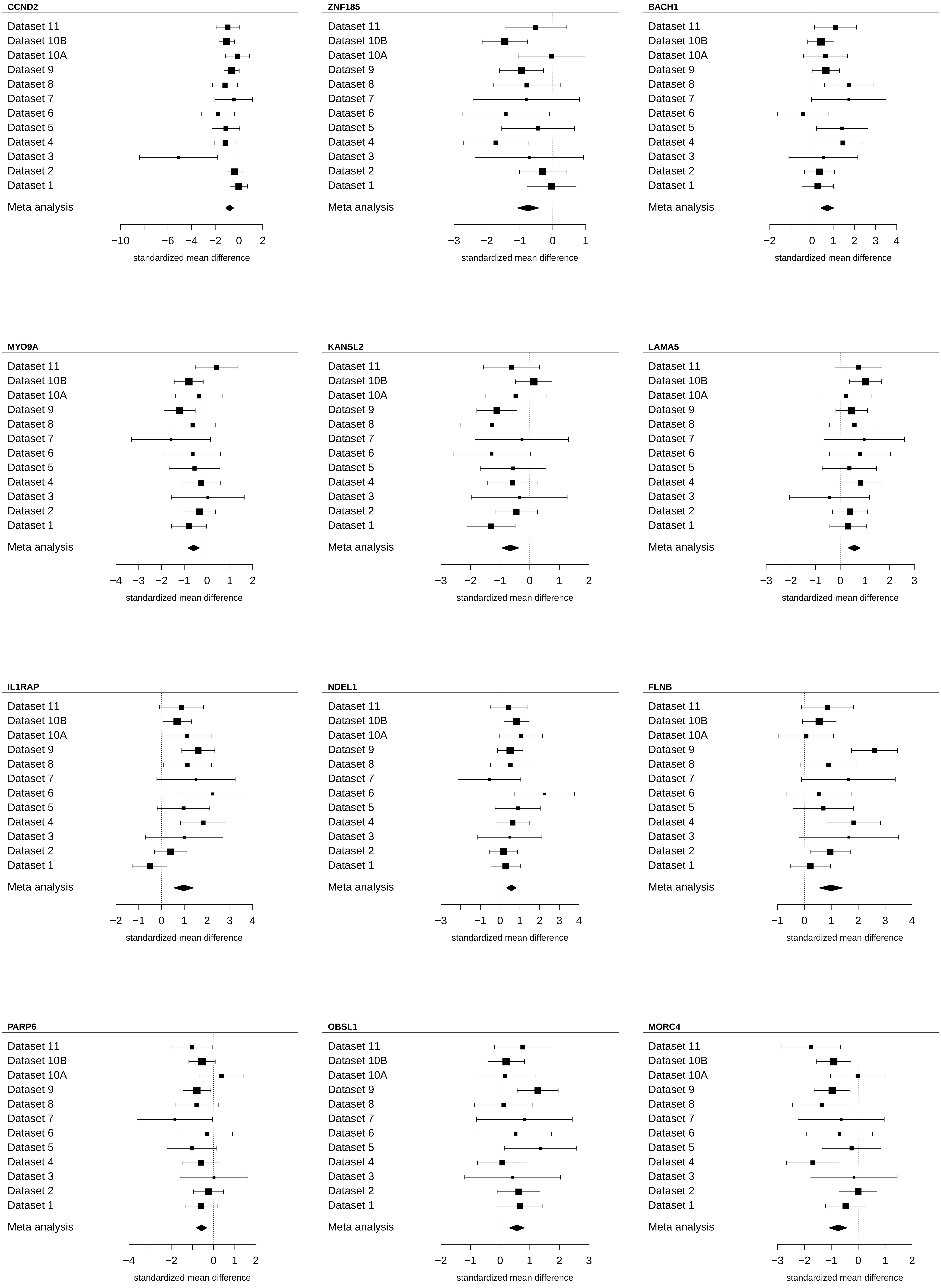
CCND2
ZNF185
BACH1
MYO9A
KANSL2
LAMA5
IL1RAP
NDEL1
FLNB
PARP6
OBSL1
MORC4
Dataset 11
Dataset 10B
Dataset 10A
Dataset 9
Dataset 8
Dataset 7
Dataset 6
Dataset 5
Dataset 4
Dataset 3
Dataset 2
Dataset 1
Meta analysis
−10 −6 −4 −2 0 2
−3 −2 −1 0 1
−2 0 1 2 3 4
−4 −3 −2 −1 0 1 2
−3 −2 −1 0 1 2
−3 −2 −1 0 1 2 3
−2 −1 0 1 2 3 4
−3 −1 0 1 2 3 4
−1 0 1 2 3 4
−4 −2 0 1 2
−2 −1 0 1 2 3
−3 −2 −1 0 1 2
standardized mean difference

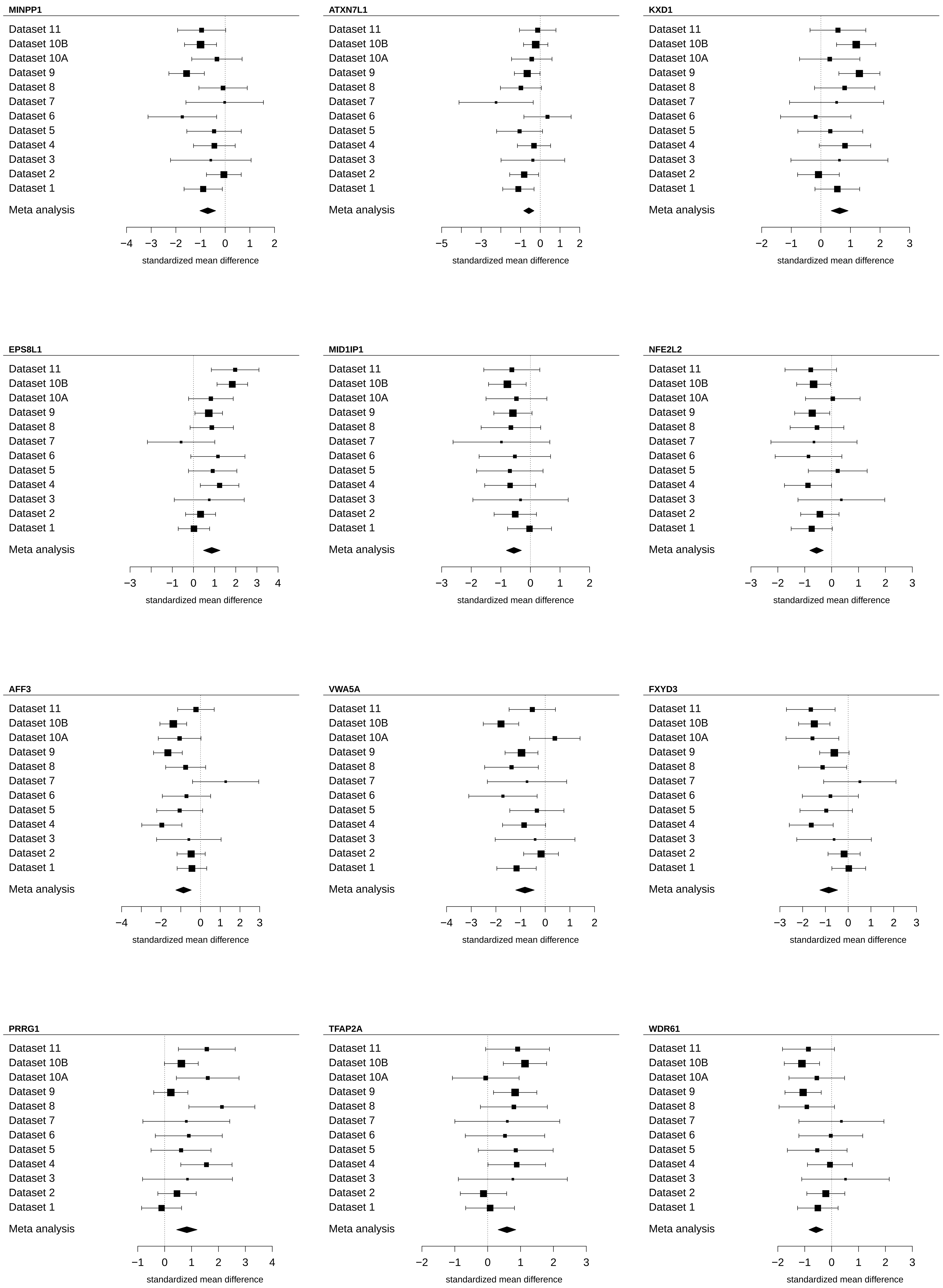
MINPP1
Dataset 11
Dataset 10B
Dataset 10A
Dataset 9
Dataset 8
Dataset 7
Dataset 6
Dataset 5
Dataset 4
Dataset 3
Dataset 2
Dataset 1
Meta analysis
−4 −3 −2 −1 0 1 2
standardized mean difference
ATXN7L1
Dataset 11
Dataset 10B
Dataset 10A
Dataset 9
Dataset 8
Dataset 7
Dataset 6
Dataset 5
Dataset 4
Dataset 3
Dataset 2
Dataset 1
Meta analysis
−5 −3 −1 0 1 2
standardized mean difference
KXD1
Dataset 11
Dataset 10B
Dataset 10A
Dataset 9
Dataset 8
Dataset 7
Dataset 6
Dataset 5
Dataset 4
Dataset 3
Dataset 2
Dataset 1
Meta analysis
−2 −1 0 1 2 3
standardized mean difference
EPS8L1
Dataset 11
Dataset 10B
Dataset 10A
Dataset 9
Dataset 8
Dataset 7
Dataset 6
Dataset 5
Dataset 4
Dataset 3
Dataset 2
Dataset 1
Meta analysis
−3 −1 0 1 2 3 4
standardized mean difference
MID1IP1
Dataset 11
Dataset 10B
Dataset 10A
Dataset 9
Dataset 8
Dataset 7
Dataset 6
Dataset 5
Dataset 4
Dataset 3
Dataset 2
Dataset 1
Meta analysis
−3 −2 −1 0 1 2
standardized mean difference
NFE2L2
Dataset 11
Dataset 10B
Dataset 10A
Dataset 9
Dataset 8
Dataset 7
Dataset 6
Dataset 5
Dataset 4
Dataset 3
Dataset 2
Dataset 1
Meta analysis
−3 −2 −1 0 1 2 3
standardized mean difference
AFF3
Dataset 11
Dataset 10B
Dataset 10A
Dataset 9
Dataset 8
Dataset 7
Dataset 6
Dataset 5
Dataset 4
Dataset 3
Dataset 2
Dataset 1
Meta analysis
−4 −2 0 1 2 3
standardized mean difference
VWA5A
Dataset 11
Dataset 10B
Dataset 10A
Dataset 9
Dataset 8
Dataset 7
Dataset 6
Dataset 5
Dataset 4
Dataset 3
Dataset 2
Dataset 1
Meta analysis
−4 −3 −2 −1 0 1 2
standardized mean difference
FXYD3
Dataset 11
Dataset 10B
Dataset 10A
Dataset 9
Dataset 8
Dataset 7
Dataset 6
Dataset 5
Dataset 4
Dataset 3
Dataset 2
Dataset 1
Meta analysis
−3 −2 −1 0 1 2 3
standardized mean difference
PRRG1
Dataset 11
Dataset 10B
Dataset 10A
Dataset 9
Dataset 8
Dataset 7
Dataset 6
Dataset 5
Dataset 4
Dataset 3
Dataset 2
Dataset 1
Meta analysis
−1 0 1 2 3 4
standardized mean difference
TFAP2A
Dataset 11
Dataset 10B
Dataset 10A
Dataset 9
Dataset 8
Dataset 7
Dataset 6
Dataset 5
Dataset 4
Dataset 3
Dataset 2
Dataset 1
Meta analysis
−2 −1 0 1 2 3
standardized mean difference
WDR61
Dataset 11
Dataset 10B
Dataset 10A
Dataset 9
Dataset 8
Dataset 7
Dataset 6
Dataset 5
Dataset 4
Dataset 3
Dataset 2
Dataset 1
Meta analysis
−2 −1 0 1 2 3
standardized mean difference

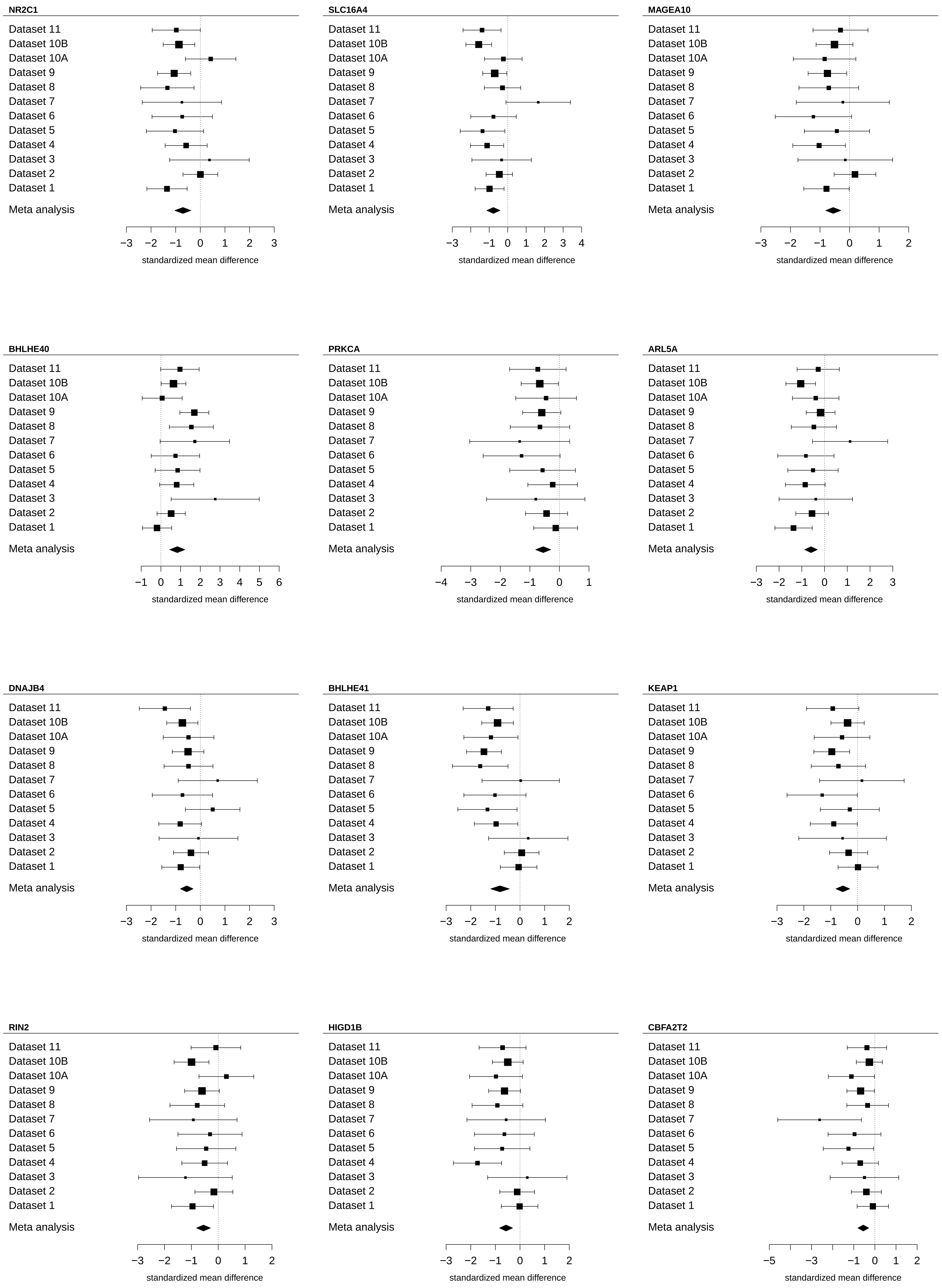
NR2C1
Dataset 11
Dataset 10B
Dataset 10A
Dataset 9
Dataset 8
Dataset 7
Dataset 6
Dataset 5
Dataset 4
Dataset 3
Dataset 2
Dataset 1
Meta analysis
−3 −2 −1 0 1 2 3
standardized mean difference
SLC16A4
Dataset 11
Dataset 10B
Dataset 10A
Dataset 9
Dataset 8
Dataset 7
Dataset 6
Dataset 5
Dataset 4
Dataset 3
Dataset 2
Dataset 1
Meta analysis
−3 −1 0 1 2 3 4
standardized mean difference
MAGEA10
Dataset 11
Dataset 10B
Dataset 10A
Dataset 9
Dataset 8
Dataset 7
Dataset 6
Dataset 5
Dataset 4
Dataset 3
Dataset 2
Dataset 1
Meta analysis
−3 −2 −1 0 1 2
standardized mean difference
BHLHE40
Dataset 11
Dataset 10B
Dataset 10A
Dataset 9
Dataset 8
Dataset 7
Dataset 6
Dataset 5
Dataset 4
Dataset 3
Dataset 2
Dataset 1
Meta analysis
−1 0 1 2 3 4 5 6
standardized mean difference
PRKCA
Dataset 11
Dataset 10B
Dataset 10A
Dataset 9
Dataset 8
Dataset 7
Dataset 6
Dataset 5
Dataset 4
Dataset 3
Dataset 2
Dataset 1
Meta analysis
−4 −3 −2 −1 0 1
standardized mean difference
ARL5A
Dataset 11
Dataset 10B
Dataset 10A
Dataset 9
Dataset 8
Dataset 7
Dataset 6
Dataset 5
Dataset 4
Dataset 3
Dataset 2
Dataset 1
Meta analysis
−3 −2 −1 0 1 2 3
standardized mean difference
DNAJB4
Dataset 11
Dataset 10B
Dataset 10A
Dataset 9
Dataset 8
Dataset 7
Dataset 6
Dataset 5
Dataset 4
Dataset 3
Dataset 2
Dataset 1
Meta analysis
−3 −2 −1 0 1 2 3
standardized mean difference
BHLHE41
Dataset 11
Dataset 10B
Dataset 10A
Dataset 9
Dataset 8
Dataset 7
Dataset 6
Dataset 5
Dataset 4
Dataset 3
Dataset 2
Dataset 1
Meta analysis
−3 −2 −1 0 1 2
standardized mean difference
KEAP1
Dataset 11
Dataset 10B
Dataset 10A
Dataset 9
Dataset 8
Dataset 7
Dataset 6
Dataset 5
Dataset 4
Dataset 3
Dataset 2
Dataset 1
Meta analysis
−3 −2 −1 0 1 2
standardized mean difference
RIN2
Dataset 11
Dataset 10B
Dataset 10A
Dataset 9
Dataset 8
Dataset 7
Dataset 6
Dataset 5
Dataset 4
Dataset 3
Dataset 2
Dataset 1
Meta analysis
−3 −2 −1 0 1 2
standardized mean difference
HIGD1B
Dataset 11
Dataset 10B
Dataset 10A
Dataset 9
Dataset 8
Dataset 7
Dataset 6
Dataset 5
Dataset 4
Dataset 3
Dataset 2
Dataset 1
Meta analysis
−3 −2 −1 0 1 2
standardized mean difference
CBFA2T2
Dataset 11
Dataset 10B
Dataset 10A
Dataset 9
Dataset 8
Dataset 7
Dataset 6
Dataset 5
Dataset 4
Dataset 3
Dataset 2
Dataset 1
Meta analysis
−5 −3 −1 0 1 2
standardized mean difference

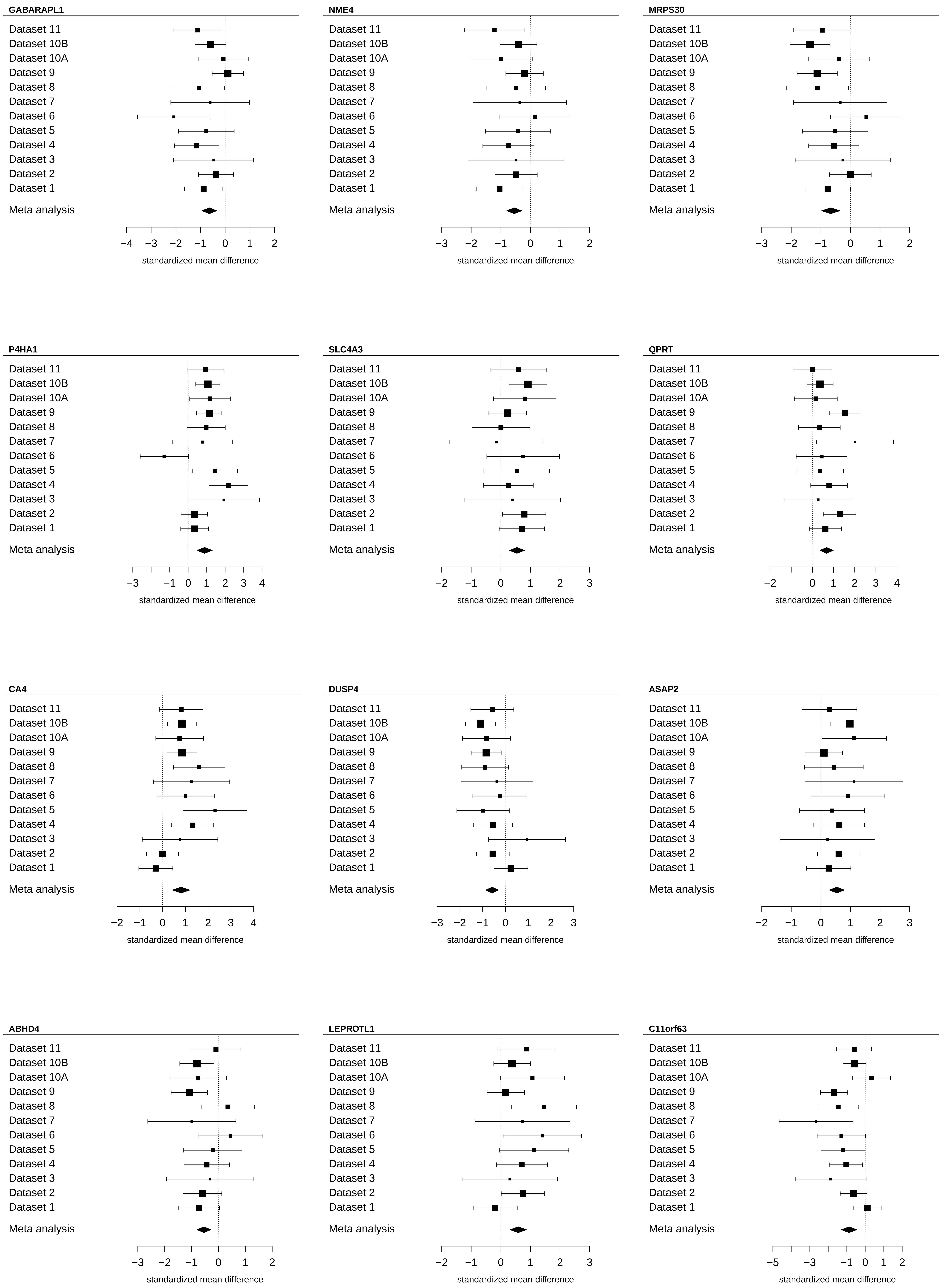
GABARAPL1
NME4
MRPS30
P4HA1
SLC4A3
QPRT
CA4
DUSP4
ASAP2
ABHD4
LEPROTL1
C11orf63
Dataset 11
Dataset 10B
Dataset 10A
Dataset 9
Dataset 8
Dataset 7
Dataset 6
Dataset 5
Dataset 4
Dataset 3
Dataset 2
Dataset 1
Meta analysis
standardized mean difference

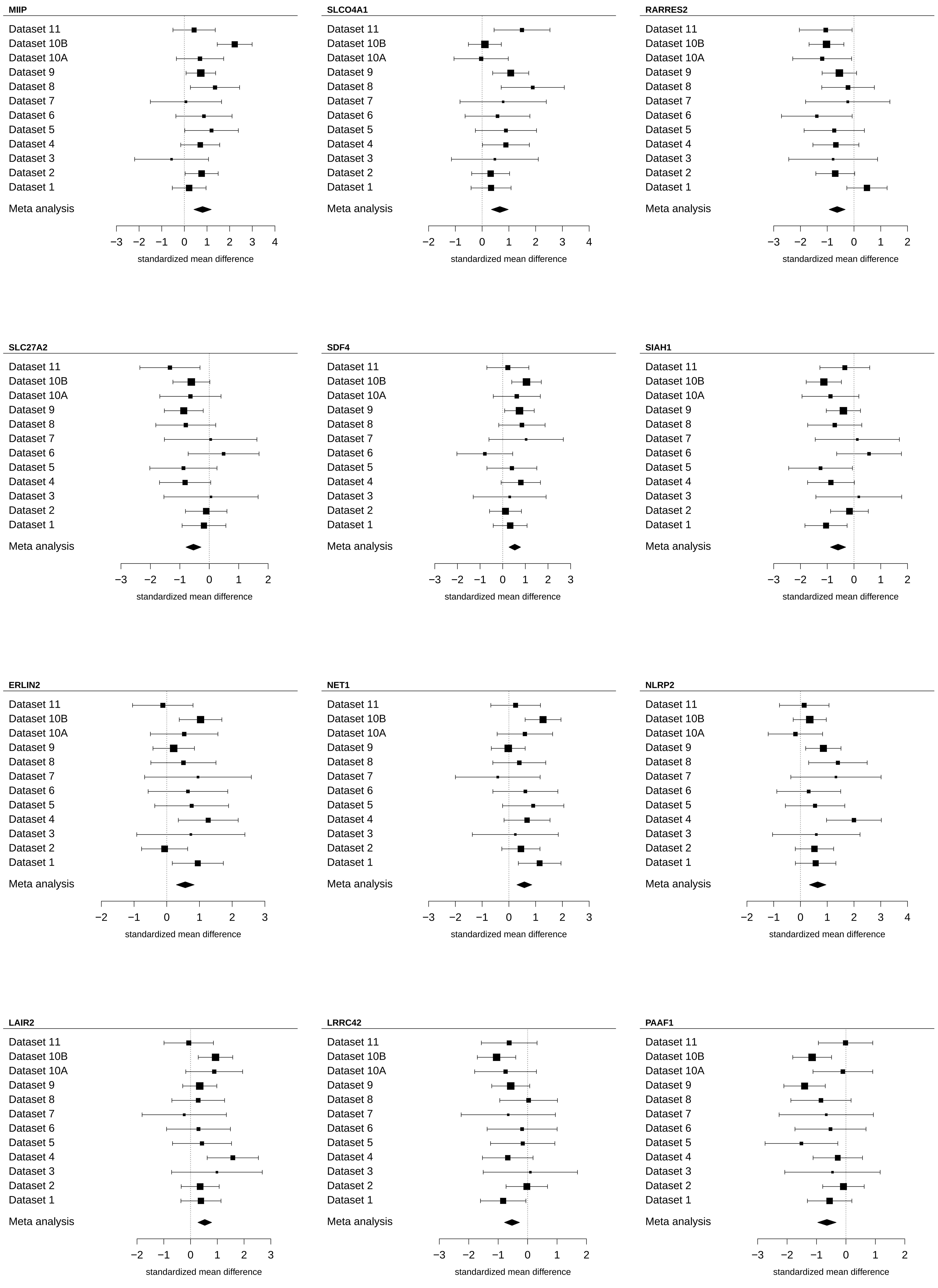

MIIP
SLCO4A1
RARRES2
SLC27A2
SDF4
SIAH1
ERLIN2
NET1
NLRP2
LAIR2
LRRC42
PAAF1
Dataset 11
Dataset 10B
Dataset 10A
Dataset 9
Dataset 8
Dataset 7
Dataset 6
Dataset 5
Dataset 4
Dataset 3
Dataset 2
Dataset 1
Meta analysis
−3 −2 −1 0 1 2 3 4
−2 −1 0 1 2 3 4
−3 −2 −1 0 1 2
−3 −2 −1 0 1 2 3
−2 −1 0 1 2 3
−3 −2 −1 0 1 2 3
−2 −1 0 1 2 3 4
−2 −1 0 1 2 3
−3 −2 −1 0 1 2
standardized mean difference

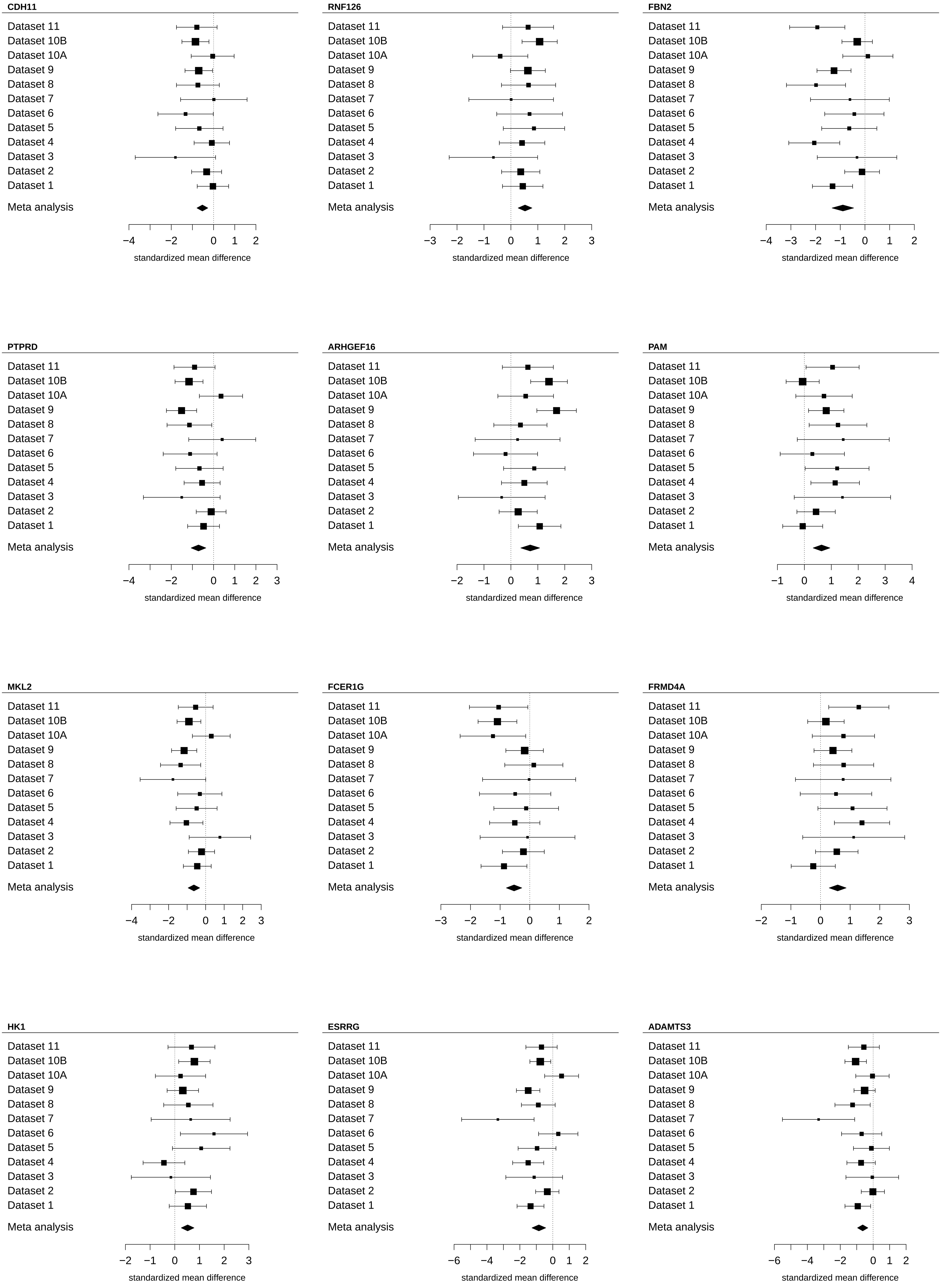
CDH11
Dataset 11
Dataset 10B
Dataset 10A
Dataset 9
Dataset 8
Dataset 7
Dataset 6
Dataset 5
Dataset 4
Dataset 3
Dataset 2
Dataset 1
Meta analysis
−4 −2 0 1 2
standardized mean difference
RNF126
Dataset 11
Dataset 10B
Dataset 10A
Dataset 9
Dataset 8
Dataset 7
Dataset 6
Dataset 5
Dataset 4
Dataset 3
Dataset 2
Dataset 1
Meta analysis
−3 −2 −1 0 1 2 3
standardized mean difference
FBN2
Dataset 11
Dataset 10B
Dataset 10A
Dataset 9
Dataset 8
Dataset 7
Dataset 6
Dataset 5
Dataset 4
Dataset 3
Dataset 2
Dataset 1
Meta analysis
−4 −3 −2 −1 0 1 2
standardized mean difference
PTPRD
Dataset 11
Dataset 10B
Dataset 10A
Dataset 9
Dataset 8
Dataset 7
Dataset 6
Dataset 5
Dataset 4
Dataset 3
Dataset 2
Dataset 1
Meta analysis
−4 −2 0 1 2 3
standardized mean difference
ARHGEF16
Dataset 11
Dataset 10B
Dataset 10A
Dataset 9
Dataset 8
Dataset 7
Dataset 6
Dataset 5
Dataset 4
Dataset 3
Dataset 2
Dataset 1
Meta analysis
−2 −1 0 1 2 3
standardized mean difference
PAM
Dataset 11
Dataset 10B
Dataset 10A
Dataset 9
Dataset 8
Dataset 7
Dataset 6
Dataset 5
Dataset 4
Dataset 3
Dataset 2
Dataset 1
Meta analysis
−1 0 1 2 3 4
standardized mean difference
MKL2
Dataset 11
Dataset 10B
Dataset 10A
Dataset 9
Dataset 8
Dataset 7
Dataset 6
Dataset 5
Dataset 4
Dataset 3
Dataset 2
Dataset 1
Meta analysis
−4 −2 0 1 2 3
standardized mean difference
FCER1G
Dataset 11
Dataset 10B
Dataset 10A
Dataset 9
Dataset 8
Dataset 7
Dataset 6
Dataset 5
Dataset 4
Dataset 3
Dataset 2
Dataset 1
Meta analysis
−3 −2 −1 0 1 2
standardized mean difference
FRMD4A
Dataset 11
Dataset 10B
Dataset 10A
Dataset 9
Dataset 8
Dataset 7
Dataset 6
Dataset 5
Dataset 4
Dataset 3
Dataset 2
Dataset 1
Meta analysis
−2 −1 0 1 2 3
standardized mean difference
HK1
Dataset 11
Dataset 10B
Dataset 10A
Dataset 9
Dataset 8
Dataset 7
Dataset 6
Dataset 5
Dataset 4
Dataset 3
Dataset 2
Dataset 1
Meta analysis
−2 −1 0 1 2 3
standardized mean difference
ESRRG
Dataset 11
Dataset 10B
Dataset 10A
Dataset 9
Dataset 8
Dataset 7
Dataset 6
Dataset 5
Dataset 4
Dataset 3
Dataset 2
Dataset 1
Meta analysis
−6 −4 −2 0 1 2
standardized mean difference
ADAMTS3
Dataset 11
Dataset 10B
Dataset 10A
Dataset 9
Dataset 8
Dataset 7
Dataset 6
Dataset 5
Dataset 4
Dataset 3
Dataset 2
Dataset 1
Meta analysis
−6 −4 −2 0 2
standardized mean difference

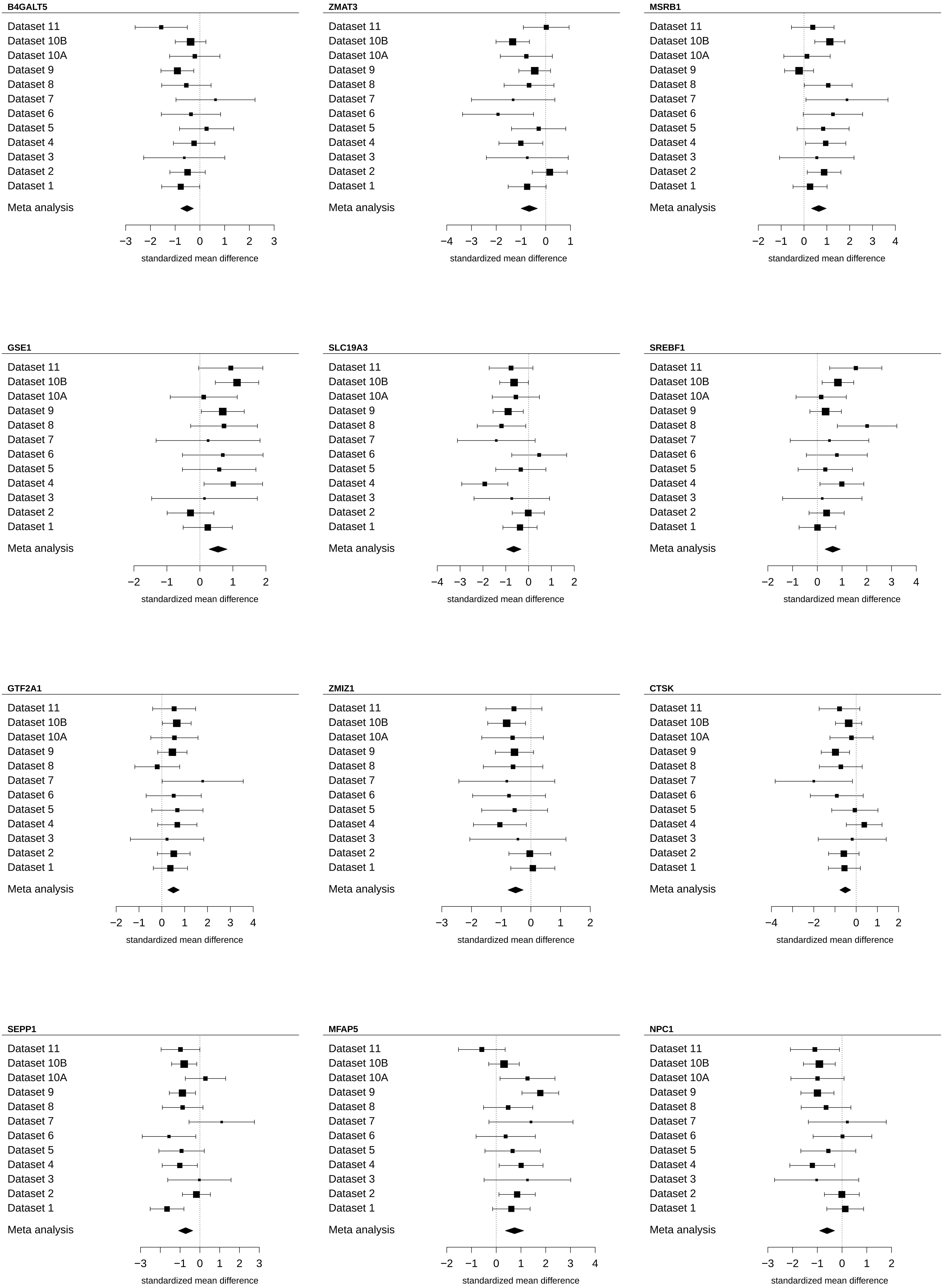
B4GALT5
Dataset 11
Dataset 10B
Dataset 10A
Dataset 9
Dataset 8
Dataset 7
Dataset 6
Dataset 5
Dataset 4
Dataset 3
Dataset 2
Dataset 1
Meta analysis
−3 −2 −1 0 1 2 3
standardized mean difference
ZMAT3
Dataset 11
Dataset 10B
Dataset 10A
Dataset 9
Dataset 8
Dataset 7
Dataset 6
Dataset 5
Dataset 4
Dataset 3
Dataset 2
Dataset 1
Meta analysis
−4 −3 −2 −1 0 1
standardized mean difference
MSRB1
Dataset 11
Dataset 10B
Dataset 10A
Dataset 9
Dataset 8
Dataset 7
Dataset 6
Dataset 5
Dataset 4
Dataset 3
Dataset 2
Dataset 1
Meta analysis
−2 −1 0 1 2 3 4
standardized mean difference
GSE1
Dataset 11
Dataset 10B
Dataset 10A
Dataset 9
Dataset 8
Dataset 7
Dataset 6
Dataset 5
Dataset 4
Dataset 3
Dataset 2
Dataset 1
Meta analysis
−2 −1 0 1 2
standardized mean difference
SLC19A3
Dataset 11
Dataset 10B
Dataset 10A
Dataset 9
Dataset 8
Dataset 7
Dataset 6
Dataset 5
Dataset 4
Dataset 3
Dataset 2
Dataset 1
Meta analysis
−4 −3 −2 −1 0 1 2
standardized mean difference
SREBF1
Dataset 11
Dataset 10B
Dataset 10A
Dataset 9
Dataset 8
Dataset 7
Dataset 6
Dataset 5
Dataset 4
Dataset 3
Dataset 2
Dataset 1
Meta analysis
−2 −1 0 1 2 3 4
standardized mean difference
GTF2A1
Dataset 11
Dataset 10B
Dataset 10A
Dataset 9
Dataset 8
Dataset 7
Dataset 6
Dataset 5
Dataset 4
Dataset 3
Dataset 2
Dataset 1
Meta analysis
−2 −1 0 1 2 3 4
standardized mean difference
ZMIZ1
Dataset 11
Dataset 10B
Dataset 10A
Dataset 9
Dataset 8
Dataset 7
Dataset 6
Dataset 5
Dataset 4
Dataset 3
Dataset 2
Dataset 1
Meta analysis
−3 −2 −1 0 1 2
standardized mean difference
CTSK
Dataset 11
Dataset 10B
Dataset 10A
Dataset 9
Dataset 8
Dataset 7
Dataset 6
Dataset 5
Dataset 4
Dataset 3
Dataset 2
Dataset 1
Meta analysis
−4 −2 0 1 2
standardized mean difference
SEPP1
Dataset 11
Dataset 10B
Dataset 10A
Dataset 9
Dataset 8
Dataset 7
Dataset 6
Dataset 5
Dataset 4
Dataset 3
Dataset 2
Dataset 1
Meta analysis
−3 −1 0 1 2 3
standardized mean difference
MFAP5
Dataset 11
Dataset 10B
Dataset 10A
Dataset 9
Dataset 8
Dataset 7
Dataset 6
Dataset 5
Dataset 4
Dataset 3
Dataset 2
Dataset 1
Meta analysis
−2 −1 0 1 2 3 4
standardized mean difference
NPC1
Dataset 11
Dataset 10B
Dataset 10A
Dataset 9
Dataset 8
Dataset 7
Dataset 6
Dataset 5
Dataset 4
Dataset 3
Dataset 2
Dataset 1
Meta analysis
−3 −2 −1 0 1 2
standardized mean difference

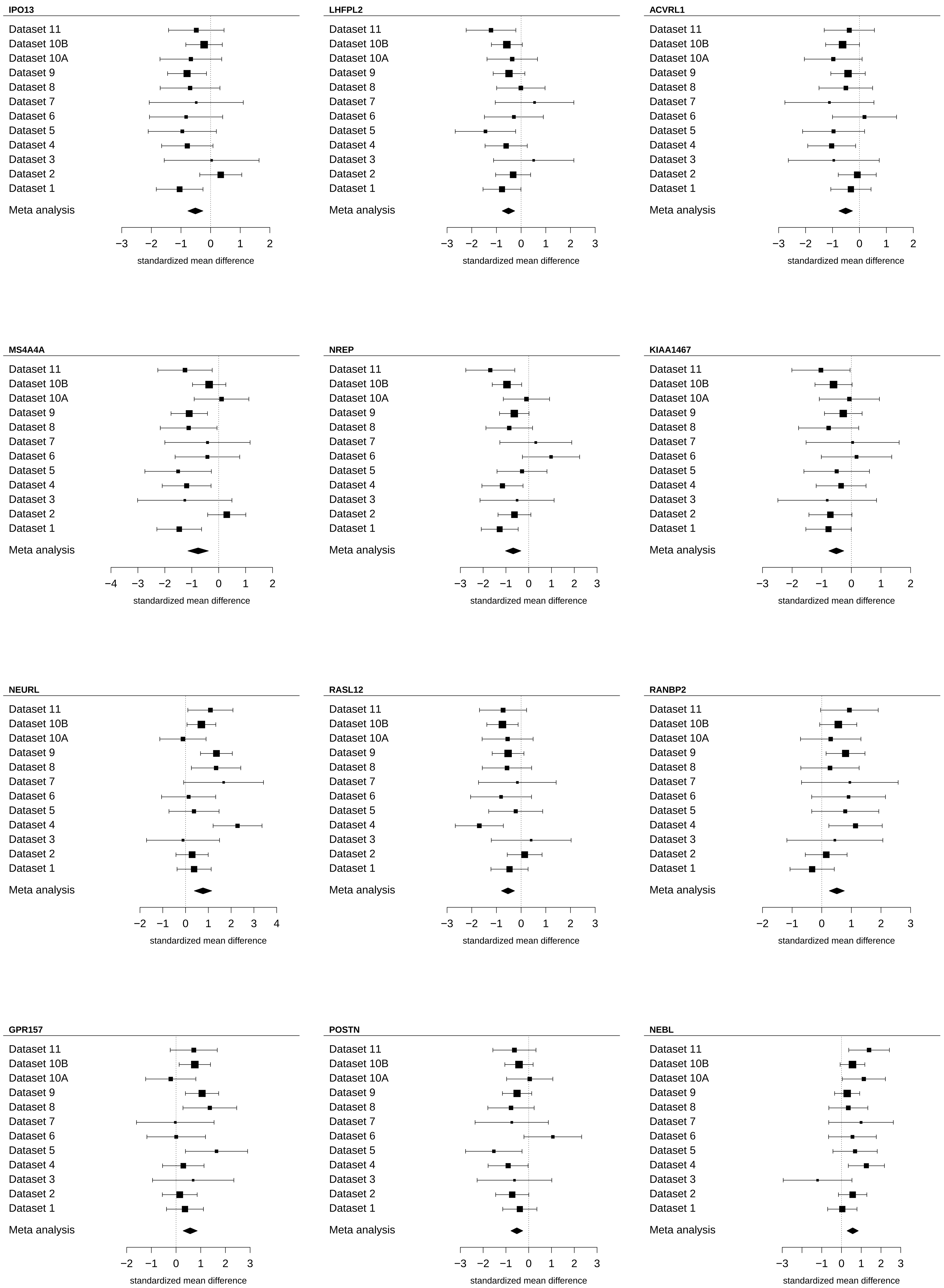
IPO13
LHFPL2
ACVRL1
MS4A4A
NREP
KIAA1467
NEURL
RASL12
RANBP2
GPR157
POSTN
NEBL
Dataset 11
Dataset 10B
Dataset 10A
Dataset 9
Dataset 8
Dataset 7
Dataset 6
Dataset 5
Dataset 4
Dataset 3
Dataset 2
Dataset 1
Meta analysis
standardized mean difference

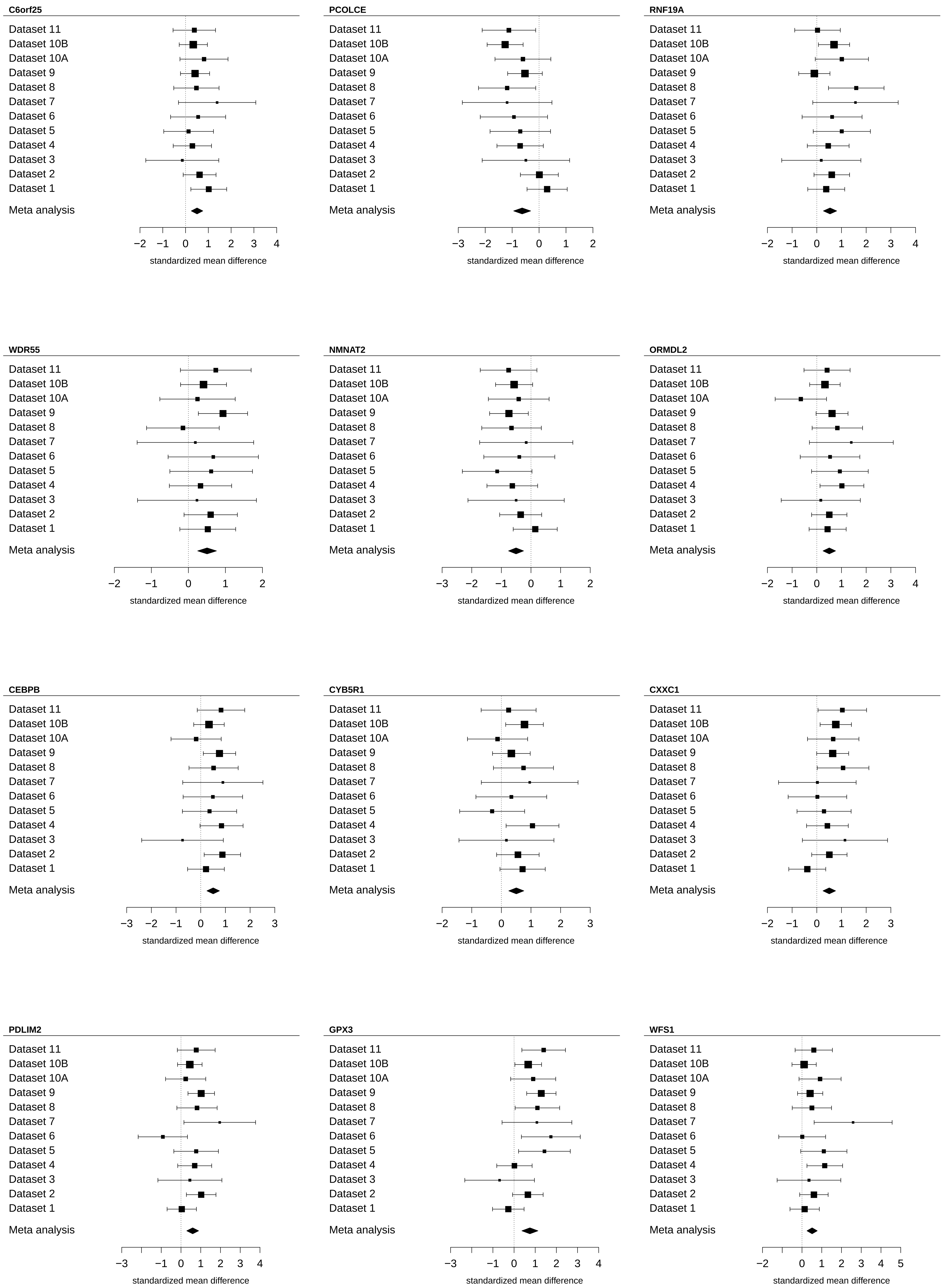
C6orf25
PCOLCE
RNF19A
WDR55
NMNAT2
ORMDL2
CEBPB
CYB5R1
CXXC1
PDLIM2
GPX3
WFS1
Dataset 11
Dataset 10B
Dataset 10A
Dataset 9
Dataset 8
Dataset 7
Dataset 6
Dataset 5
Dataset 4
Dataset 3
Dataset 2
Dataset 1
Meta analysis
standardized mean difference

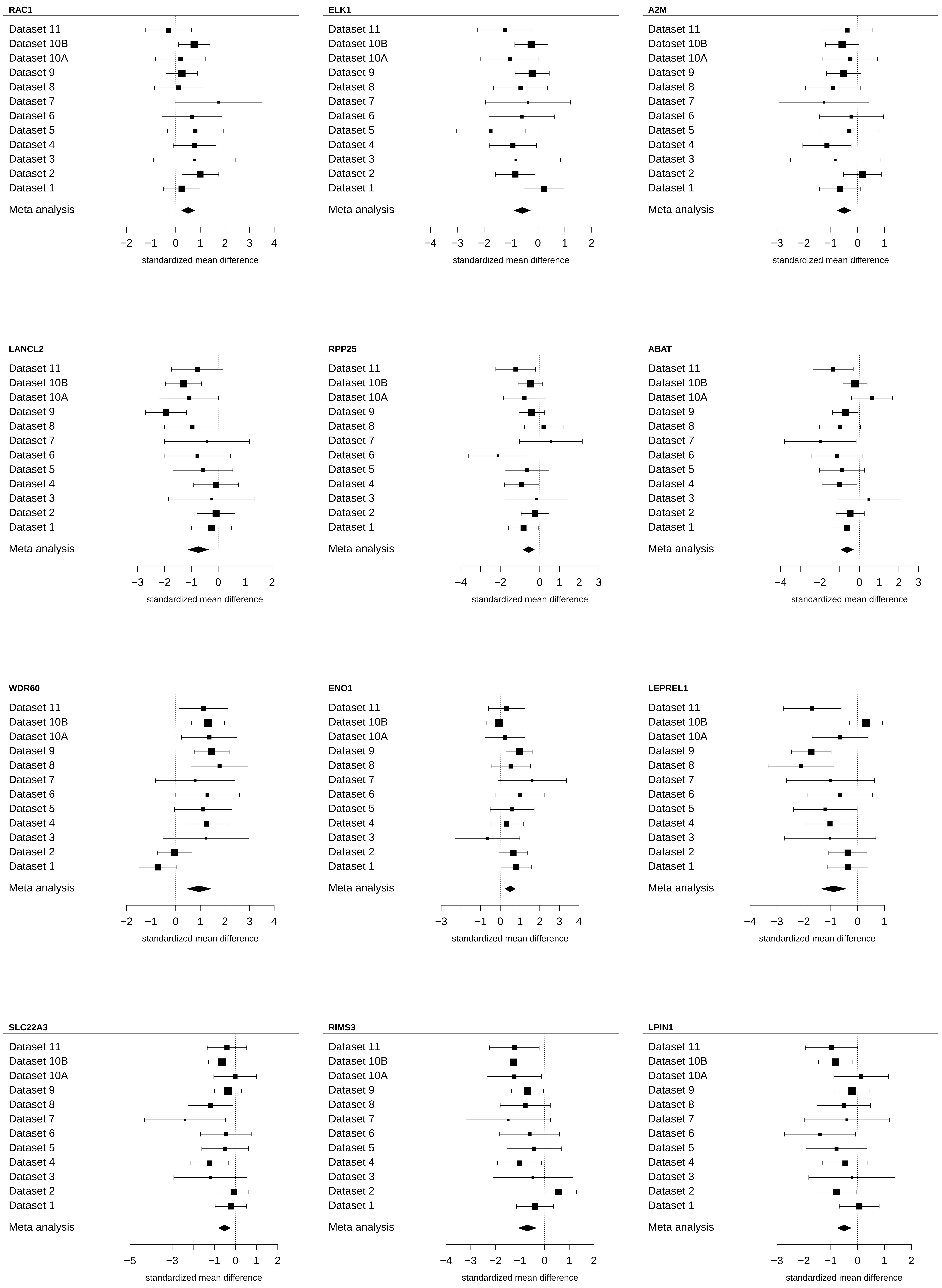
RAC1
ELK1
A2M
LANCL2
RPP25
ABAT
WDR60
ENO1
LEPREL1
SLC22A3
RIMS3
LPIN1
Dataset 11
Dataset 10B
Dataset 10A
Dataset 9
Dataset 8
Dataset 7
Dataset 6
Dataset 5
Dataset 4
Dataset 3
Dataset 2
Dataset 1
Meta analysis
−2 −1 0 1 2 3 4
−4 −3 −2 −1 0 1 2
−3 −2 −1 0 1
−3 −2 −1 0 1 2
−4 −2 0 1 2 3
−4 −2 0 1 2 3
−2 −1 0 1 2 3 4
−3 −1 0 1 2 3 4
−4 −3 −2 −1 0 1
−5 −3 −1 0 1 2
−4 −3 −2 −1 0 1 2
−3 −2 −1 0 1 2
standardized mean difference

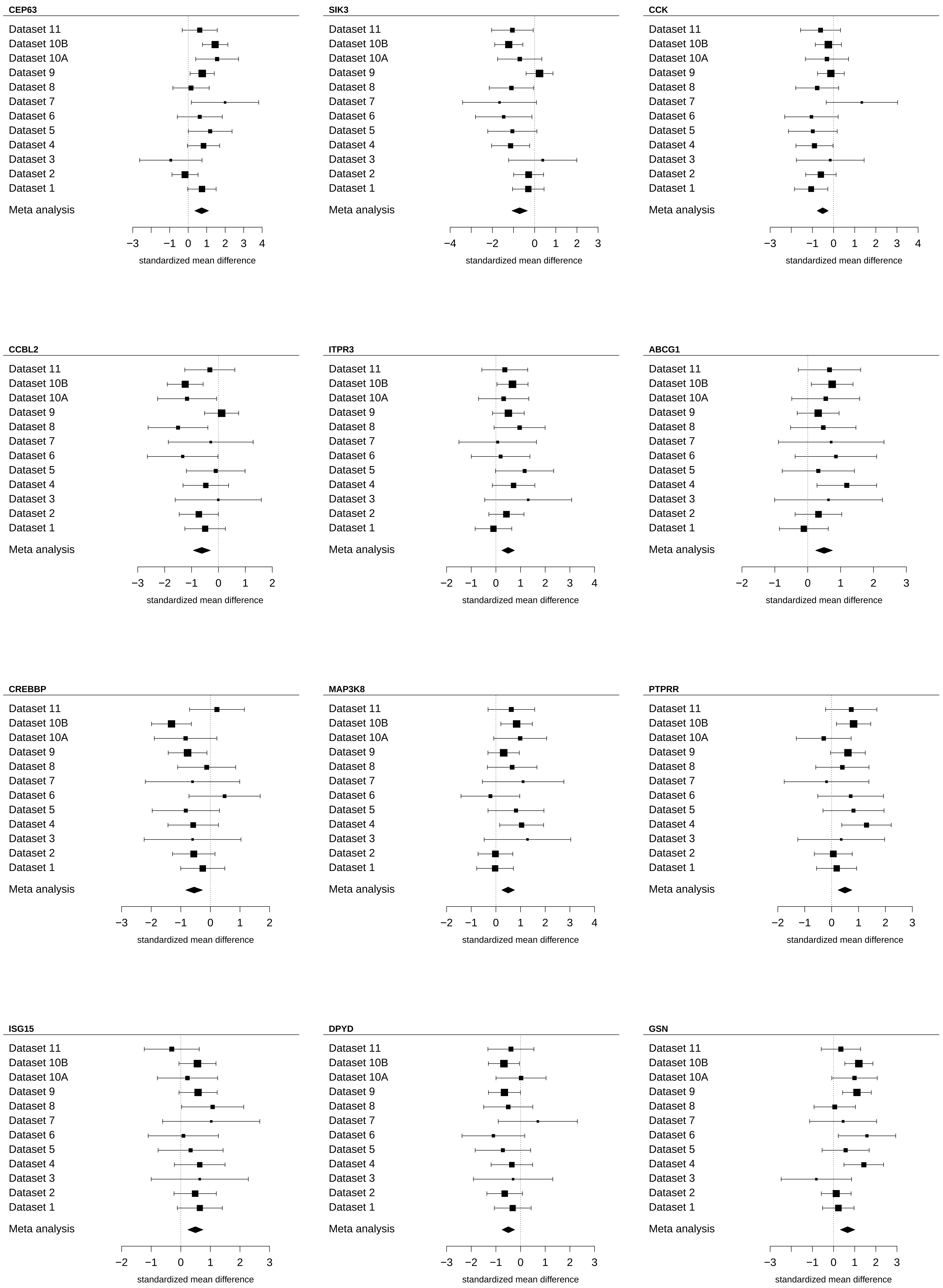
CEP63
Dataset 11
Dataset 10B
Dataset 10A
Dataset 9
Dataset 8
Dataset 7
Dataset 6
Dataset 5
Dataset 4
Dataset 3
Dataset 2
Dataset 1
Meta analysis
−3 −1 0 1 2 3 4
standardized mean difference
SIK3
Dataset 11
Dataset 10B
Dataset 10A
Dataset 9
Dataset 8
Dataset 7
Dataset 6
Dataset 5
Dataset 4
Dataset 3
Dataset 2
Dataset 1
Meta analysis
−4 −2 0 1 2 3
standardized mean difference
CCK
Dataset 11
Dataset 10B
Dataset 10A
Dataset 9
Dataset 8
Dataset 7
Dataset 6
Dataset 5
Dataset 4
Dataset 3
Dataset 2
Dataset 1
Meta analysis
−3 −1 0 1 2 3 4
standardized mean difference
CCBL2
Dataset 11
Dataset 10B
Dataset 10A
Dataset 9
Dataset 8
Dataset 7
Dataset 6
Dataset 5
Dataset 4
Dataset 3
Dataset 2
Dataset 1
Meta analysis
−3 −2 −1 0 1 2
standardized mean difference
ITPR3
Dataset 11
Dataset 10B
Dataset 10A
Dataset 9
Dataset 8
Dataset 7
Dataset 6
Dataset 5
Dataset 4
Dataset 3
Dataset 2
Dataset 1
Meta analysis
−2 −1 0 1 2 3 4
standardized mean difference
ABCG1
Dataset 11
Dataset 10B
Dataset 10A
Dataset 9
Dataset 8
Dataset 7
Dataset 6
Dataset 5
Dataset 4
Dataset 3
Dataset 2
Dataset 1
Meta analysis
−2 −1 0 1 2 3
standardized mean difference
CREBBP
Dataset 11
Dataset 10B
Dataset 10A
Dataset 9
Dataset 8
Dataset 7
Dataset 6
Dataset 5
Dataset 4
Dataset 3
Dataset 2
Dataset 1
Meta analysis
−3 −2 −1 0 1 2
standardized mean difference
MAP3K8
Dataset 11
Dataset 10B
Dataset 10A
Dataset 9
Dataset 8
Dataset 7
Dataset 6
Dataset 5
Dataset 4
Dataset 3
Dataset 2
Dataset 1
Meta analysis
−2 −1 0 1 2 3 4
standardized mean difference
PTPRR
Dataset 11
Dataset 10B
Dataset 10A
Dataset 9
Dataset 8
Dataset 7
Dataset 6
Dataset 5
Dataset 4
Dataset 3
Dataset 2
Dataset 1
Meta analysis
−2 −1 0 1 2 3
standardized mean difference
ISG15
Dataset 11
Dataset 10B
Dataset 10A
Dataset 9
Dataset 8
Dataset 7
Dataset 6
Dataset 5
Dataset 4
Dataset 3
Dataset 2
Dataset 1
Meta analysis
−2 −1 0 1 2 3
standardized mean difference
DPYD
Dataset 11
Dataset 10B
Dataset 10A
Dataset 9
Dataset 8
Dataset 7
Dataset 6
Dataset 5
Dataset 4
Dataset 3
Dataset 2
Dataset 1
Meta analysis
−3 −2 −1 0 1 2 3
standardized mean difference
GSN
Dataset 11
Dataset 10B
Dataset 10A
Dataset 9
Dataset 8
Dataset 7
Dataset 6
Dataset 5
Dataset 4
Dataset 3
Dataset 2
Dataset 1
Meta analysis
−3 −1 0 1 2 3
standardized mean difference

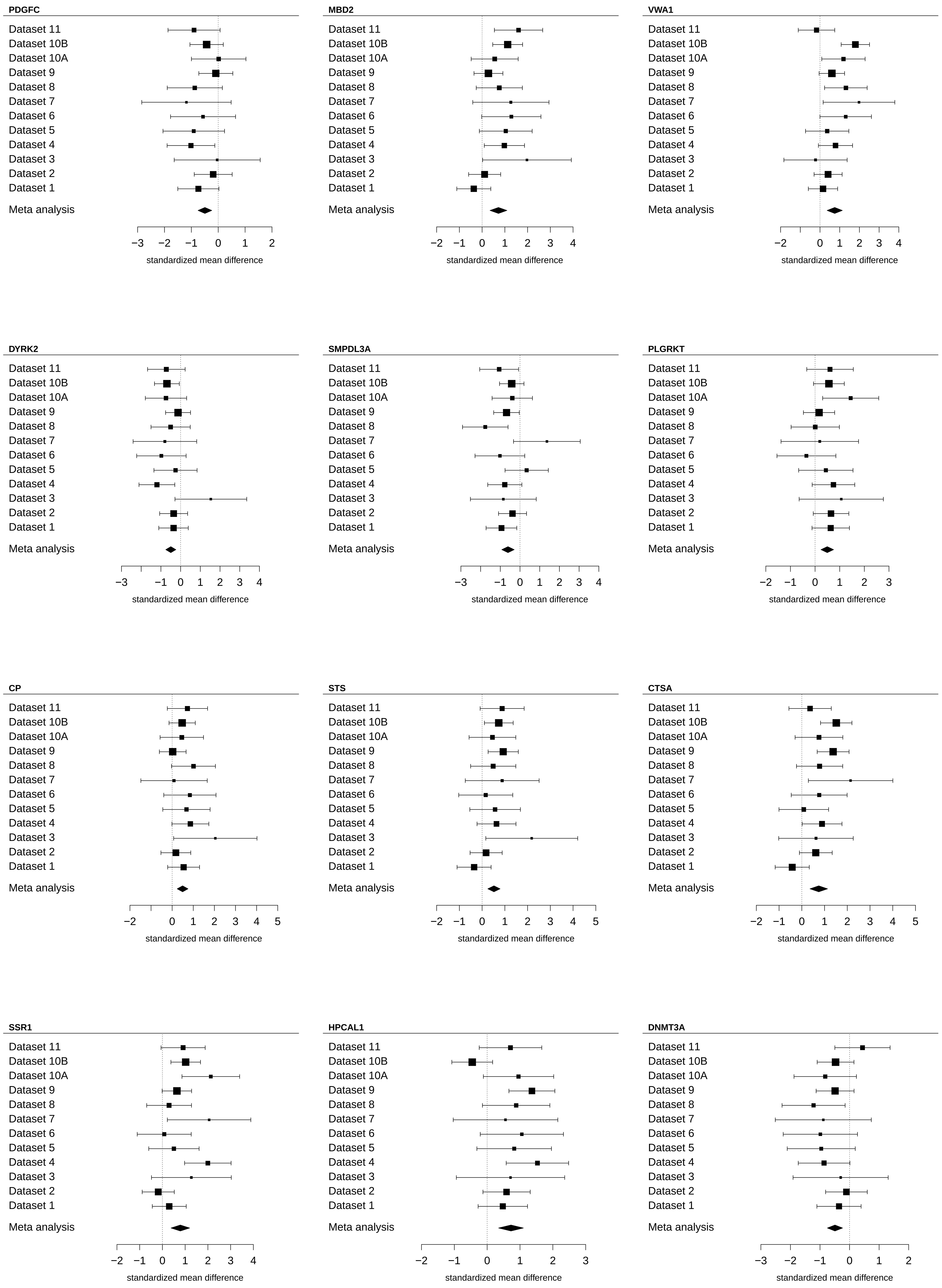
PDGFC
Dataset 11
Dataset 10B
Dataset 10A
Dataset 9
Dataset 8
Dataset 7
Dataset 6
Dataset 5
Dataset 4
Dataset 3
Dataset 2
Dataset 1
Meta analysis
−3 −2 −1 0 1 2
standardized mean difference
MBD2
Dataset 11
Dataset 10B
Dataset 10A
Dataset 9
Dataset 8
Dataset 7
Dataset 6
Dataset 5
Dataset 4
Dataset 3
Dataset 2
Dataset 1
Meta analysis
−2 −1 0 1 2 3 4
standardized mean difference
VWA1
Dataset 11
Dataset 10B
Dataset 10A
Dataset 9
Dataset 8
Dataset 7
Dataset 6
Dataset 5
Dataset 4
Dataset 3
Dataset 2
Dataset 1
Meta analysis
−2 0 1 2 3 4
standardized mean difference
DYRK2
Dataset 11
Dataset 10B
Dataset 10A
Dataset 9
Dataset 8
Dataset 7
Dataset 6
Dataset 5
Dataset 4
Dataset 3
Dataset 2
Dataset 1
Meta analysis
−3 −1 0 1 2 3 4
standardized mean difference
SMPDL3A
Dataset 11
Dataset 10B
Dataset 10A
Dataset 9
Dataset 8
Dataset 7
Dataset 6
Dataset 5
Dataset 4
Dataset 3
Dataset 2
Dataset 1
Meta analysis
−3 −1 0 1 2 3 4
standardized mean difference
PLGRKT
Dataset 11
Dataset 10B
Dataset 10A
Dataset 9
Dataset 8
Dataset 7
Dataset 6
Dataset 5
Dataset 4
Dataset 3
Dataset 2
Dataset 1
Meta analysis
−2 −1 0 1 2 3
standardized mean difference
CP
Dataset 11
Dataset 10B
Dataset 10A
Dataset 9
Dataset 8
Dataset 7
Dataset 6
Dataset 5
Dataset 4
Dataset 3
Dataset 2
Dataset 1
Meta analysis
−2 0 1 2 3 4 5
standardized mean difference
STS
Dataset 11
Dataset 10B
Dataset 10A
Dataset 9
Dataset 8
Dataset 7
Dataset 6
Dataset 5
Dataset 4
Dataset 3
Dataset 2
Dataset 1
Meta analysis
−2 −1 0 1 2 3 4 5
standardized mean difference
CTSA
Dataset 11
Dataset 10B
Dataset 10A
Dataset 9
Dataset 8
Dataset 7
Dataset 6
Dataset 5
Dataset 4
Dataset 3
Dataset 2
Dataset 1
Meta analysis
−2 −1 0 1 2 3 4 5
standardized mean difference
SSR1
Dataset 11
Dataset 10B
Dataset 10A
Dataset 9
Dataset 8
Dataset 7
Dataset 6
Dataset 5
Dataset 4
Dataset 3
Dataset 2
Dataset 1
Meta analysis
−2 −1 0 1 2 3 4
standardized mean difference
HPCAL1
Dataset 11
Dataset 10B
Dataset 10A
Dataset 9
Dataset 8
Dataset 7
Dataset 6
Dataset 5
Dataset 4
Dataset 3
Dataset 2
Dataset 1
Meta analysis
−2 −1 0 1 2 3
standardized mean difference
DNMT3A
Dataset 11
Dataset 10B
Dataset 10A
Dataset 9
Dataset 8
Dataset 7
Dataset 6
Dataset 5
Dataset 4
Dataset 3
Dataset 2
Dataset 1
Meta analysis
−3 −2 −1 0 1 2
standardized mean difference

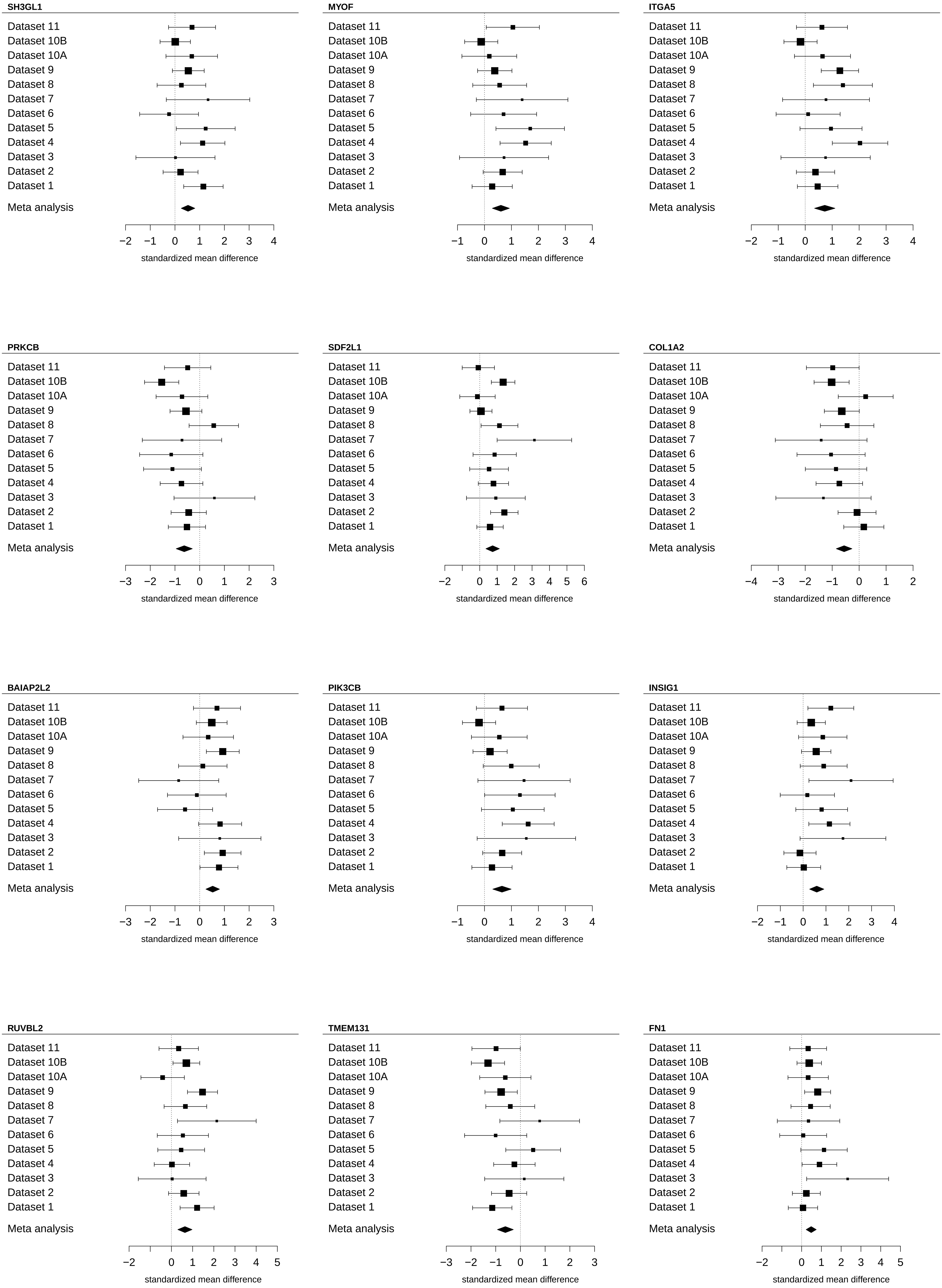
SH3GL1
MYOF
ITGA5
PRKCB
SDF2L1
COL1A2
BAIAP2L2
PIK3CB
INSIG1
RUVBL2
TMEM131
FN1
Dataset 11
Dataset 10B
Dataset 10A
Dataset 9
Dataset 8
Dataset 7
Dataset 6
Dataset 5
Dataset 4
Dataset 3
Dataset 2
Dataset 1
Meta analysis
standardized mean difference

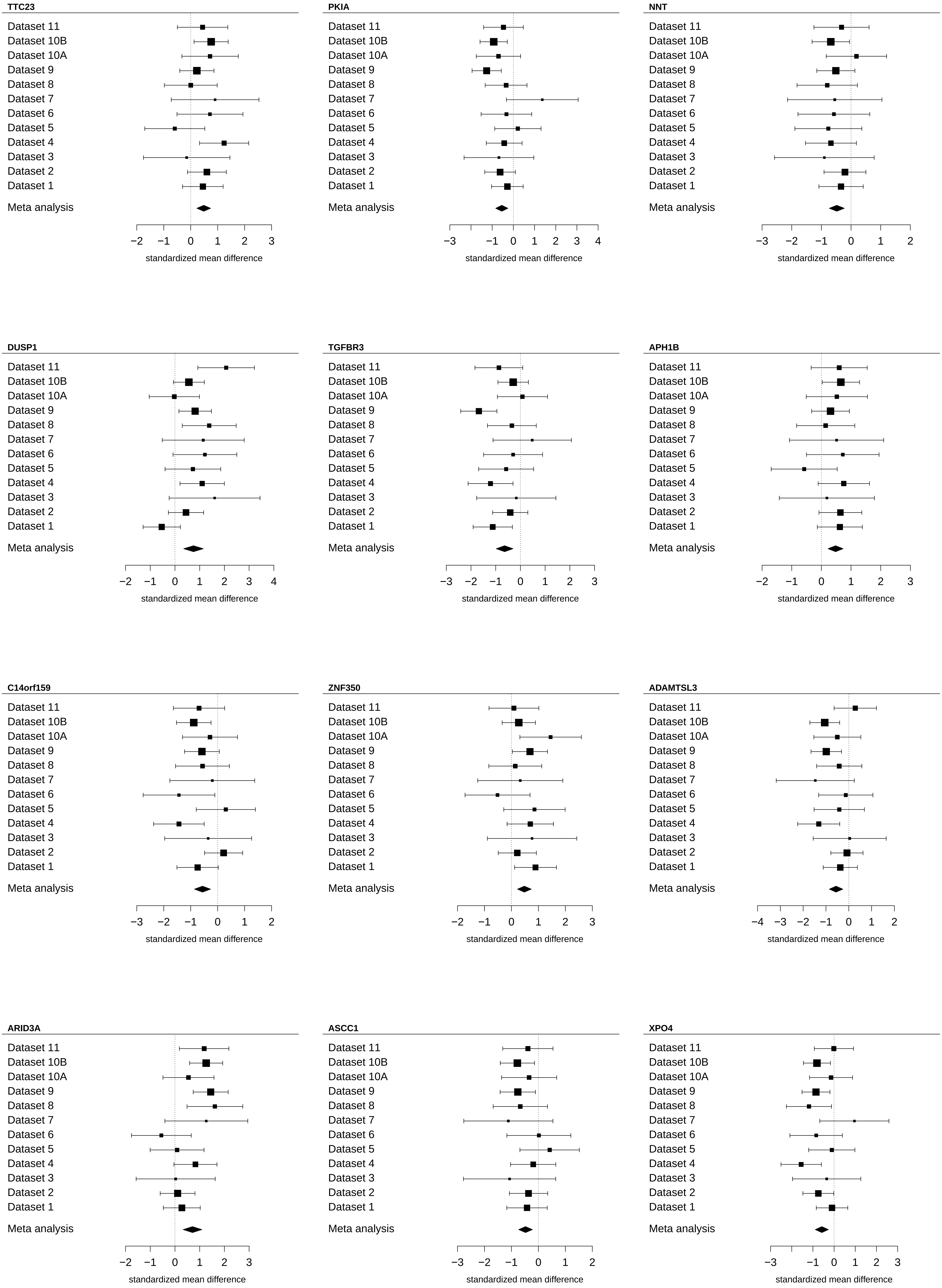
TTC23
Dataset 11
Dataset 10B
Dataset 10A
Dataset 9
Dataset 8
Dataset 7
Dataset 6
Dataset 5
Dataset 4
Dataset 3
Dataset 2
Dataset 1
Meta analysis
−2 −1 0 1 2 3
standardized mean difference
PKIA
Dataset 11
Dataset 10B
Dataset 10A
Dataset 9
Dataset 8
Dataset 7
Dataset 6
Dataset 5
Dataset 4
Dataset 3
Dataset 2
Dataset 1
Meta analysis
−3 −1 0 1 2 3 4
standardized mean difference
NNT
Dataset 11
Dataset 10B
Dataset 10A
Dataset 9
Dataset 8
Dataset 7
Dataset 6
Dataset 5
Dataset 4
Dataset 3
Dataset 2
Dataset 1
Meta analysis
−3 −2 −1 0 1 2
standardized mean difference
DUSP1
Dataset 11
Dataset 10B
Dataset 10A
Dataset 9
Dataset 8
Dataset 7
Dataset 6
Dataset 5
Dataset 4
Dataset 3
Dataset 2
Dataset 1
Meta analysis
−2 −1 0 1 2 3 4
standardized mean difference
TGFBR3
Dataset 11
Dataset 10B
Dataset 10A
Dataset 9
Dataset 8
Dataset 7
Dataset 6
Dataset 5
Dataset 4
Dataset 3
Dataset 2
Dataset 1
Meta analysis
−3 −2 −1 0 1 2 3
standardized mean difference
APH1B
Dataset 11
Dataset 10B
Dataset 10A
Dataset 9
Dataset 8
Dataset 7
Dataset 6
Dataset 5
Dataset 4
Dataset 3
Dataset 2
Dataset 1
Meta analysis
−2 −1 0 1 2 3
standardized mean difference
C14orf159
Dataset 11
Dataset 10B
Dataset 10A
Dataset 9
Dataset 8
Dataset 7
Dataset 6
Dataset 5
Dataset 4
Dataset 3
Dataset 2
Dataset 1
Meta analysis
−3 −2 −1 0 1 2
standardized mean difference
ZNF350
Dataset 11
Dataset 10B
Dataset 10A
Dataset 9
Dataset 8
Dataset 7
Dataset 6
Dataset 5
Dataset 4
Dataset 3
Dataset 2
Dataset 1
Meta analysis
−2 −1 0 1 2 3
standardized mean difference
ADAMTSL3
Dataset 11
Dataset 10B
Dataset 10A
Dataset 9
Dataset 8
Dataset 7
Dataset 6
Dataset 5
Dataset 4
Dataset 3
Dataset 2
Dataset 1
Meta analysis
−4 −3 −2 −1 0 1 2
standardized mean difference
ARID3A
Dataset 11
Dataset 10B
Dataset 10A
Dataset 9
Dataset 8
Dataset 7
Dataset 6
Dataset 5
Dataset 4
Dataset 3
Dataset 2
Dataset 1
Meta analysis
−2 −1 0 1 2 3
standardized mean difference
ASCC1
Dataset 11
Dataset 10B
Dataset 10A
Dataset 9
Dataset 8
Dataset 7
Dataset 6
Dataset 5
Dataset 4
Dataset 3
Dataset 2
Dataset 1
Meta analysis
−3 −2 −1 0 1 2
standardized mean difference
XPO4
Dataset 11
Dataset 10B
Dataset 10A
Dataset 9
Dataset 8
Dataset 7
Dataset 6
Dataset 5
Dataset 4
Dataset 3
Dataset 2
Dataset 1
Meta analysis
−3 −1 0 1 2 3
standardized mean difference

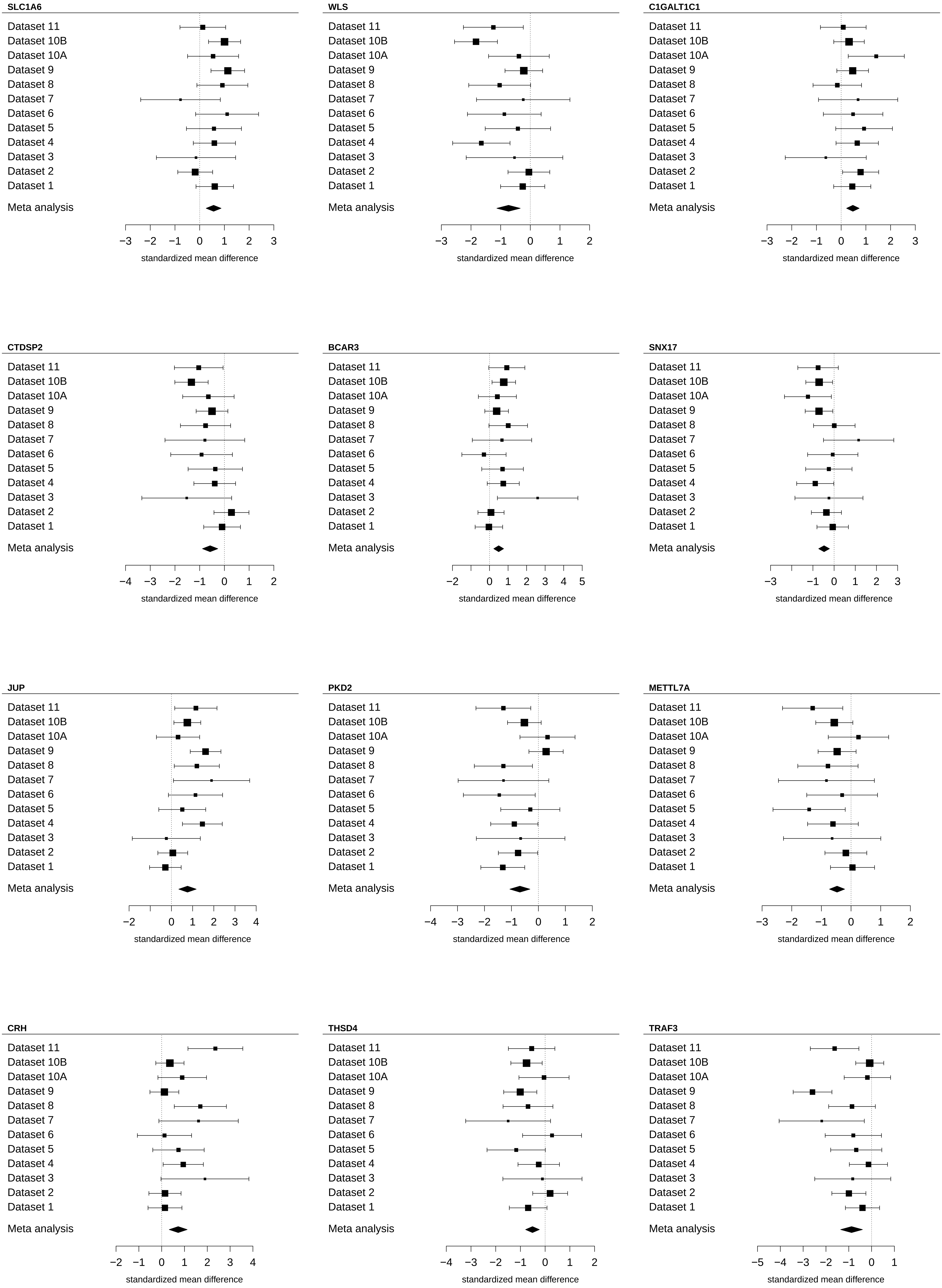
SLC1A6
WLS
C1GALT1C1
CTDSP2
BCAR3
SNX17
JUP
PKD2
METTL7A
CRH
THSD4
TRAF3
Dataset 11
Dataset 10B
Dataset 10A
Dataset 9
Dataset 8
Dataset 7
Dataset 6
Dataset 5
Dataset 4
Dataset 3
Dataset 2
Dataset 1
Meta analysis
standardized mean difference

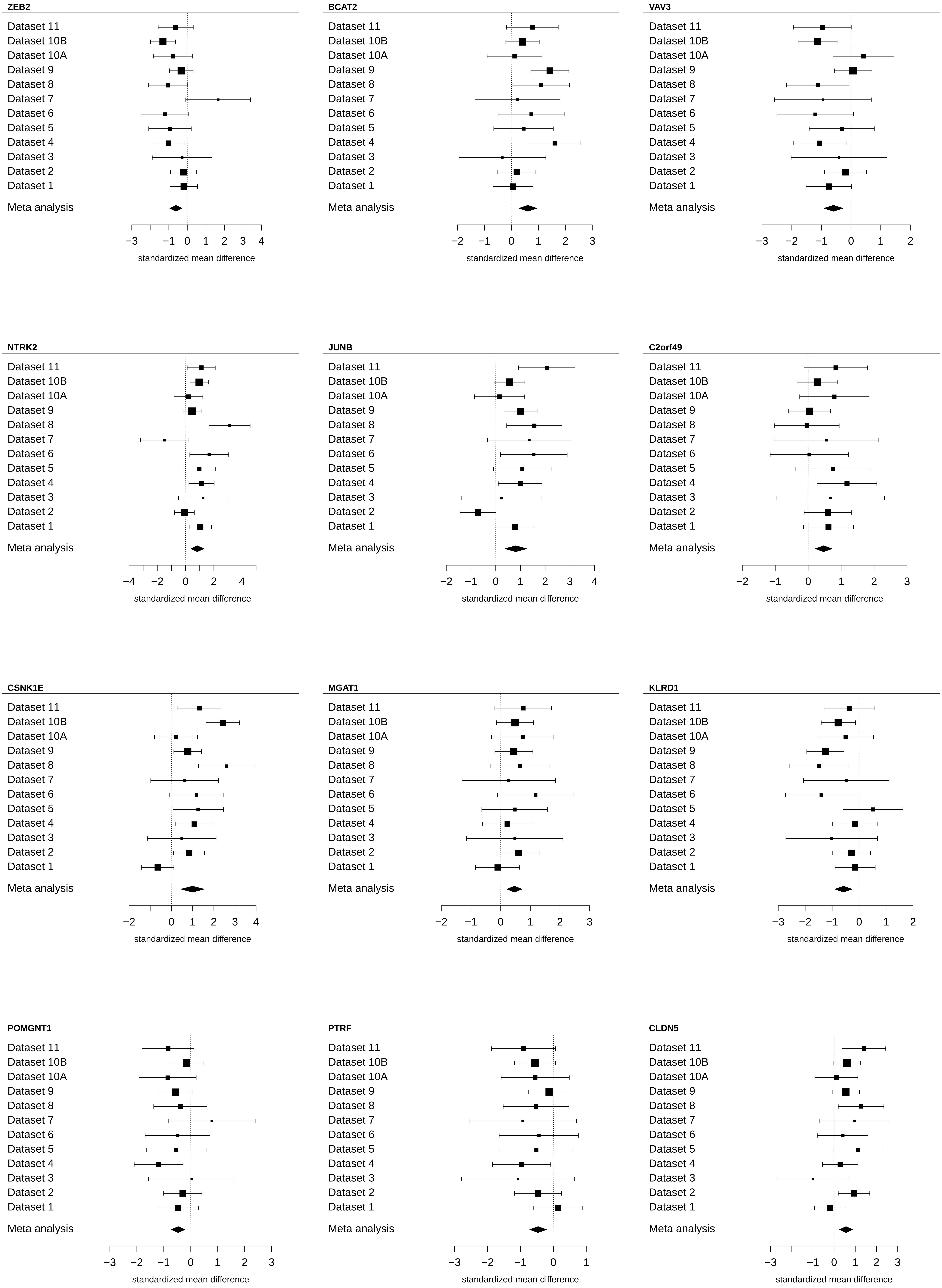
ZEB2
BCAT2
VAV3
NTRK2
JUNB
C2orf49
CSNK1E
MGAT1
KLRD1
POMGNT1
PTRF
CLDN5
Dataset 11
Dataset 10B
Dataset 10A
Dataset 9
Dataset 8
Dataset 7
Dataset 6
Dataset 5
Dataset 4
Dataset 3
Dataset 2
Dataset 1
Meta analysis
standardized mean difference

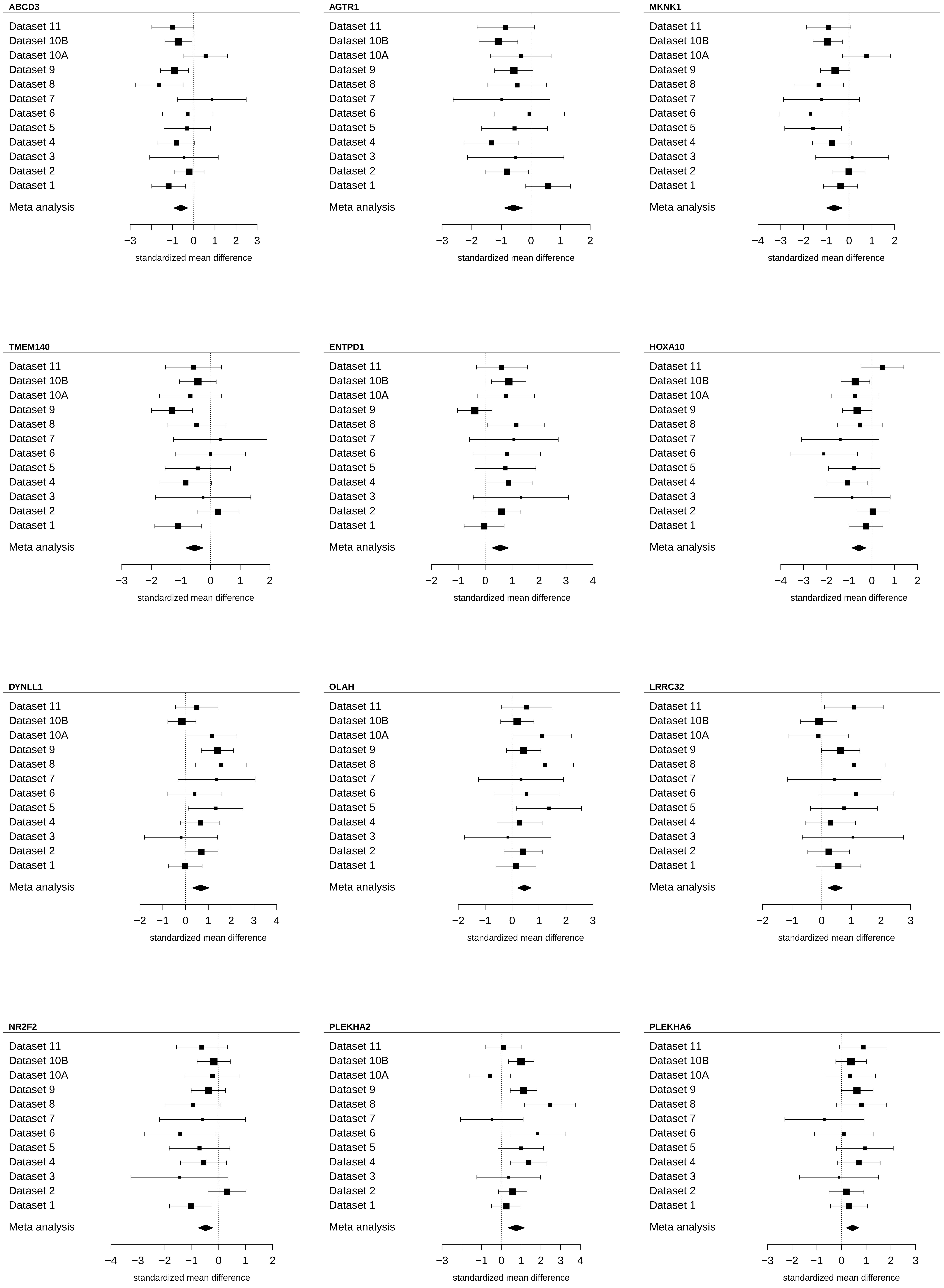
ABCD3
Dataset 11
Dataset 10B
Dataset 10A
Dataset 9
Dataset 8
Dataset 7
Dataset 6
Dataset 5
Dataset 4
Dataset 3
Dataset 2
Dataset 1
Meta analysis
−3 −1 0 1 2 3
standardized mean difference
AGTR1
Dataset 11
Dataset 10B
Dataset 10A
Dataset 9
Dataset 8
Dataset 7
Dataset 6
Dataset 5
Dataset 4
Dataset 3
Dataset 2
Dataset 1
Meta analysis
−3 −2 −1 0 1 2
standardized mean difference
MKNK1
Dataset 11
Dataset 10B
Dataset 10A
Dataset 9
Dataset 8
Dataset 7
Dataset 6
Dataset 5
Dataset 4
Dataset 3
Dataset 2
Dataset 1
Meta analysis
−4 −3 −2 −1 0 1 2
standardized mean difference
TMEM140
Dataset 11
Dataset 10B
Dataset 10A
Dataset 9
Dataset 8
Dataset 7
Dataset 6
Dataset 5
Dataset 4
Dataset 3
Dataset 2
Dataset 1
Meta analysis
−3 −2 −1 0 1 2
standardized mean difference
ENTPD1
Dataset 11
Dataset 10B
Dataset 10A
Dataset 9
Dataset 8
Dataset 7
Dataset 6
Dataset 5
Dataset 4
Dataset 3
Dataset 2
Dataset 1
Meta analysis
−2 −1 0 1 2 3 4
standardized mean difference
HOXA10
Dataset 11
Dataset 10B
Dataset 10A
Dataset 9
Dataset 8
Dataset 7
Dataset 6
Dataset 5
Dataset 4
Dataset 3
Dataset 2
Dataset 1
Meta analysis
−4 −3 −2 −1 0 1 2
standardized mean difference
DYNLL1
Dataset 11
Dataset 10B
Dataset 10A
Dataset 9
Dataset 8
Dataset 7
Dataset 6
Dataset 5
Dataset 4
Dataset 3
Dataset 2
Dataset 1
Meta analysis
−2 −1 0 1 2 3 4
standardized mean difference
OLAH
Dataset 11
Dataset 10B
Dataset 10A
Dataset 9
Dataset 8
Dataset 7
Dataset 6
Dataset 5
Dataset 4
Dataset 3
Dataset 2
Dataset 1
Meta analysis
−2 −1 0 1 2 3
standardized mean difference
LRRC32
Dataset 11
Dataset 10B
Dataset 10A
Dataset 9
Dataset 8
Dataset 7
Dataset 6
Dataset 5
Dataset 4
Dataset 3
Dataset 2
Dataset 1
Meta analysis
−2 −1 0 1 2 3
standardized mean difference
NR2F2
Dataset 11
Dataset 10B
Dataset 10A
Dataset 9
Dataset 8
Dataset 7
Dataset 6
Dataset 5
Dataset 4
Dataset 3
Dataset 2
Dataset 1
Meta analysis
−4 −3 −2 −1 0 1 2
standardized mean difference
PLEKHA2
Dataset 11
Dataset 10B
Dataset 10A
Dataset 9
Dataset 8
Dataset 7
Dataset 6
Dataset 5
Dataset 4
Dataset 3
Dataset 2
Dataset 1
Meta analysis
−3 −1 0 1 2 3 4
standardized mean difference
PLEKHA6
Dataset 11
Dataset 10B
Dataset 10A
Dataset 9
Dataset 8
Dataset 7
Dataset 6
Dataset 5
Dataset 4
Dataset 3
Dataset 2
Dataset 1
Meta analysis
−3 −2 −1 0 1 2 3
standardized mean difference

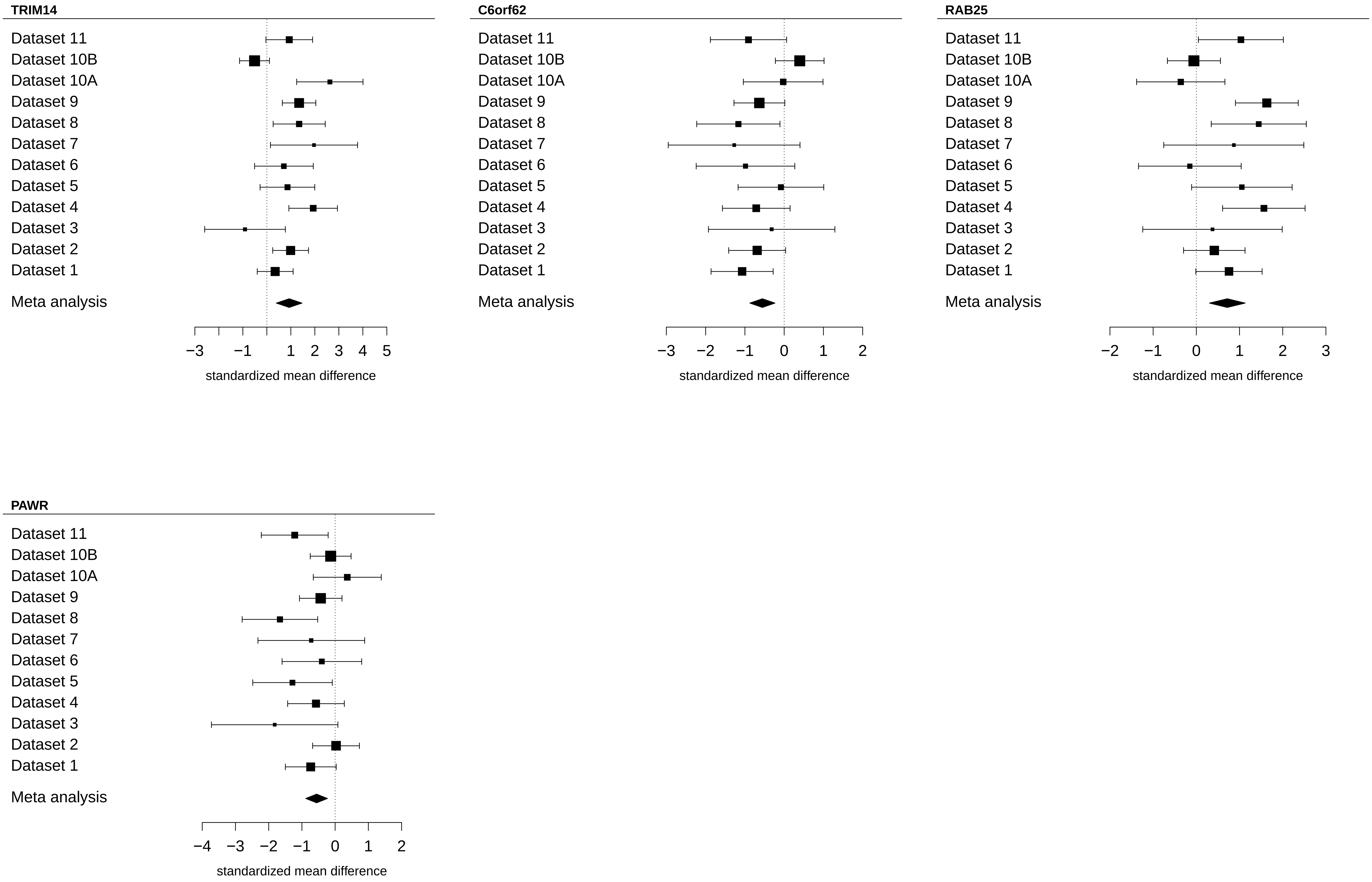

S1 Figure. **Forest plots of meta-signature genes.**

Forest plots of the 388 differentially expressed genes in the preeclamptic placenta. Squares are proportional to weights used in the meta-analysis, and the lines represent the 95% confidence interval. The diamond represents the standardized mean difference (log2 scale).
