# Supplementary material for: Meta-Analysis of Placental Transcriptome Data Identifies a Novel Molecular Pathway Related to Preeclampsia
Source: PLoS One. 2015 Jul 14;10(7):e0132468. doi: 10.1371/journal.pone.0132468 (PMC4501668; doi:10.1371/journal.pone.0132468)
Supplement: S2 Fig — All 388 genes from the meta‐signature were used as input for STRING analysis and a network was built based on high confidence (0.8) evidence from experimental protein‐protein interaction (blue lines) and curated (purple lines) databases. Proteins are indicated by nodes labeled with the encoding gene symbol. Two genes (CEBPA and SEPP1) are not present in the used STRING version 9.1. The network is enriched in interactions (p = 0.007) using the intersection of 8,612 genes present on all platforms as background. Additional cluster analysis was used to color the nodes of the interaction networks. Yellow nodes represent proteins that have no protein‐protein interaction at this confidence setting. (PDF) [file pone.0132468.s002.pdf]

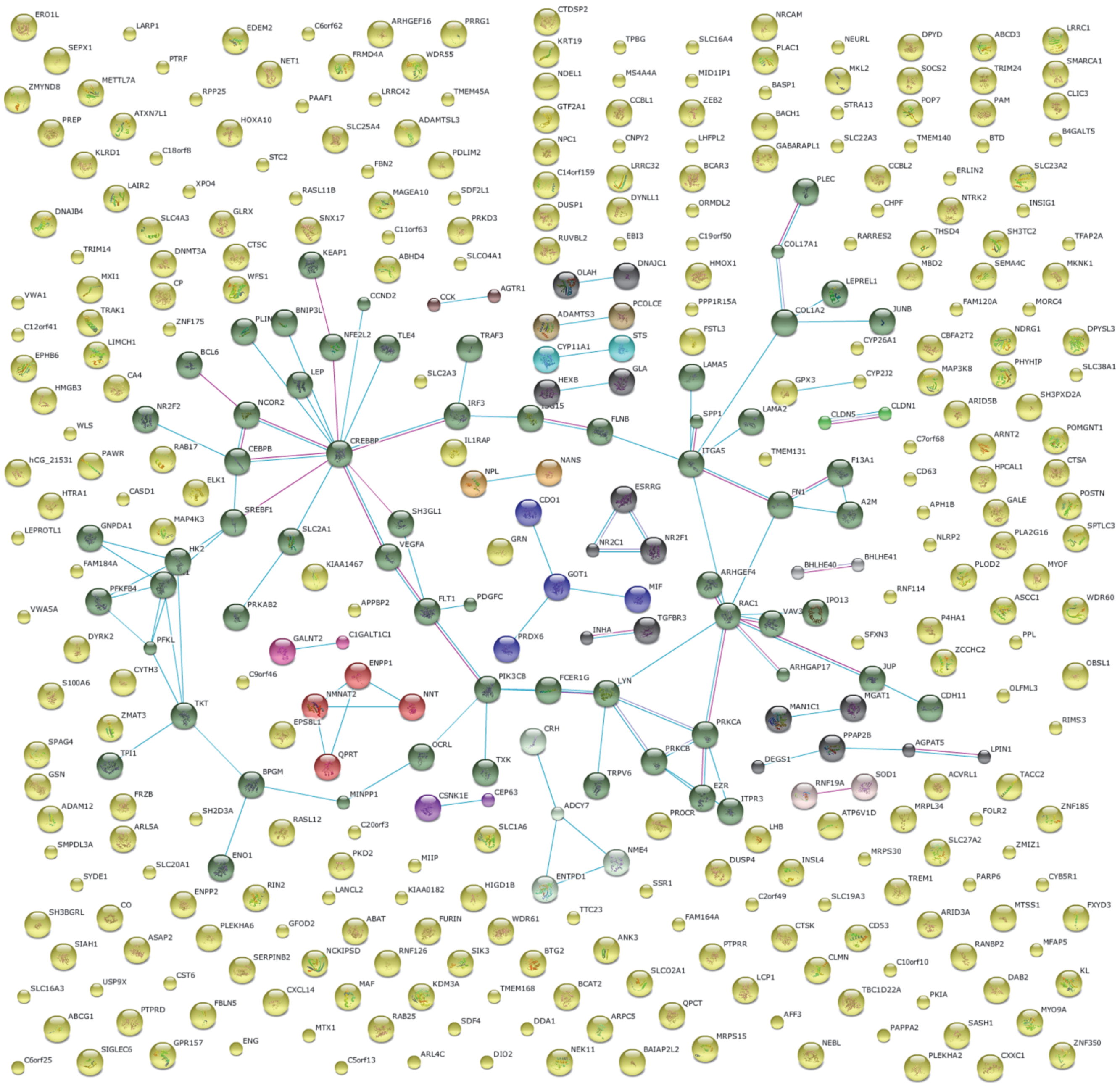

S2 Figure. STRING protein interaction analysis

All 388 genes from the meta-signature were used as input for STRING analysis and a network was built based on high confidence (0.8) evidence from experimental protein-protein interaction (blue lines) and curated (purple lines) databases. Proteins are indicated by nodes labeled with the encoding gene symbol. Two genes (CEBPA and SEPP1) are not present in the used STRING version 9.1. The network is enriched in interactions (p=0.007) using the intersection of 8,612 genes present on all platforms as background. Additional cluster analysis was used to color the nodes of the interaction networks. Yellow nodes represent proteins that have no protein-protein interaction at this confidence setting.
