# Supplementary material for: Meta-Analysis of Placental Transcriptome Data Identifies a Novel Molecular Pathway Related to Preeclampsia
Source: PLoS One. 2015 Jul 14;10(7):e0132468. doi: 10.1371/journal.pone.0132468 (PMC4501668; doi:10.1371/journal.pone.0132468)
Supplement: S1 Text — (DOCX) [file pone.0132468.s007.docx]

**Supplementary Methods**

We followed the guidelines for conducting a meta-analysis of microarray datasets as described in Ramasamy et al ^1^. Analyses were carried out with packages from Bioconductor in the statistical software package R (version 3.0.2).

*Pre-processing and probe annotation.*

If available, original data was retrieved from GEO and subsequently pre-processed using state-of-the art methods for each of the platforms included. For three datasets (2,7,9) only already pre-processed data could be obtained from the authors. Probes were mapped to their corresponding Entrez gene ID using the Bioconductor annotation packages for the corresponding platform, if available. Alternatively, annotation files supplied by the authors were used. See Table ‘Characteristics of data analysis’ for details.

*Quality control.*

The R package arrayQualityMetrics (version 3.18.0) was used to assess the data quality of the individual samples for each dataset. For each dataset with raw data available we used a two-step procedure. 1) The raw data was analyzed using arrayQualityMetrics. If a sample was detected as an outlier according to three or more of the standardly computed criteria, it was considered to be of low quality and subsequently discarded. 2) The remaining samples were normalized using *rma* (package affy, version 1.40.0) or *neqc* (package limma, version 3.18.13) or a similar procedure; see Table ‘Characteristics of data analysis’. The normalized samples were then analyzed again using arrayQualityMetrics. This time a sample was only considered to be of low quality and discarded if it was detected as an outlier according to one of the criteria “distances between arrays” or “MA plots” or, or for dual channel arrays, “boxplots”. For all datasets with only pre-processed data available we used the same arrayQualityMetrics criteria as in step 2.

*Meta-analysis.*

The meta-analysis was performed by estimating the effect sizes and combining them using an inverse-variance random-effects model^2^ using the R package GeneMeta (version 1.34.0):

- Step 1. For each of the eight platforms the probes that did not have an Entrez gene ID were discarded (Table 1).
- Step 2. The meta-analysis was conducted using only those probes that corresponded to one of the 8,612 Entrez gene IDs present on all platforms.
- Step 3. For each dataset we computed the effect size (Cohen’s d, scaled using the Hedges and Olkin correction^3^) for all probes that were selected in Step 2. Cohen’s d is the difference in two group means standardized by its pooled standard deviation.
- Step 4. For each dataset, probes were kept only if they were unique to a specific Entrez Gene ID. In case an Entrez Gene ID was represented by multiple probes, we first checked whether all effect sizes as calculated in Step 3, were either positive or negative. If so, the probe with the largest (absolute) effect size was selected. If not, the probe with the largest variance was selected. This resulted in a representative probe for each of the 8,612 genes for each dataset.
- Step 5. Dataset-specific effect sizes were then combined using random effects inverse-variance weighting for each gene to calculate the pooled effect size and the corresponding standard error. Z-statistics (and their corresponding p-values) were calculated by taking the ratio of the pooled standardized mean difference and the standard error.
- Step 6. Benjamini-Hochberg adjusted p-values less than 0.05 (controlling the false discovery rate at 5%) were considered to be significant

*Robustness analysis.*

In order to increase robustness of the resulting meta-signature, a leave-one-out analysis was conducted by repeating the meta-analysis 12 times, leaving out one of the datasets in each round. The final meta-signature consisted of all genes that were differentially expressed in all 12 meta- analyses.

**Characteristics of data analysis**

| **Dataset** | **Dual channel** | **Data** | **Pre-processing** | **R package** | **Probe annotation** | **Comments** |
| --- | --- | --- | --- | --- | --- | --- |
| Dataset 1  (Centlow ^4^) | Yes | Raw | Background correction: normexp; within-array normalization: loess | Limma | Retrieved from original authors |  |
| Dataset 2  (Enquobahrie ^5^) | Yes | Pre-processed | Pre-processed data was retrieved from original authors (loess within-array normalization) | - | See Dataset 1 | KNN imputation^6^  was used to impute missing expression values |
| Dataset 3  (Hoegh ^7^) | No | Raw | RMA^8^ | Affy | Package hgu133a.db |  |
| Dataset 4  (Jebbink ^9^ ) | No | Raw | Summarization; normalization: NEQC^10^ | Beadarray | Package illuminaHumanv4.db |  |
| Dataset 5  (Kivinen ^11^) | No | Raw | RMA^8^ | Affy | Package hgu133plus2.db |  |
| Dataset 6  (Meng ^12^) | No | Probe-level | NEQC^10^ | Limma | Package illuminaHumanv4.db |  |
| Dataset 7  (Nishizawa ^13^) | Yes | Pre-processed | Pre-processed data was retrieved from GEO (within-array loess normalization) | - | Package hgug4112a.db |  |
| Dataset 8  (Nishizawa ^14^) | No | Raw | RMA^8^ | Oligo | Package hugene10sttranscriptcluster.db |  |
| Dataset 9  (Sitras ^15^) | No | Pre-processed | Pre-processed data was retrieved from GEO (quantile normalization) | - | Updated original annotation available on GEO (GPL2986) by BLASTing probe sequences (AB1700 Human Array V2 Probe Sequences were obtained from Life Technologies Benelux). Only probes with unique gene matches were used to replace the original annotation |  |
| Dataset 10  (Tsai ^16^) | No | Probe-level | NEQC^10^ | Limma | Package illuminaHumanv2.db | Treated the data as 2 separate datasets: 10A and 10B |
| Dataset 11  (Winn ^17^) | No | Raw | RMA^8^ | Affy | Package hgu133a.db |  |

1. Ramasamy A, Mondry A, Holmes CC, Altman DG. Key issues in conducting a meta-analysis of gene expression microarray datasets. *PLoS Med*. 2008;5:e184

2. Choi JK, Yu U, Kim S, Yoo OJ. Combining multiple microarray studies and modeling interstudy variation. *Bioinformatics*. 2003;19:i84-i90

3. Hedges L, Olkin I. *Statistical Methods for Meta-Analysis*. Orlando: Academic Press; 1985.

4. Centlow M, Wingren C, Borrebaeck C, Brownstein MJ, Hansson SR. Differential gene expression analysis of placentas with increased vascular resistance and pre-eclampsia using whole-genome microarrays. *J Pregnancy*. 2011;2011:472354

5. Enquobahrie DA, Meller M, Rice K, Psaty BM, Siscovick DS, Williams MA. Differential placental gene expression in preeclampsia. *Am J Obstet Gynecol*. 2008;199:566.e561-511

6. Troyanskaya O, Cantor M, Sherlock G, Brown P, Hastie T, Tibshirani R, Botstein D, Altman RB. Missing value estimation methods for DNA microarrays. *Bioinformatics*. 2001;17:520-525

7. Hoegh AM, Borup R, Nielsen FC, Sorensen S, Hviid TV. Gene expression profiling of placentas affected by pre-eclampsia. *J Biomed Biotechnol*. 2010;2010:787545

8. Irizarry RA, Hobbs B, Collin F, Beazer-Barclay YD, Antonellis KJ, Scherf U, Speed TP. Exploration, normalization, and summaries of high density oligonucleotide array probe level data. *Biostatistics*. 2003;4:249-264

9. Jebbink JM, Boot RG, Keijser R, Moerland PD, Aten J, Veenboer GJ, van Wely M, Buimer M, Ver Loren van Themaat E, Aerts JM, van der Post JA, Afink GB, Ris-Stalpers C. Increased glucocerebrosidase expression and activity in preeclamptic placenta. *Placenta*. 2015;36:160-169

10. Shi W, Oshlack A, Smyth GK. Optimizing the noise versus bias trade-off for Illumina whole genome expression BeadChips. *Nucleic Acids Res*. 2010;38:e204

11. Kivinen K, Peterson H, Hiltunen L, Laivuori H, Heino S, Tiala I, Knuutila S, Rasi V, Kere J. Evaluation of STOX1 as a preeclampsia candidate gene in a population-wide sample. *Eur J Hum Genet*. 2007;15:494-497

12. Meng T, Chen H, Sun M, Wang H, Zhao G, Wang X. Identification of differential gene expression profiles in placentas from preeclamptic pregnancies versus normal pregnancies by DNA microarrays. *Omics*. 2012;16:301-311

13. Nishizawa H, Pryor-Koishi K, Kato T, Kowa H, Kurahashi H, Udagawa Y. Microarray analysis of differentially expressed fetal genes in placental tissue derived from early and late onset severe pre-eclampsia. *Placenta*. 2007;28:487-497

14. Nishizawa H, Ota S, Suzuki M, Kato T, Sekiya T, Kurahashi H, Udagawa Y. Comparative gene expression profiling of placentas from patients with severe pre-eclampsia and unexplained fetal growth restriction. *Reprod Biol Endocrinol*. 2011;9:107

15. Sitras V, Paulssen RH, Gronaas H, Leirvik J, Hanssen TA, Vartun A, Acharya G. Differential placental gene expression in severe preeclampsia. *Placenta*. 2009;30:424-433

16. Tsai S, Hardison NE, James AH, Motsinger-Reif AA, Bischoff SR, Thames BH, Piedrahita JA. Transcriptional profiling of human placentas from pregnancies complicated by preeclampsia reveals disregulation of sialic acid acetylesterase and immune signalling pathways. *Placenta*. 2011;32:175-182

17. Winn VD, Gormley M, Paquet AC, Kjaer-Sorensen K, Kramer A, Rumer KK, Haimov-Kochman R, Yeh RF, Overgaard MT, Varki A, Oxvig C, Fisher SJ. Severe preeclampsia-related changes in gene expression at the maternal-fetal interface include sialic acid-binding immunoglobulin-like lectin-6 and pappalysin-2. *Endocrinology*. 2009;150:452-462
